# Iron salt-promoted oxidation of steroidal phenols by *m*-chloroperbenzoic acid: a route to possible antitumor agents†

**DOI:** 10.1039/d2ra03717c

**Published:** 2022-07-18

**Authors:** Tatjana J. Kop, Nataša Terzić-Jovanović, Željko Žižak, Bogdan A. Šolaja, Dragana R. Milić

**Affiliations:** University of Belgrade, Institute of Chemistry, Technology and Metallurgy, Department of Chemistry Njegoševa 12 11000 Belgrade Republic of Serbia tatjana.kop@ihtm.bg.ac.rs; University of Belgrade, Institute for Oncology and Radiology of Serbia Pasterova 14 11000 Belgrade Serbia; University of Belgrade, Faculty of Chemistry Studentski trg 12-16 11158 Belgrade Serbia; Serbian Academy of Sciences and Arts Knez Mihailova 35 11000 Belgrade Serbia

## Abstract

A new oxidant, containing *m*-chloroperoxybenzoic acid (MCPBA) and an iron salt, was developed and used for oxidation of steroidal phenols to a quinol/epoxyquinol mixture. Reaction was optimized for estrone, by varying initiators (Fe-salts), reaction temperature, time and mode of MCPBA application. A series of five more substrates (17β-estradiol and its hydrophobized derivatives) was subjected to the optimized oxidation, providing corresponding *p*-quinols and 4β,5β-epoxyquinols in good to moderate yields. The obtained epoxyquinols were additionally transformed by oxidation, as well as the acid-catalyzed oxirane opening. In a preliminary study of the antiproliferative activity against human cancer cell lines, all newly synthesized compounds expressed moderate to high activity.

## Abstract 2

Iron salt-promoted reaction of estrone and its derivatives with *meta*-chloroperoxybenzoic acid was developed and epoxyquinols were further transformed. Most compounds showed *in vitro* antiproliferative activity.

## Graphical abstract

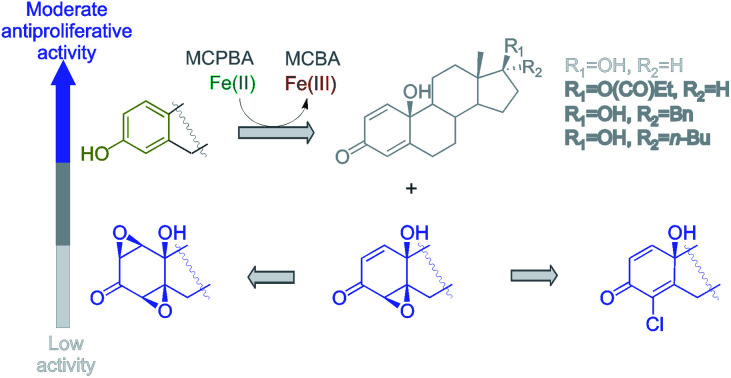

## Introduction

*para*-Quinols and epoxyquinols represent two biologically important classes of compounds. A number of biologically active natural products with *p*-quinol substructure were isolated from quite different sources.^1–5^ Epoxyquinols, whether natural or synthetic, also exhibit a wide range of activities.^6–11^ Smaller phenols can form *p*-quinols with a number of non-metal oxidants, such as oxone^12^ or hypervalent iodine(iii) reagents.^13,14^ Oxidation of several phenol ethers with DAIB and PIFA was used for the synthesis of a series of antitumor quinols.^15^ It was also known that phenols can be oxidized by various oxidants in the presence of Fe(ii)^16^ and Fe(iii)^17^ salts, usually *via* intermediate phenoxy radical.^18^ As assumed, the neuroprotective role of phenols in living cells is accomplished by oxidative quenching of hydroxyl radicals, products of ferric-assisted degradation of hydroperoxides in cytochrome P450.^19^ In this process, smaller phenols yield mixtures of epoxyquinols, while estrogens produce mixtures of *p*-quinols, 6-hydroxylated quinols and *syn*-1,2-epoxyquinols. Furthermore, estrogens exhibit neuroprotective roles in several unfavorable oxidative processes through estrogen receptor dependent or independent free radical scavenging.^20^ Steroidal *p*-quinols, which have long been known as metabolites of estrogens,^21^ have no binding affinity for estrogenic receptors. The reversible reduction reaction of *p*-quinol to estrone is an enzymatically controlled, ROS (reactive oxygen species) free process.^22^ Due to this process, *p*-quinols act as prodrugs for active estrogens. Therefore, DHED (*p*-quinol derived from 17β-estradiol) is a promising brain targeting prodrug of 17β-estradiol for hormone therapy without peripheral side-effects.^23^ Its epimer, α-DHED (derived from 17α-estradiol) has an even higher brain selectivity for targeted neurotherapy.^24^ Several steroidal *p*-quinols are used as prodrugs of antioxidants^25^ and ophthalmic agents^26^ and in estrogen replacement therapy.^27^ Until then, only a few methods for the synthesis of steroidal *p*-quinols were published.^28–30^ So far, the most efficient methods for direct oxidation of unprotected estradiol to *p*-quinol used KMnO_4_/HCl system,^31^ and lead(iv) acetate assisted by microwave irradiation.^32^

Our first attempt to incorporate a cyclohexadienone substructure into a steroidal skeleton resulted in a novel method of oxidation of polycyclic phenols by the MCPBA/(BzO)_2_/*hν* system.^33,34^ Both groups of obtained compounds, steroidal *p*-quinols and 4β,5β-epoxyquinols, exhibited the antitumor activity.^35,36^ In addition, they also proved to be good synthetic intermediates for many A-ring polyfunctionalized compounds, including quinones,^34,37^ diepoxides,^36,38^ bromo-derivatized quinols and epoxyquinols,^38^ rearomatized^39^ and A,B-spiro structures.^36^ All of them expressed moderate to significant antitumor activity, as well.

Willing to investigate other synthetic approaches to such useful compounds, we developed a new oxidation system and investigated it extensively. In this work, we present the oxidation of estrone (1a) and its five derivatives (of which three have an unprotected 17β-hydroxy group) with *meta*-chloroperoxybenzoic acid (MCPBA) in the presence of iron salts. The influence of different iron salts and reaction conditions on the reaction efficiency and distribution of products was investigated. Based on those results, a modified procedure for oxidation of steroidal phenols was optimized, producing steroidal *p*-quinols 2 and epoxyquinols 3 in good overall yields under mild reaction conditions.

## Results and discussion

### Oxidation of steroidal phenols by Fe(ii)/Fe(iii)-MCPBA system

During our previous extensive work on optimization of the oxidation of steroidal phenols by the system MCPBA/(BzO)_2_/*hν*, several unfavourable facts opened up the possibility for further investigation. Control experiments, studying reaction conditions, showed sensitivity to air and moisture and the necessity of the presence of benzoyl peroxide combined with irradiation of the reaction mixture. Thus, possibility of using photosensitive substrates under above conditions is excluded. On the other hand, clean reaction mixtures were obtained, allowing easy separation of the product.^33^ Under any of applied conditions, lactones as possible Baeyer–Villiger reaction products were not detected. The reaction also took place in the presence of magnesium monoperphthalate (MMPP) as an oxidant and azobisisobutyronitrile (AIBN) as initiator, but the best results were achieved with the combination MCPBA/(BzO)_2_. Peroxyacetic acid did not react under applied conditions. Monitoring the fate of estrone in reaction conditions without initiators and irradiation, under day light and prolonged reflux (26 hours), 30% conversion was observed. Products were quinol 2a and epoxyquinol 3a in 1 : 2 ratio. This fact led us to a conclusion that traces of aroyloxy radical, presented in the reaction mixture due to decomposition of MCPBA, still can promote the reaction. That also supported our previous conclusion that aroyloxy radical propagated reaction, while irradiation is necessary only for initiation by benzoyl peroxide. It was the starting point in our efforts to improve the reaction system during this work. In aim to find an alternative way to initiate the reaction, *i.e.*, to generate aroyloxy radical in amount acceptable for the efficient propagation of the reaction, avoiding use of benzoyl peroxide as initiator, as well as irradiation, several test reactions were done. The use of Fe-salts instead of (BzO)_2_ seemed reasonable since Fe(ii)-ions could also promote the cleavage of the peroxy bond. At the same time, there was a need to test at least two more peroxide reagents under new conditions (hydrogen peroxide, as the cheapest and most widely used source of hydroxy radical, and MMPP as a source of another aroyloxy radical), to determine is the aroyloxy radical indeed a key intermediate in the propagation of the reaction. Oxidation of estrone was performed under Fenton and Fenton-like conditions (meaning Fe(ii) or Fe(iii) ions, respectively, accompanied with H_2_O_2_), examining whether hydroxy radical can provoke reaction, too. Reaction was performed with and without irradiation, in several solvent systems (dried, as well as not) and all cases showed traces of desired products, but with low conversion of estrone (not isolated, confirmed only by comparative TLC analysis). Having in mind that different peroxy reagents showed very opposite results in previously designed oxidation system, and that only aromatic peracids reacted in satisfying manner, research was not extended to new oxidants, but conditions were adjusted by combining aromatic peracid reagents (MCPBA and MMPP) with iron ions as initiators. Although several solvents were tested, acetone was one of choice, as well as MCPBA over MMPP as an oxidant choice. Also, reaction took place without irradiation. Reaction occurred in acetone/water mixture, but precipitation of the estrone significantly lowered the conversion. So, further reactions were done in pure acetone, with MCPBA as oxidant, and without irradiation. Given the complexity of Fe-ion/hydroperoxide chemistry, which includes the Fe(iii)/Fe(ii) redox couple, Fe(ii)- and Fe(iii)-salts were used sparingly. Previous results have shown that optimal yields were achieved with 3-fold excess of MCPBA relative to phenolic substrate.^33,37^ Consequently, our synthetic strategy was based on the oxidation of steroidal phenols with 3 mol equivalents of MCPBA, with initiation by different Fe-salts. In addition, variations of counterions, reaction time, temperature, as well as the mode of application of the peroxyacid were also included in the research set-up. The yield and distribution of products were followed and obtained results are summarized in Table 1.

**Table 1:** Reaction of estrone (1a) with 85% MCPBA in the presence of various Fe-salts in acetone

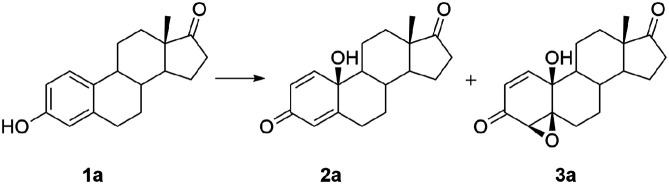									
Run	Fe-Salt	Fe^*n*+^ : 1a (eq.)	MCPBA	*T* (°C)	*t* (h)	Yield^a^ (%)			
						1a	2a	3a	2a + 3a
1	FeSO_4_	1 : 1	3 × 1 eq.	56	4	5	43	47	90
2	FeSO_4_	0.2 : 1	3 × 1 eq.	56	4	40	29	28	57
3	FeSO_4_	1 : 1	1 × 3 eq.	56	4	15	36	22	58
4	FeSO_4_	0.2 : 1	1 × 3 eq.	56	4	49	33	17	50
5	FeSO_4_	1 : 1	1 × 3 eq.	20	24	19	37	18	55
6	(NH_4_)_2_Fe(SO_4_)_2_	1 : 1	3 × 1 eq.	56	5	7	36	32	68
7	(NH_4_)_2_Fe(SO_4_)_2_	0.2 : 1	1 × 3 eq.	56	7	25	39	25	64
8	K_4_[Fe(CN)_6_]	1 : 1	3 × 1 eq.	56	5	10	20	13	33
9	K_4_[Fe(CN)_6_]	1 : 1	1 × 3 eq.	20	24	57	17	16	33
10	FeCl_2_	1 : 1	3 × 1 eq.	56	2.5	—	—	—	—b
11	FeCl_2_	0.2 : 1	1 × 3 eq.	56	5	54	25	11	36
12	Fe_2_(SO_4_)_3_	1 : 1	3 × 1 eq.	56	4	17	29	11	40
13	Fe_2_(SO_4_)_3_	0.2 : 1	3 × 1 eq.	56	4	39	31	29	60
14	Fe_2_(SO_4_)_3_	0.2 : 1	1 × 3 eq.	56	4	54	29	16	45
15	Fe_2_(SO_4_)_3_	1 : 1	1 × 3 eq.	20	24	56	28	6	34
16	Fe_2_(SO_4_)_3_ × (NH_4_)_2_SO_4_	0.2 : 1	1 × 3 eq.	56	4	25	43	19	62
17	Fe_2_(SO_4_)_3_ × (NH_4_)_2_SO_4_	0.2 : 1	1 × 3 eq.	56	7	12	33	31	64
18	K_3_[Fe(CN)_6_]	1 : 1	3 × 1 eq.	56	4	45	30	24	54
19	K_3_[Fe(CN)_6_]	0.2 : 1	1 × 3 eq.	56	5	45	30	20	50
20	K_3_[Fe(CN)_6_]	1 : 1	1 × 3 eq.	20	24	48	35	11	46
21	FeCl_3_	1 : 1	3 × 1 eq.	56	2.5	—	—	—	—b
22	FeCl_3_	0.2 : 1	1 × 3 eq.	56	5	20	2	6	8

a Isolated yields; yields of unconverted estrone is also included, since conversion is incomplete; the rest up to 100% remained adsorbed on the column as a complex mixture of polar products.

b Complex mixture of products-not isolated.

In all performed experiments, the same product mixture, consisting of quinol 2a and *syn*-epoxyquinol 3a, was formed. Their yield and distribution varied with initiators and reaction conditions. The mode of MCPBA application showed the major influence on reaction efficacy (total yield). As can be seen from Table 1, better total yield, together with a significant increase of the epoxide fraction in the product mixture, was achieved when the peracid was added in portions (runs 1 *vs.* 3, 2 *vs.* 4, and 13 *vs.* 14), except in the presence of complex Fe-salts where the mode of application showed no influence on reaction efficacy. The counter ion of the applied initiator was important only in the case of chloride salts, where a low yield of target compounds (run 22), or even the formation of a complex mixture of inseparable products (runs 10 and 21) was obtained, probably due to competitive reactions. The effect of Fe-ion oxidation state on reaction efficacy proved to be irregular and could not be simply generalized – Fe(iii) complex salts were mainly more efficient than Fe(ii) ones (runs 18 *vs.* 8, and 20 *vs.* 9), while in the case of simple salts, both the opposite effect (runs 1 *vs.* 12, 5 *vs.* 15, and 11 *vs.* 22) and quite similar results with Fe(ii) and Fe(iii) salts (runs 2 *vs.* 13, 4 *vs.* 14, and 10 *vs.* 21) were observed. The amount of Fe(iii) ions did not affect the yield of products (run 12 *vs.* 13), as it was in the case of Fe(ii) ions (run 1 *vs.* 2). In addition, the insolubility of iron salts in acetone causes reaction to take place under heterogenous conditions, so the solid phase surface area and activity could also affect the overall yield and products distribution.

Finally, in the optimal experiment to a refluxing acetonic suspension of estrone (1 eq.) and FeSO_4_ (1 eq.) MCPBA was added in portions (3 × 1 eq.), with 30 minutes interval between them, and reaction mixture was heated for additional 4 h. After simple work-up, a clean reaction mixture was easily separated by a dry-flash column chromatography on silica-gel, leading to the highest overall yield of products (90%), in a ratio of quinol to epoxyquinol of nearly 1 : 1 (Table 1, run 1). The rest of the reaction mixture consisted of unreacted estrone (5%) and inseparable mixture of polar products strongly adsorbed on silica during the chromatography.

**Scheme 1:**
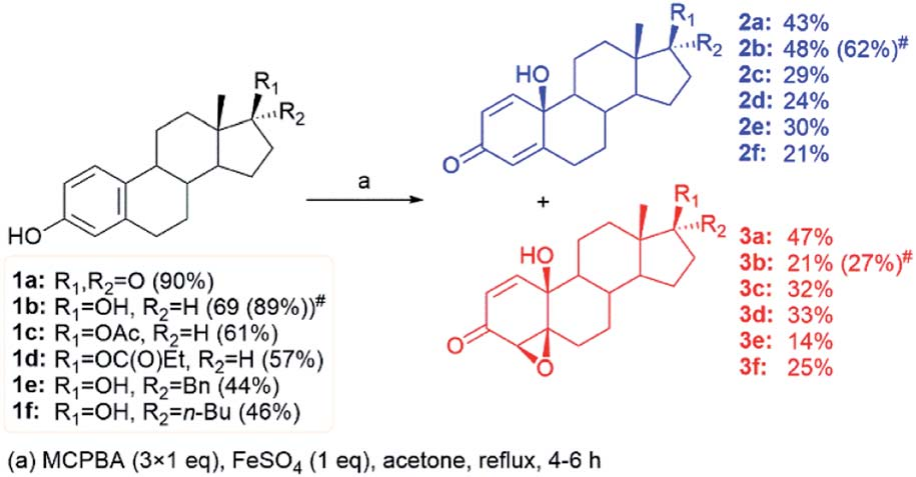
Oxidation of steroidal phenols 1 into corresponding quinol/epoxyquinol mixtures (^#^incomplete conversion; yields recalculated to the amount of converted substrate are shown in parentheses).

A series of five more steroidal phenols derived from estrone, including compounds with unprotected hydroxyl-groups, as well as the ester subunits, were subjected to optimized oxidation (Scheme 1). As with estrone (1a), each reaction led to formation of only two products – *para*-quinol 2 and 4β,5β-epoxyquinol 3 (although theoretically *p*-quinol could be transformed to four epoxyquinols, with different, α- or β-orientation at different, 1,2- and 4,5-positions).

In the presence of unprotected hydroxy-, and ester moieties, as well as aromatic and alkyl side chains, the reaction proceeded in satisfactory yield, as shown in Scheme 1. Total yields of quinols and epoxyquinols ranged from 44% for 17α-benzyl-17β-estradiol (1e) up to 90% for estrone (1a), while their distribution varied from 1 : 1 to 2 : 1 approximately.

### Studying reaction mechanism by the EPR

**Scheme 2:**
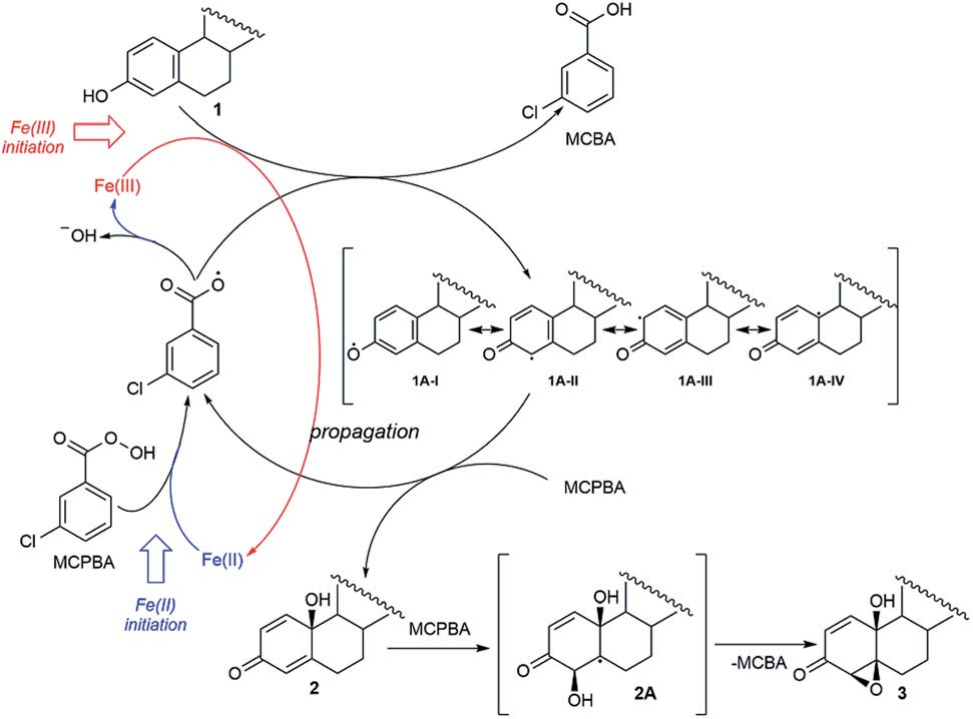
Proposed mechanism of oxidation by Fe(ii)/Fe(iii)-MCPBA system.

**Fig. 1:**
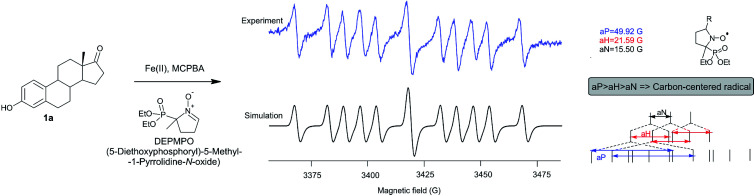
EPR spectrum of the reaction of estrone with MCPBA in the presence of Fe(ii) ions and DEPMPO as a radical trapper.

The fact that the regio- and stereoselectivity is preserved and manifested in the same manner in all reactions of estrone in three different systems (MCPBA/(BzO)_2_/*hν*, MCPBA/Fe(ii) and MCPBA/Fe(iii)), leads us to a conclusion that the reacting species responsible for the propagation phase are the same for all three reaction. So, the initiating step of this reaction, which includes the formation of an aroyloxy radical, makes the main difference in applicated oxidizing systems (Scheme 2). Instead of homolytic photochemical cleavage of benzoyl-peroxide (as proposed^40^ for the system MCPBA/(BzO)_2_/*hν*), aroyloxy radical is formed directly from MCPBA by reductive cleavage of the peroxy bond by Fe(ii) ion (Scheme 2, blue path). According to previous theoretical findings,^40^ attack of the *m*-Cl-C_6_H_4_COO˙ radical on phenolic hydroxy group is highly favorable, exothermic process, with no needed activation energy, indicating that it is very fast. EPR spectra of Fe^2+^ induced reaction of estrone with MCPBA in the presence of DEPMPO indicated the existence of only the C-centered radical (Fig. 1), which is in accordance with assumption that *m*-Cl-C_6_H_4_COO˙ radical reacts with estrone in the same manner under newly applied conditions, yielding resonantly stabilized radical 1A (Scheme 2, black cycle – first step). The next step of the reaction, approach and abstraction of hydroxy radical from MCPBA, took place exclusively in position 10 (the most stabilized resonant structure is 1A-IV), from the thermodynamically strongly favored β-side,^40^ yielding *p*-quinol 2 and the second aroyloxy radical, a source for a new reaction cycle.

Interestingly, Fe(iii) ions also initiated a reaction with estrone without irradiation. It can be assumed that the reaction with Fe(iii) starts with the direct one electron oxidation of estrone and the formation of a radical 1A (Scheme 2, red path). The reaction propagates according to the reaction path indicated for systems Fe(ii)/MCPBA and MCPBA/(BzO)_2_/*hν*. An almost imperceptible difference (meaning overall yield and product ratio) between the reactions performed with stoichiometric and catalytic amounts of Fe(iii) supports presumption that the initiating step, oxidation of estrone by Fe(iii), is the slowest one and at some point of the reaction is suppressed by aroyloxy radical coming from fast reaction of the MCPBA with 1A. Therefore, starting amount of Fe(iii) does not affect the efficiency of the reaction to the same extent as the starting amount of Fe(ii)-ions as initiators, which contribute to the amount of *m*-Cl-C_6_H_4_COO˙ by direct reaction with MCPBA. The proximity of the β-hydroxyl group in position 10 directed a further oxygenation of *p*-quinol from β-side. The abstraction of hydroxy-radical from MCPBA *via* homolytic cleavage of double bond 4,5 produced a thermodynamically favored C-radical 2A and, followed by ring closure, 4,5-epoxyde rather than 1,2-isomer.^40^ Conversion of *p*-quinol to 4β,5β-epoxyquinol (like for previous reaction system) was confirmed experimentally, subjecting it under the same Fe(ii)/Fe(iii)-MCPBA conditions.

### Comparison of Fe(ii)/Fe(iii)-MCPBA *vs.* MCPBA/(BzO)_2_/*hν* system

Both reaction systems showed the same excellent chemo-, regio- and stereoselectivity (with exception of reactions done with iron chlorides) and were favored by the same type of peroxy reagent. Also, changes of the reaction time and amount of the MCPBA affected similarly the outcome of the reaction. Products and unreacted substrate were easily separated from the reaction mixture. In both cases the only remain was a mixture of polar products, with no synthetic use. Unreacted substrate is reusable. Under both applied systems quinol 2a was transformed to epoxyquinol 3a in yield close to 50%. Main advantages of newly developed system over previous one are insensitivity to moisture and air, applicability to photosensitive and substrates with unprotected –OH groups, easier work-up of the raw reaction mixture and use of environmentally more acceptable solvent and initiator. Shortcomings of new reaction are higher sensitivity to mode of application of MCPBA and larger amount of initiator used to achieve good efficiency. In both systems recovery of the initiator is very low (5% for (BzO)_2_ and 0% for any of iron salts).

### Synthetic utility: structural modifications of epoxyquinols

**Scheme 3:**
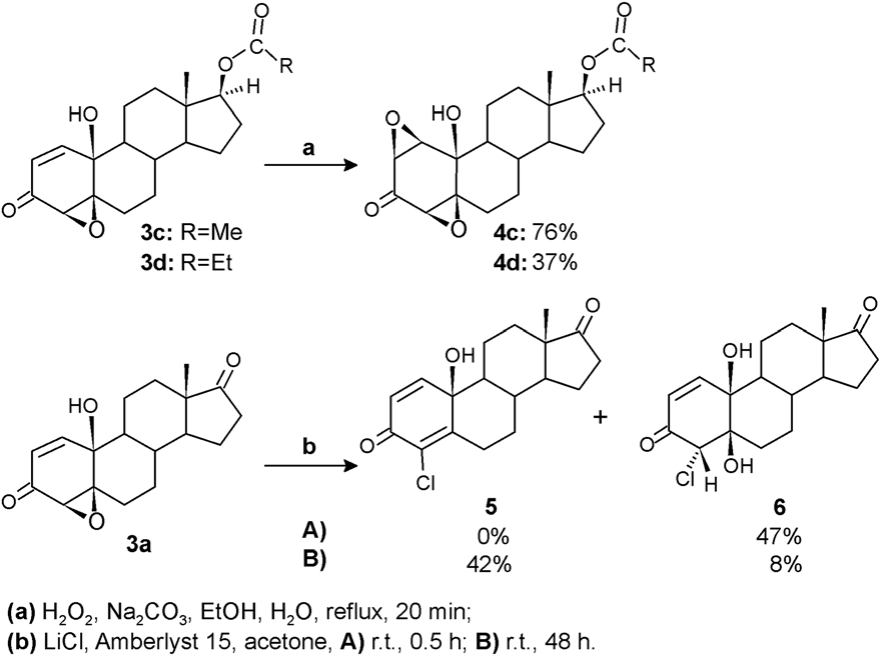
Transformations of epoxyquinols.

Our previous results demonstrated high antitumor activity of various steroidal polyoxygenated compounds, including A-ring *syn*-diepoxide with 10-hydroxy and 17-keto functionalities.^38^ Willing to examine the influence of structural modifications on antitumor activity and cytotoxicity more in detail, selected epoxyquinols were additionally transformed into three new compounds. The terminal subunits of steroidal framework – D and A rings – were chemically altered for this purpose (Scheme 3).

Oxidation by hydrogen peroxide under basic conditions introduced a second oxirane ring in epoxyquinols 3c and 3d to give diepoxides 4c and 4d, respectively, by already known synthetic transformation, applied and investigated for synthesis of analogous 17-keto diepoxyde.^38^ Modifications of the A-ring were achieved by the acidic oxirane ring opening of epoxyquinol 3a using the LiCl/Amberlyst 15 system. Applied mild reaction conditions provided corresponding chloroquinol 5 as the main product. It is important to note that unlike the previous synthesis of analogous 4-bromoquinol,^38^ where intermediate bromohydrin was not detected, chlorohydrin 6 expressed a significant degree of stability under the same reaction conditions. So, by shortening the reaction time, it could be isolated as the only product, while prolonging reaction time to 48 h resulted in its almost complete dehydration to chloroquinol 5.

### Structural characterization

All compounds were characterized by UV-vis, FTIR, ^1^H, ^13^C NMR and HR mass spectrometry. Additional structural elucidation was performed by 2D NMR experiments (COSY, HSQC, HMBC and NOESY) and by comparison of collected data with those of known compounds. All compounds gave correct elemental analysis, in agreement with proposed molecular formulae.

All synthesized *p*-quinols expressed strong UV absorption around 230–250 nm due to conjugated carbonyl group at C-3 position. Weaker absorption at the similar wavelengths was observed for the corresponding epoxyquinols, while diepoxides were UV inactive compounds. The band around 280 nm, appeared in UV spectra of compounds 2e and 3e, indicated the presence of 17α-benzyl group.

IR spectra of *p*-quinols and corresponding epoxides contain strong wide maxima at 3100–3000 cm^−1^ (O–H), strong sharp maxima originated from conjugated C=O, at 1660–1670 cm^−1^ (quinols) and 1680–1690 (epoxyquinols) and at 1610–1635 cm^−1^ (due to conjugated C=C, much weaker for epoxyquinols than for quinols). IR spectra of diepoxides also contain wide maxima at 3100–3000 cm^−1^, but the characteristic carbonyl band is shifted at higher values (1710–1730 cm^−1^), due to saturation of the A-ring.

**Fig. 2:**
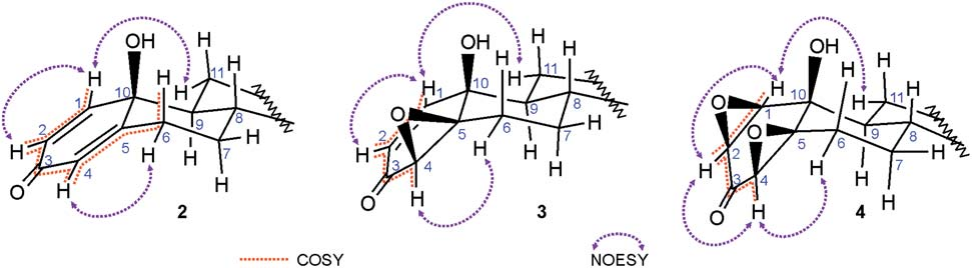
H,H-COSY and NOE correlations in quinols 2, epoxyquinols 3 and diepoxides 4. COSY couplings: vinylic (H–C(1)–H–C(2)), W (H–C(2)–H–C(4)) and allylic (H–C(4)–H_β_–C(6)).

In the ^1^H NMR spectra of all *p*-quinols a typical d–dd–t pattern of the A-ring dienone subunit^37^ was found: a doublet at 7.0–7.3 ppm, with coupling constant around 10 Hz, originating from proton attached to C-1, coupled with *cis*-vinyl H–C(2); doublet of doublets at 6.1–6.2 ppm belonging to H–C(2), coupled with H–C(1) and H–C(4), with coupling constants around 10 and 2 Hz, indicating coplanar W-geometry of H–C(1)–C(3)–C(4)–H segment; doublet of doublets belonging to H–C(4), appearing as an irregular triplet at 5.9–6.0 ppm, with coupling constants of 2 and 1 Hz, long-range coupled with H–C(2) (W-coupling) and allylic H_β_–C(6) (Fig. 2). An axial orientation of H_β_–C(6) enables maximal overlapping of π-C(4)–C(5) and σ-C(6)–H orbitals and long-range allylic coupling. Several 2D NMR data contributed A-ring assignments: scalar couplings were confirmed from COSY correlations; NOESY correlations between H–C(1) and H_α_–C(11) and H_α_–C(4)–H_α_–C(6) confirmed their spatial proximity (Fig. 2); correct HSQC correlations with carbon signals at expected shifts confirmed position of protons. H_β_–C(6) gave doublet of doublet of doublet of doublets (appearing as triplet of doublet of doublets) at ∼2.8 ppm, which indicates two strong couplings (geminal and *anti*-diaxial with H_α_–C(7)), one moderate (*gauche*) with H_β_–C(7) and weak allylic with H_α_–C(4).

Strong coupling (COSY correlation) and shared carbon with H_β_–C(6) at ∼32 ppm (HSQC correlation), alongside with NOESY correlation with H_α_–C(4), indicated that doublet of doublets of doublets at 2.3–2.4 ppm belongs to H_α_–C(6). Signals at ∼1.0 ppm and ∼2.0 ppm were assigned to H_α_–C(7) and H_β_–C(7), respectively, regarding the COSY, HMBC and NOESY correlations to adjacent protons attached to C(6) and C(8), and correlation of both signals with one carbon signal at ∼33 ppm in HSQC spectrum. Corresponding signals of protons attached to C(11) and C(12) were assigned in the similar way, having in mind coupling with H_α_–C(9) and NOESY correlation of H_α_–C(11) with H–C(1). Correlations with H–C(14) enabled assignment of protons in position 15, and consequently, in position 16. ^1^H NMR spectra of 4β,5β-epoxyquinols also contain characteristic signals of vinylic protons: doublet at ∼6.7 ppm for H–C(1) and doublet of doublets for H–C(2) at ∼5.8 ppm. Oxirane proton attached to C(4) gives doublet on lower chemical shift (∼3.3 ppm), comparing to quinol, without coupling with H–C(6). Saturation of 4–5 bond led to the absence of H–C(4)–H_β_–C(6) coupling and to lower chemical shifts of protons in position 6, as well. Although β-orientation of the epoxy moiety could not be doubtlessly determined from observed H–C(4)–H_α_–C(6) NOE correlation^35^ (Fig. 2), it was verified from further transformation of epoxyquinols 3 to *syn*-diepoxyalcohols 4. Similar chemical shifts of protons in positions 4 and 6 were noticed for diepoxides, but ^1^H NMR spectra and ^13^C NMR spectra of diepoxides are also characterized by total loss of the vinylic signals. Signals of H–C(1), H–C(2) and H–C(4) are between 3.0 and 4.0 ppm, with significantly lower *J*_1,2_ (∼4 Hz), comparing to those of corresponding *p*-quinols and epoxyquinols. The presence of the W-coupling between H–C(2) and H–C(4) indicated their coplanar position. The NOE correlations of H–C(1) with H_α_–C(11), and H–C(4) with H_α_–C(6) (Fig. 2) indicated α-orientation of H–C(1) and H–C(4), and due to H-(2/4) coplanarity of H–C(2), as well. Consequently, both oxirane rings adopted β-orientation, as it was found earlier for 17-keto analog.^36,38^

### Bio-utility: antiproliferative activity

**Fig. 3:**
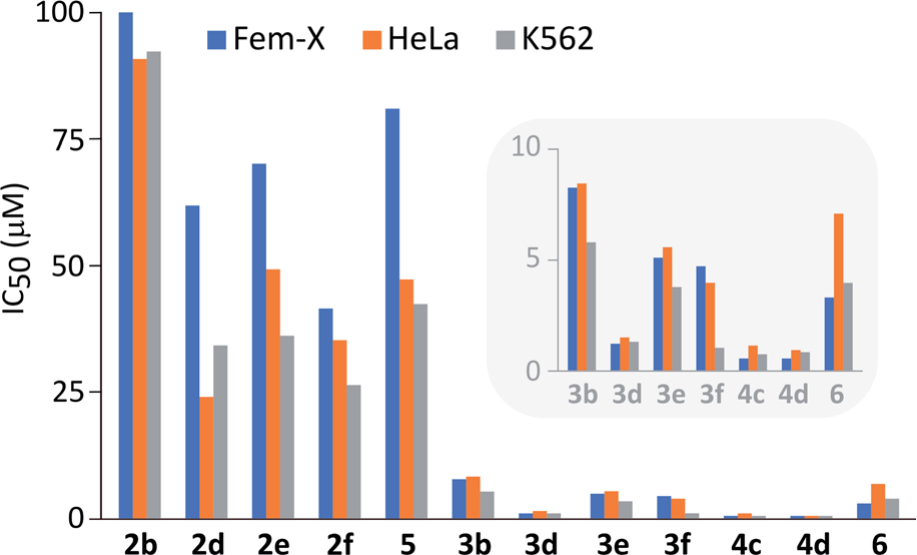
Antiproliferative activity of newly synthesized compounds against human malignant melanoma (Fem-X), human cervix carcinoma (HeLa) and human myelogenous leukemia (K562) cell lines. Activities of more potent samples are accentuated in the inset.

It is important to note that performed structural changes of steroidal phenols induced changes in the type of biological activity, providing estrogen-based compounds with an antiproliferative action toward three human cancer cell lines (Fig. 3 and Table S1†).

All compounds expressed dose-dependent and phenotype-independent activity. Quinol 2b proved to be completely inactive, but modification of D-ring substituents, as well as the introduction of chlorine into the quinol subunit led to an increase in the antineoplastic activity, especially against human cervix carcinoma (HeLa) and human myelogenous leukemia (K562) cells (2b*vs.*2d,e,f and 5). However, a moderate activity was achieved employing quinols. On the other hand, their further oxidation to epoxyquinols and diepoxides resulted in the significant increase of the antiproliferative activity against all three tested cell lines (2*vs.*3/4). The influence of oxirane fragment was not found to be additive, since the first epoxidation provoked much higher activity increase (2d*vs.*3d) in comparison to the second one (3d*vs.*4d). In addition, quite good antiproliferative activity was preserved even after the opening of the epoxy-ring by chloride (Fig. 2, compound 6). At the same time, there is a noticeable increase in activity of quinols and epoxyquinols with 17β-propioniloxy- (2d and 3d) and 17α-*n*-butyl-substituents (2f and 3f), relative to other members of two series. Diepoxides 4c and 4d showed IC_50_ values less than 1 μM in all cases, except for 4c against HeLa cells, where this value slightly exceeded 1 μM.

## Conclusions

A new oxidation system, consisting of MCPBA and FeSO_4_, was developed and employed for the transformation of steroidal phenols to quinol–epoxyquinol mixtures. Using estrone as the model compound, numerous parameters were extensively examined – the mode of MCPBA application, type of Fe-salt (simple or complex), oxidation state of Fe-ion, reaction time and temperature. Adding the peracid in three equivalent portions proved to be the most important and led to a significant increase in the total yield (90%). Also, the use of chloride salts was found to be substantially unfavorable since complex mixtures of numerous products were formed. The influence of other parameters was not significant to such an extent. The EPR experiments, done in the presence of DEMPO, confirmed the free-radical mechanism of the oxidation *via* C-centered radical species. The utility of the developed MCPBA/FeSO_4_ oxidant was validated through the transformation of six steroidal phenols into quinol–epoxyquinol mixtures in good yields. In addition, the tolerance of free alcoholic OH-group, as well as its esters, should be highlighted. The synthetic utility of obtained epoxyquinols was verified by their oxidative transformation to diepoxides, as well as to chloro-derivatives *via* acid catalyzed opening of the oxirane ring under mild reaction conditions. Finally, it was shown that the developed oxidation system afforded potent candidates for detailed biological examination. All compounds expressed *in vitro* antiproliferative activity towards three cell lines of human cancer, varying from moderate (quinols) to high (epoxyquinols and diepoxides).

## Author contributions

Tatjana J. Kop – conceptualization, investigation (synthesis), methodology, writing (original draft, review and editing); Nataša Terzić-Jovanović – investigation (EPR); Željko Žižak – investigation (antiproliferative activity); Bogdan A. Šolaja – conceptualization, supervision, funding acquisition, writing (review); Dragana R. Milić – supervision, visualization, writing (original draft, review and editing).

## Conflicts of interest

There are no conflicts to declare.

## Supplementary Material

RA-012-D2RA03717C-s001
