# Supplementary material for: Iron salt-promoted oxidation of steroidal phenols by m-chloroperbenzoic acid: a route to possible antitumor agents
Source: RSC Adv. 2022 Jul 18;12(32):20649–55. doi: 10.1039/d2ra03717c (PMC9290445; doi:10.1039/d2ra03717c)
Supplement: RA-012-D2RA03717C-s001 [file RA-012-D2RA03717C-s001.pdf]

Electronic Supplementary Material (ESI) for RSC Advances.
This journal is © The Royal Society of Chemistry 2022

Supplementary material

for

Iron salts promoted oxidation of steroidal phenols by *m*-chloroperbenzoic acid: route to possible antitumor agents

Tatjana J. Kop,[a] Nataša Terzić-Jovanović,[a] Željko Žižak,[b] Bogdan A. Šolaja[c, d] and Dragana R. Milić[d]

[a] *University of Belgrade - Institute of Chemistry, Technology and Metallurgy, National Institute of Republic of Serbia, Njegoševa 12, 11000 Belgrade, Serbia.*

[b] *University of Belgrade - Institute for Oncology and Radiology of Serbia, Pasterova 14, 11000 Belgrade, Serbia.*

[c] *University of Belgrade - Faculty of Chemistry, Studentski trg 12-16, 11158 Belgrade, Serbia.*

[d] *Serbian Academy of Sciences and Arts, KnezMihailova 35, Belgrade, Serbia*

Contents:

1. **Experimental** 1
2. **Table S1:** Antineoplastic activity of newly synthesized derivatives 7
3. **NMR spectra** of synthesized derivatives:
   - 3.1. ^{1}H NMR, ^{13}C NMR, COSY, HSQC, HMBC and NOESY spectra of compound **2b** 8
   - 3.2. ^{1}H NMR, ^{13}C NMR, COSY, HSQC and HMBC spectra of compound **2d** 15
   - 3.3. ^{1}H NMR, ^{13}C NMR, COSY, HSQC and HMBC spectra of compound **2e** 21
   - 3.4. ^{1}H NMR, ^{13}C NMR, COSY, HSQC, HMBC and NOESY spectra of compound **2f** 27
   - 3.5. ^{1}H NMR and ^{13}C NMR spectra of compound **3b** 34
   - 3.6. ^{1}H NMR, ^{13}C NMR, COSY, HSQC, HMBC and NOESY spectra of compound **3d** 36
   - 3.7. ^{1}H NMR and ^{13}C NMR spectra of compound **3e** 43
   - 3.8. ^{1}H NMR, ^{13}C NMR, COSY, HSQC, HMBC and NOESY spectra of compound **3f** 45
   - 3.9. ^{1}H NMR, ^{13}C NMR, COSY, HSQC and HMBC spectra of compound **4c** 52
   - 3.10. ^{1}H NMR and ^{13}C NMR spectra of compound **4d** 58
   - 3.11. ^{1}H NMR, ^{13}C NMR, COSY, HSQC and HMBC spectra of compound **5** 60
   - 3.12. ^{1}H NMR and DEPT spectra of compound **6** 66

1. Experimental Section

General:

Antiproliferative activity testing towards HeLa, Fem-x and K562 cells was done according to previously described protocol.[1] Stock solutions of tested compounds (10 mM in DMSO) were diluted to various final concentrations by nutrient medium. Amount of survived cells (HeLa (cervix carcinoma), Fem-x (melanoma) and K562 (myelogenous leukemia)) was measured in standard 72 h MTT test. Antiproliferative activities were represented as a concentrations of investigated compounds inducing 50% decrease in cell survival (IC50). Experiments were done in triplicates. The HeLa and K562 cell lines was procured from ATCC. The Fem-X cell line was a kind gift from Dr Nikola Vujanovic (University of Pittsburgh).

EPR spectroscopy: the EPR spectra with DEPMPO as a spin trapper were recorded on a Varian E-109 spectrometer. The EPR instrumental settings for experiment were as following: H = 3140 G (center field), microwave frequency = 9.51 GHz, modulation frequency =100 kHz, field sweep = 200 G, modulation amplitude = 2 G, receiver gain 2×10^4, microwave power = 10 mW, time constant = 32 ms, sweep time = 2 min. WINEPR SimFonia, v 1.25, was used for the simulation of DEPMPO generated EPR spectra. During the experiment, mixture of estrone (2 mM), DEPMPO (112.5 mM), Fe(II) salt (2mM, $FeSO_4$ or $FeCl_2$) and MCPBA (6 mM) in acetonitrile (0.5 mL) was subjected to above mentioned conditions and signal was detected between 3 and 5 minutes.

Synthesis: melting points were determined on a Boetius PMHK 05 apparatus and were not corrected. IR spectra were recorded on Perkin-Elmer FT-IR 1725X spectrophotometer. UV spectra were recorded on a Beckman DU-50 and Cintra 40 spectrophotometers. ^{1}H NMR and ^{13}C NMR spectra were recorded on Varian Gemini-200 (at 200 and 50 MHz, respectively), Bruker AM-250 (at 250 and 62.9 MHz, respectively) and Bruker Avance (at 500 and 125 MHz, respectively) spectrometers in the indicated solvent, using TMS as internal standard. Chemical shifts (δ) are expressed in ppm values and coupling contants (J) in Hz. Mass spectra were taken on a Finnigan-MAT 8230 and HRMS spectra were recorded on Agilent Technologies 1200 Series. Elemental analysis was done on Elementar, Vario EL III. Estrone (**1a**) and estradiol (**1b**) are purchased from Merck. Monoesters **1c** and **1d** were synthesized from diesters by selective hydrolysis. Estrone was alkylated with corresponding organometalic compounds (benzylmagnesium chloride and buthyllithium, respectively) to give substrates **1e**[2] and **1f**.[3]

General procedure 1: Oxidation of steroidal phenols with MCPBA/$FeSO_4$ system (**1** → **2** + **3**).

To a suspension of steroidal phenol **1** (1.00 mmol) and $FeSO_4\times7H_2O$ (278.5 mg, 1.00 mmol, 1 eq) in refluxing acetone (20-40 mL) MCPBA (80%, 3×215.0 mg, 3×1.00 mmol, 3 eq) was added portionwise, every 30 minutes. Reaction mixture was additionally refluxed until consumption of the substrate (monitored by TLC using PhMe/EtOAc 7:3). Acetone was evaporated under reduced pressure and the residue resuspended in DCM (80 mL). Solid Na_2CO_3 (50 mg) and $Na_2S_2O_3$ (50 mg) were added to the suspension and stirred for 10 minutes at room temperature. After filtration and evaporation to dryness the crude mixture was purified by dry-flash chromatography on silica.

1a → 2a + 3a

Estrone (**1a**, 500.0 mg, 1.86 mmol), $FeSO_4\times7H_2O$ (518 mg, 1.86 mmol) and MCPBA (3×400.0 mg, 3×1.86 mmol) in acetone (35 mL) were refluxed for 4 h after addition of MCPBA. Elution with PhMe/EtOAc 9:1, 7:3 and 1:1 afforded unreacted estrone **1a** (29.9 mg, 5%), and compounds **3a** (264 mg, 47%) and **2a** (264 mg, 43%), respectively. Their spectroscopic data were in accordance with literature.[4]

1b → 2b + 3b

Estradiol (**1b**, 390 mg, 1.432 mmol), $FeSO_4\times7H_2O$ (400 mg, 1.43 mmol) and MCPBA (3×309 mg, 3×1.43 mmol) in acetone (40 mL) were refluxed for 4 h after addition of MCPBA. Elution with PhMe/EtOAc 8:2, 7:3 and 1:1 afforded unreacted estradiol (**1b,** 88 mg, 23%) and compounds **3b** (91,4 mg, 21% (27% relative to consumed substrate)) and **2b** (198 mg, 48% (62% relative to consumed substrate)), respectively. Spectroscopic data of quinol **2b** were in accordance with literature.[5]

1c → 2c + 3c
Estradiol 17β-acetate (**1c,** 1,00 g, 3.04 mol), $FeSO_4 \times 7H_2O$ (847 mg, 3.04 mmol) and MCPBA (3× 655 mg, 3×3.04 mmol) in acetone (60 mL) were refluxed for 4 h after addition of MCPBA. Elution with PhMe/EtOAc 9:1 afforded unreacted **1c** (110 mg, 11%), while elution with PhMe/EtOAc 8:2 and 7:3 afforded compounds **3c** (350 mg, 32%) and **2c** (320 mg, 29%), respectively. Their spectroscopic data were in accordance with literature.4

1d → 2d + 3d
Estradiol 17β-propionate (**1d**, 100 mg, 0.29 mmol), $FeSO_4 \times 7H_2O$ (80 mg, 0.29 mmol) and MCPBA (3×63 mg, 3×0.29 mmol) in acetone (10 mL) were refluxed for 6 h after addition of MCPBA. Elution with PhMe/EtOAc 9:1 afforded unreacted **1d** (8 mg, 8%), while elution with PhMe/EtOAc 7:3 and 1:1 afforded compounds **3d** (36 mg, 33%) and **2d** (25 mg, 24%), respectively.

1e → 2e + 3e
17α-benzylestradiol (**1e**, 360 mg, 0.99 mmol), $FeSO_4 \times 7H_2O$ (280 mg, 0.99 mmol) and MCPBA (3×215 mg, 3×0.99 mmol) in acetone (35 mL) were refluxed for 4.5 h after addition of MCPBA. Elution with DCM/acetone 9:1 afforded unreacted **1e** (45 mg, 13%), while elution with DCM/acetone 8:2 and 7:3 afforded compounds **3e** (55 mg, 14%) and **2e** (113 mg, 30%), respectively.

1f → 2f + 3f
17α-butylestradiol (**1f**, 480 mg, 1.46 mmol), $FeSO_4 \times 7H_2O$ (400 mg, 1.46 mmol) and MCPBA (4×315 mg, 4×1.46 mmol) in acetone (65 mL) were refluxed for 4 h after addition of MCPBA. Elution with PhMe/EtOAc 9:1 afforded unreacted **1f** (67 mg, 14%), while elution with PhMe/EtOAc 7:3 and 1:1 afforded compounds **3f** (132 mg, 25%) and **2f** (106 mg, 21%), respectively.

10β-hydroxyestra-1,4-dien-3,17-dione (**2a**): ^{1}H NMR (200 MHz, $CDCl_3$): 7.13 (d, *J*=10.4 Hz, H-C(1)), 6.07 (dd, *J*=10.4 Hz, *J*=2.4 Hz, H-C(2)), 5.92 (t, *J*=2.4 Hz, 1.2 Hz, H-C(4)), 0.97 (s, H_3C-C(13)) ppm. ^{13}C NMR (50 MHz, $CDCl_3$): 220.33, 185.53, 165.09, 150.25, 128.30, 123.09, 70.10, 54.18, 50.10, 47.75, 35.62, 34.58, 32.19, 31.80, 31.03, 22.00, 21.90, 13.73 ppm.

10β,17β-dihydroxy-1,4-estradien-3-one (2b):^{1}H NMR (500 MHz, CD_3OD): 7.22 (d, *J*=10.0 Hz, H-C(1)), 6.12 (dd, *J*=10.0 Hz, *J*=2.0 Hz, H-C(2)), 5.96 (dd(^{irr}t), *J*=2.0; 1.0 Hz, H-C(4)), 3.56 (t, *J*=8.5 Hz, H-C(17)), 2.77 (dddd(^{irr}tdd), *J*=13.5; 12.5; 5.0; 1.0 Hz, $H_β$-C(6)), 2.33 (ddd, *J*=12.5; 4.0; 2.0 Hz, $H_α$-C(6)), 2.01 (dddd(qd), *J*=13.5; 13.5; 13.5; 4.0, $H_β$-C(11)), 2.01-1.88 (m, $H_β$-C(7), overlapped with multiplets from $H_β$-C(8) and $H_α$-C(16)), 2.00-1.96 (m, $H_β$-C(8)), 2.00-1.94 (m, $H_α$-C(16)), 1.87 (ddd, *J*=12.5; 4.0; 2.5 Hz, $H_β$-C(12)), 1.70 (dddd(dtd), *J*=13.5; 4.5; 4.5; 2.5 Hz, $H_α$-C(11)), 1.61 (dddd, *J*= 12.5; 11.0; 7.0; 3.0 Hz, $H_α$-C(15)), 1.48 (dddd, *J*= 13.5; 12.0; 8.5; 3.0 Hz, $H_β$-C(16)), 1.34 (dddd(qd), *J*=12.0; 12.0; 12.0; 6.0 Hz, $H_β$-C(15)), 1.08 (ddd(td), *J*=13.0; 13.0; 4.5 Hz, $H_α$-C(12)), 1.04 (dddd, *J*=13.5; 12.5; 5.0; 1.0 Hz, $H_α$-C(7)), 1.04 (ddd(td), *J*=13.0; 13.0; 5.0 Hz, $H_α$-C(9)), 0.97 (ddd(td), *J*=11.5; 11.5; 7.0 Hz, $H_α$-C(14)), 0.83 (s, H_3C(18)) ppm. ^{13}C NMR (125 MHz, CD_3OD): 187.55 (C(3)), 169.82 (C(5)), 154.41 (C(1)), 127.03 (C(2)), 122.08 (C(4)), 81.53 (C(17)), 70.43 (C(10)), 56.15 (C(9)), 50.45 (C(14)), 43.61 (C(13)), 36.91 (C(12)), 35.66 (C(8)), 34.01 (C(7)), 32.50 (C(6)), 29.87 (C(16)), 23.88 (C(15)), 23.04 (C(11)), 10.84 (C(18)) ppm.

17β-acetoxy-10β-hydroxyestra-1,4-dien-3-one (2c):^{1}H NMR (250 MHz, $CDCl_3$): 7.07 (d, *J*=10.2 Hz, H-C(1)), 6.10 (dd, *J*=10.2 Hz, *J*=2.0 Hz, H-C(2)), 5.95(d, *J*=2.0 Hz, H-C(4)), 4.55 (dd, *J*=9.05; 7.5 Hz, H-C(17)), 2.04 (s, H_3C^{Ac}), 0.89 (s, H_3C(18)) ppm. ^{13}C NMR (62.9 MHz, $CDCl_3$): 185.84, 171.26, 165.90, 150.97, 128.05, 122.89, 82.48, 70.22, 54.28, 49.64, 42.80, 36.44, 34.83, 33.07, 32.07, 27.46, 23.82, 22.30, 21.21, 12.05 ppm.

10β-hydroxy-17β-propionyloxy-1,4-dien-3-one (2d):mp: 189-192 °C (colourless needles, *n*-heptane/DCM). IR(KBr): 3463, 2942, 1734, 1667, 1626, 1191, 1122, 1084, 891, 783 cm^{-1}. λ_{max}^{MeOH}

=239 nm (12000). ^{1}H NMR (500 MHz, $CDCl_3$): 7.08 (d, $J_{1,2}$=10.0 Hz, H-C(1)), 6.10 (dd, $J_{1,2}$=10.0; $J_{2,4}$=2.0 Hz, H-C(2)), 5.92 (dd(^{irr}t), $J_{2,4}$=2.0; 1.0 Hz, H-C(4)), 4.58 (dd, *J*=9.0; 8.0 Hz, $H_α$-C(17)), 2.76 (dddd(tdd), *J*=12.5; 12.5; 4.5; 2.0 Hz), 3.01 (ws, D_2O exch., O-H), 2.34-2.28 (m, $H_α$-C(6)), 2.31 (q, *J*=7.5 Hz, $CH_2^{propionyl}$), 2.16 (dddd(dtd), *J*= 14.0; 9.5; 9.5; 6.5 Hz, $H_α$-C(16)), 2.04-1.92 (m, $H_β$-C(11), $H_β$-C(8) and $H_β$-C(7)), 1.78 (ddd, *J*=12.5; 4.0; 3.0 Hz, $H_β$-C(12)), 1.67 (dddd(dtd), *J*=13.5; 4.0; 4.0; 2.0 Hz, $H_α$-C(11)), 1.65 (dddd, *J*=12.5; 10.5; 7.5; 3.0 Hz, $H_α$-C(15)), 1.50 (dddd, *J*=14.5; 11.5; 8.0; 3.0 Hz, $H_β$-C(16)), 1.39 (dddd(qd), *J*=12.0; 12.0; 12.0; 6.0 Hz, $H_β$-C(16)), 1.17 (ddd(td), *J*=13.0; 13.0; 4.5 Hz, $H_α$-C(12)), 1.13 (t, *J*=7.5 Hz, $CH_3^{propionyl}$), 1.16-1.00 (m, $H_α$-C(7), $H_α$-C(14) and $H_α$-C(9)), 0.89 (s, H_3C(18)) ppm. ^{13}C NMR (125 MHz, $CDCl_3$) : 186.26 (C(3)), 174.82 (C=O ^{propionyl}), 166.73 (C(5)), 151.76 (C(1)), 127.95 (C(2)), 122.87 (C(4)), 82.45 (C(17)), 70.32 (C(10)), 54.58 (C(9)), 49.82 (C(14)), 43.04 (C(13)), 36.66 (C(12)), 34.95 (C(8)), 33.30 (C(7)), 32.26 (C(6)), 28.01 ($CH_2^{propionyl}$), 27.67 (C(16)), 24.00 (C(15)), 22.51 (C(11)), 12.21 (C(18)), 9.46 ($CH_3^{propionyl}$) ppm. Anal. Calcd. for $C_{21}H_{28}O_4$ (344.44): C 73.22 H 8.21, found: C 73.35 H 8.227.

10β,17β-dihydroxy-17α-benzylestra-1,4-dien-3-one (2e): mp = 235-240 ºC (colourless needles, petroleum ether/acetone; decomp.). IR(KBr): 3426, 3180, 2948, 2856, 1661, 1613, 1116, 1023, 890, 818, 756 cm^{-1}. λ_{max}^{MeOH} = 209 (27000), 237 (17000), 280 (7000) nm. ^{1}H NMR (500 MHz, $CDCl_3$+CD_3OD): 7.30-7.20 (m, $5H^{Ph}$), 7.21 (d, $J_{1,2}$=10.5 Hz, H-C(1)), 6.16 (dd, $J_{1,2}$=10.5 Hz, $J_{2,4}$=2.0 Hz, H-C(2)), 6.00 (dd(^{irr}t), $^4J_{2,4}$=2.0 Hz, $^4J_{4,6\beta}$=1.0 Hz, H-C(4)), 2.83 (d, *J*=13.0 Hz, CH_2^{benzyl}), 2.81 (dddd(tdd), *J*= 11.5; 11.5; 5.0; 1.5 Hz, $H_β$-C(6)), 2.56 (d, *J*=13.5 Hz, CH_2^{benzyl}), 2.36 (ddd, *J*=12.0; 3.5; 2.5 Hz, $H_α$-C(6)), 2.11-2.04 (m, $H_β$-C(11)), 2.07 (ddd(td), *J*=12.5; 12.5; 4.0 Hz, $H_β$-C(8)), 2.06-2.00 (m, $H_β$-C(7)), 1.85 (ddd(td), *J*=9.5; 9.5; 5.0 Hz, $H_α$-C(16)), 1.77 (dddd(dtd), *J*= 13.0; 4.0; 4.0; 3.0 Hz, $H_α$-C(11)), 1.68 (ddd, *J*= 12.5; 3.5; 3.0 Hz, $H_β$-C(12)), 1.64-1.58 (m, $H_α$-C(15)), 1.48 (ddd(td), *J*=12.5; 12.5; 4.5 Hz, $H_α$-C(12)), 1.42-1.28 (m, $H_β$-C(16), $H_β$-C(15) and $H_α$-C(14)), 1.14 (ddd(td), *J*=12.5; 12.5; 4.0 Hz, $H_α$-C(9)), 1.10 (dddd(tdd), *J*=12.0; 12.0; 4.0; 3.5 Hz, $H_α$-C(7)), 1.04 (s, H_3C(18)) ppm. ^{13}C NMR (125 MHz, $CDCl_3$): 187.10 (C(3)), 169.09 (C(5)), 153.67 (C(1)), 138.54 (C_{Ar}(1')), 131.00 (C_{Ar}(3')), 127.76 (C_{Ar}(2')), 126.79 (C(2)), 125.99 (C_{Ar}(4')), 121.79 (C(4)), 83.04 (C(17)), 69.96 (C(10)), 55.10 (C(9)), 49.51 (C(14)), 46.98 (C(13)), 42.38 (CH_2^{benzyl}), 35.88 (C(8)), 33.65 (C(7)), 32.17 (C(16)), 32.15 (C(6)), 30.82 (C(12)), 23.69 (C(15)), 22.76 (C(11)), 14.29 (C(18)) ppm. HRMS: calculated for MH^+ 379.22367, measured 379,22677. Anal. Calcd. for $C_{25}H_{30}O_3$ (378.504): C 79.32 H 8.00, found: C 79.25 H 8.099.

17α-butyl-10β,17β-dihydroxyestra-1,4-dien-3-one (2f):mp= 100-103 ºC (colourless needles, petroleum ether/acetone). IR(KBr): 3425, 2949, 2863, 1664, 1624, 1121, 1014, 891, 808, 697 cm^{-1}. λ_{max}^{MeOH} =233 nm (14000). ^{1}H NMR (500 MHz, $CDCl_3$) : 7.21 (d, $J_{1,2}$=10.0 Hz, H-C(1)), 6.12 (dd, $J_{1,2}$=10.0 Hz, $J_{2,4}$=2.0 Hz, H-C(2)), 5.95 (dd(^{irr}t), *J*=2.0; 1.0 Hz, H-C(4)), 2.77 (dddd(tdd), *J*=12.5; 12.5; 5.5; 1.0 Hz, $H_β$-C(6)), 2.32 (ddd, *J*=12.5; 3.5; 2.5 Hz, $H_α$-C(6)), 2.05-1.97 (m, $H_β$-C(8) and $H_β$-C(7)), 2.03 (dddd(tdd), *J*=13.5; 13.5; 3.5; 1.5 Hz, $H_β$-C(11)), 1.90 (ddd, *J*=14.0; 9.5; 6.0 Hz, $H_α$-C(16)), 1.71 (dddd(dtd), *J*=13.0; 4.0; 4.0; 2.5 Hz, $H_α$-C(11)), 1.62-1.50 (m, $H_α$-C(15)), 1.58 (ddd(td), *J*=13.5; 13.5; 2.5 Hz, $H_β$-C(16)), 1.54 (ddd, *J*=12.5; 4.0; 3.0 Hz, $H_β$-C(12)), 1.52-1.42 (m, overlapped H-CH(1')$^{n\text{-}Bu}$ and H-CH(2')$^{n\text{-}Bu}$), 1.41-1.27 (m, overlapped $H_β$-C(15), H-CH(1')$^{n\text{-}Bu}$, H-CH(2')$^{n\text{-}Bu}$ and CH_2(3')$^{n\text{-}Bu}$), 1.21 (ddd(td), *J*=11.5; 11.5; 7.5 Hz, $H_α$-C(14)), 1.06-0.98 (m, overlapped $H_α$-C(9) and $H_α$-C(7)), 0.95 (s, H_3C(18)), 0.92 (t, *J*=7.0 Hz, H_3C(4')$^{n\text{-}Bu}$) ppm. ^{13}C NMR (125 MHz, $CDCl_3$): 187.56 (C(3)), 169.83 (C(5)), 154.43 (C(1)), 126.96 (C(2)), 122.08 (C(4)), 83.41 (C(17)), 70.44 (C(10)), 55.97 (C(9)), 50.16 (C(14)), 47.31 (C(13)), 36.71 (C(1')$^{n\text{-}Bu}$), 36.49 (C(8)), 34.23 (C(7)), 33.31 (C(16)), 32.57 (C(6)), 31.75 (C(12)), 26.31 (C(2')$^{n\text{-}Bu}$), 24.29 (C(15)), 23.95 (C(3')$^{n\text{-}Bu}$), 23.21 (C(11)), 14.37 (C(18)), 13.95 (C(4')$^{n\text{-}Bu}$) ppm. HRMS: calculated for MH^+ 344.23460, measured 344.23270. Anal. Calcd. for $C_{22}H_{32}O_3$ (344.488): C 76.70 H 9.363 found: C 76.55 H 9.423.

10β-hydroxy-4β,5β-epoxyestr-1-en-3,17-dione (3a):^{1}H NMR (250 MHz, DMSO-d_6): 6.68 (d, *J*=10.6 Hz, H-C(1)), 5.79 (s, HO-C(10)), 5.77 (dd, *J*=10.6 Hz, *J*=2.2 Hz, H-C(2)), 3.33 (d, *J*=2.2 Hz, H-C(4)), 2.45-2.25 (m, 2H), 0.81 (s, H_3C-C(13)) ppm. ^{13}C NMR (50 MHz, DMSO-d_6):

219.54, 195.14, 153.06, 122.33, 71.74, 64.59, 59.56, 54.19, 48.99, 46.93, 35.12, 33.62, 30.53, 28.15, 21.45, 20.55, 13.30 ppm.

10β,17β-dihydroxy-4β,5β-epoxyestr-1-en-3-one (3b):colourless oil. IR(KBr): 3515, 3288, 2938, 1681, 1604, 1267, 1130, 1051, 843, 733 cm^{-1}. λ_{max}^{MeOH} =240 nm (7000). ^{1}H NMR (200 MHz, CD_3OD): 6.73 (d, *J*=10.6 Hz, H-C(1)), 5.78 (dd, *J*=10.6 Hz, *J*=2.2 Hz, H-C(2)), 3.56 (t, *J*=8.4 Hz, H-C(17)), 3.32-3.29 (m, 1H), 3.23 (d, *J*=2.2 Hz, H-C(4)), 2.43 (td, *J*=13.4; 4.2 Hz), 2.08-0.88 (m), 0.80 (s, H_3C-C(13)) ppm. ^{13}C NMR (50 MHz, CD_3OD): 197.27, 154.06, 123.69, 82.25, 73.82, 65.95, 61.58, 56.21, 50.97, 43.94, 37.26, 35.93, 30.34, 30.14, 29.77, 24.29, 22,34, 11.44 ppm. Anal. Calcd. for $C_{18}H_{24}O_4$ (304.386): C 71.03 H 7.95, found: C 71.02 H 7.921.

17β-acetoxy-4β,5β-epoxy-10β-hydroxyestr-1-en-3-one (3c):^{1}H NMR (250 MHz, $CDCl_3$): 6.62 (d, *J*=10.7 Hz, H-C(1)), 5.80 (dd, *J*=10.7 Hz, *J*=2.1 Hz, H-C(2)), 4.56 (dd, *J*=7.5 Hz, 1.6 Hz, H-C(17)), 3.28 (d, *J*=2.1 Hz, H-C(4)), 2.03 (s, H_3C^{Ac}), 0.82 (s, $H_3C(18)$) ppm. ^{13}C NMR (62.9 MHz, $CDCl_3$): 194.69, 171.18, 150.87, 123.29, 82.38, 72.81, 64.80, 61.24, 53.57, 49.39, 42.38, 36.06, 34.27, 28.84, 28.66, 27.18, 23.46, 21.09, 21.01, 11.84 ppm.

4β,5β-epoxy-10β-hydroxy-17β-propionyloxyestr-1-en-3-one (3d):colourless oil. IR(KBr): 3450, 2942, 2876, 2853, 1733, 1686, 1279, 1192, 1082, 841, 731 cm^{-1}. λ_{max}^{MeOH} = 230 (9000) nm. ^{1}H NMR (200 MHz, $CDCl_3$): 6.64 (d, $J_{1,2}$=11.0 Hz, H-C(1)), 5.82 (dd, $J_{1,2}$=11.0 Hz, $J_{2,4}$=2.5 Hz, H-C(2)), 4.59 (dd, *J*=9.0; 8.0 Hz, H_α-C(17)), 3.30 (d, $J_{2,4}$=2.5 Hz, H_α-C(4)), 2.82 (ws, H-O, D_2O exch.), 2.46 (ddd(td), *J*=13.5; 13.5; 4.5 Hz), 2.32 (q, *J*=7.5 Hz, $CH_2^{propionyl}$), 2.18 (dddd(dtd), *J*=13.5; 9.5; 9.5; 6.0 Hz, H_α-C(16)), 1.93-1.80 (m, 3H, H_β-C(7), H_β-C(8), H_β-C(11)), 1.77 (ddd, *J*=13.0; 4.5; 2.5 Hz, H_β-C(12)), 1.68 (dddd, *J*=12.0; 10.0; 7.0; 3.0 Hz, H_α-C(15)), 1.56 (dddd(dtd), *J*=13.5; 4.0; 4.0; 2.5 Hz, H_α-C(17)), 1.51 (dddd, *J*=13.5; 11.5; 7.5; 3.0 Hz, H_β-C(16)), 1.38 (dddd(qd), *J*=12.0; 12.0; 12.0; 6.0 Hz, H_β-C(15)), 1.28 (ddd(dt), *J*=13.5; 3.0; 3.0 Hz, H_α-C(6)), 1.18-1.09 (m, 2H, H_α-C(7), H_α-C(12)), 1.13 (t, *J*=7.5 Hz, $CH_3^{propionyl}$), 1.10-1.01 (m, 2H, H_α-C(9), H_α-C(14)), 0.8 (s, $H_3C(18)$) ppm. ^{13}C NMR (50 MHz, $CDCl_3$): 194.65 (C(3)), 174.48 (C=O ^{propionyl}), 150.76 (C(1)), 123.35 (C(2)), 82.14 (C(17)), 72.86 (C(10)), 64.81 (C(5)), 61.31 (C(4)), 53.58 (C(9)), 49.46 (C(14)), 42.48 (C(13)), 36.14 (C(12)), 34.33 (C(8)), 28.87 (C(7)), 28.70 (C(6)), 27.73 ($CH_2^{propionyl}$), 27.26 (C(16)), 23.50 (C(15)), 21.04 (C(11)), 11.86 (C(18)), 9.19 ($CH_3^{propionyl}$) ppm. HRMS: calculated for MH^+ 361.20095, measured 361.19829. Anal. Calcd. for $C_{21}H_{28}O_5$ (360.44): C 69.98 H 7.83 found : C 69.91 H 7.916.

10β,17β-dihidroxy-4β,5β-epoxy-17α-benzylestr-1-en-3-one (3e):colourless oil. IR(KBr): 3520, 3389, 3080, 3030, 2961, 1680, 1630, 1620, 1250, 1123, 893, 709 cm^{-1}. λ_{max}^{MeOH} = 217 (11000), 232 (6000), 279 (2000) nm. ^{1}H NMR (200 MHz, $CDCl_3$): 7.39-7.21 (m, 5H), 6.68 (d, $J_{1,2}$=10.6 Hz, H-C(1)), 5.85 (dd, $J_{1,2}$=10.6 Hz, $J_{2,4}$=2.0 Hz, H-C(2)), 3.32 (d, $J_{2,4}$=2.0 Hz, H_α-C(4)), 2.85 (d, *J*=13.2 Hz, 1H, CH_2^{benzyl}), 2.55 (d, *J*=13.2 Hz, 1H, CH_2^{benzyl}), 2.49 (td, *J*=16.0; 4.0), 2.28 (ws, H-O), 2.13-1.25 (m), 1.02 (s, $H_3C(18)$) ppm. ^{13}C NMR (50 MHz, $CDCl_3$): 194.87 (C(3)), 151.07 (C(1)), 137.99 (C_{Ar}(1')), 130.99 (C_{Ar}(2',6')), 128.16 (C_{Ar}(3',5')), 126.40 (C_{Ar}(4')), 123.34 (C(2)), 82.95 (C(17)), 72.98 (C(10)), 64.95 (C(5)), 61.34 (C(4)), 53.73 (C(9)), 49.18 (C(14)), 46.34 (C(13)), 42.21 (CH_2^{benzyl}), 35.43 (C(8)), 33.30 (C(16)), 30.63 (C(7)), 29.08 (C(6)), 28.75 (C(11)), 23.82 (C(12)), 21.30 (C(15)), 14.31 (C(18)) ppm. HRMS: calculated for MH^+ 395.21778, measured 395.22169. Anal. Calcd. for $C_{25}H_{30}O_4$ (394.55): C 76.10 H 7.68, found: C 76.18 H 7.754.

17α-butyl-10β,17β-dihydroxy-4β,5β-epoxyestr-1-en-3-one (3f):colourless oil. IR(KBr): 3477, 3425, 2937, 2874, 1683, 1620, 1293, 1133, 1082, 842, 735, 701 cm^{-1}. λ_{max}^{MeOH} = 231 (6000), 280 (800) nm. ^{1}H NMR (200 MHz, $CDCl_3$): 6.65 (d, $J_{1,2}$=10.5 Hz, H-C(1)), 5.82 (dd, $J_{1,2}$=10.5 Hz, $J_{2,4}$=2.0 Hz, H-C(2)), 3.29 (d, $J_{2,4}$=2.0 Hz, H_α-C(4)), 2.46 (ddd(td), *J*=13.5; 13.5; 4.0 Hz, H_β-C(6)), 1.97 (ddd, *J*=15.0; 10.5; 7.5 Hz, H_α-C(16)), 1.91 (dddd(qd), *J*=13.0; 13.0; 13.0; 4.0 Hz, H_β-C(11)), 1.91-1.82 (m, 2H, H_β-C(7), H_β-C(8)), 1.62-1.53 (m, 3H, H_α-C(11), H_α-C(15), H_β-C(16)), 1.50 (ddd(dt), *J*=12.5; 3.5; 3.5 Hz, H_β-C(12)), 1.47-1.30 (m, 8H, H_α-C(12), H_β-C(15), $H_2C(1')^{n\text{-}Bu}$, $H_2C(2')^{n\text{-}Bu}$, $H_2C(3')^{n\text{-}Bu}$), 1.27 (ddd(dt), *J*=13.5; 3.0; 3.0 Hz, H_α-C(6)), 1.19 (ddd(td), *J*=12.0; 12.0; 7.5 Hz, H_α-C(14)), 1.07 (dddd(qd), *J*=13.0; 13.0; 13.0; 3.5 Hz, H_α-C(7)), 1.02 (ddd, *J*=13.0; 12.5;

4.0 Hz, H$_\alpha$-C(9)), 0.94 (s, H$_3$C(18)), 0.91 (t, *J*=7.0 Hz, H$_3$C(4')$^{n\text{-Bu}}$) ppm. ^{13}C NMR (50 MHz, CDCl$_3$): 194.80 (C(3)), 151.14 (C(1)), 123.20 (C(2)), 83.32 (C(17)), 72.93 (C(10)), 64.93 (C(5)), 61.29 (C(4)), 53.77 (C(9)), 49.15 (C(14)), 46.19 (C(13)), 36.33 (C(1')$^{n\text{-Bu}}$), 35.38(C(8)), 33.97(C(16)), 30.83 (C(12)), 29.10 (C(7)), 28.76 (C(6)), 25.69 (C(2')$^{n\text{-Bu}}$), 23.63 (C(15)), 23.48 (C(3')$^{n\text{-Bu}}$), 21.32 (C(11)), 14.23 (C(18)), 14.16 (C(4')$^{n\text{-Bu}}$) ppm. HRMS: calculated for MH$^+$ 345.24702, measured 345.24242. Anal. Calcd. for $C_{22}H_{32}O_4$ (360.487): C 73.28 H 8.96, found: C 73.35 H 9.06.

General procedure 2: Oxidation of steroidal epoxyquinols with H_2O_2/ Na_2CO_3 system (**3c**,**d** → **4c**,**d**)

To a solution of epoxyquinol (1 mmol) in EtOH (5 mL) at 60 °C, oxidizing solution (1 mL) was added (oxidizing solution was made from 200 mg of Na_2CO_3, 1 mL of H_2O_2 (30%) and 5 mL of water). After 20 minutes of reflux, reaction mixture was cooled, diluted with water (5 mL), neutralized with diluted HCl (3.6 %, 1 mL) and evaporated to the one half of the volume. Solution was extracted with DCM (4×5 mL) and combined organic layers were dried over anh. Na_2SO_4, filtered and evaporated to dryness. Crude products were purified by dry-flash chromatography on silica.

17β-acetoxy-1β,2β;4β,5β-diepoxy-10β-hydroxyestra-3-one(4c) was synthesized from 17β-acetoxy-4β,5β-epoxy-10β-hydroxyestra-1-en-3-one (**3c**, 340 mg, 0.980 mmol), according to *General procedure 2*, as a colourless needles crystalized from benzene (270 mg, 76%). mp = 222-225 °C (colourless needles, benzene). IR (KBr): 3446, 2950, 1734, 1712, 1250, 1045, 824, 765, 735 cm^{-1}. ^{1}H NMR (500 MHz, CDCl$_3$): 4.60 (dd, *J*=9.0; 8.0 Hz, H$_\alpha$-C(17)), 3.84 (d, $J_{1,2}$=4.0 Hz, H$_\alpha$-C(1)), 3.49 (dd, *J*=4.0; 2.5 Hz, H$_\alpha$-C(2)), 3.26 (d, $J_{2,4}$=2.5 Hz, H$_\alpha$-C(4)), 3.22 (s, O-H), 2.43 (ddd(td), *J*=13.0; 13.0; 4.0 Hz, H$_\beta$-C(6)), 2.18 (dddd(dtd), *J*=14.5; 9.5; 9.5; 6.5 Hz, H$_\alpha$-C(16)), 2.05 (s, H$_3$C-C(O)), 1.91 (dddd(qd), *J*=12.0; 12.0; 12.0; 4.0 Hz, H$_\beta$-C(11)), 1.86-1.78 (m, 3H (7β, 8β, 12β)), 1.75 (dddd(dtd), *J*=13.0; 4.0; 4.0; 2.5 Hz, H$_\alpha$-C(11)), 1.66 (dddd, *J*=12.5; 10.0; 7.5; 3.0 Hz, H$_\alpha$-C(15)), 1.53 (dddd, *J*=14.5; 12.0; 8.0; 3.5 Hz, H$_\beta$-C(16)), 1.37 (dddd(qd), *J*=12.0; 12.0; 12.0; 6.0 Hz, H$_\beta$-C(15)), 1.21 (ddd(td), *J*= 13.0; 13.0; 4.5 Hz, H$_\alpha$-C(12)), 1.15 (ddd, *J*=12.5; 10.5; 4.5 Hz, H$_\alpha$-C(9)), 1.10-1.00 (m, 3H (6α, 7α, 14α)), 0.88 (s, H$_3$C(18)) ppm. ^{13}C NMR (125 MHz, CDCl$_3$): 199.93 (C(3)), 171.43 (C=O^{Ac}), 82.63 (C(17)), 71.89 (C(5)), 70.25 (C(10)), 63.60 (C(4)), 62.94 (C(1)), 57.35 (C(2)), 49.77 (C(14)), 49.04 (C(9)), 42.55 (C(13)), 36.44 (C(12)), 34.19 (C(8)), 30.72 (C(6)), 29.10 (C(7)), 27.55 (C(16)), 23.80 (C(15)), 21.35 (CH$_3^{Ac}$), 20.88 (C(11)), 12.12 (C(18)) ppm. MS (DCI, m/e): 363 (MH$^+$, 100), 345 (MH$^+$-H$_2$O, 24), 303 (MH$^+$-AcOH, 70). Anal. Calcd. for $C_{20}H_{26}O_6$ (362.417): C 66.28 H 7.231, found: C 66.31 H 7.253.

1β,2β,4β,5β-diepoxy-10β-hydroxy-17β-propionyloxyestra-3-one (4d) was synthesized from 4β,5β-epoxy-10β-hydroxy-17β-propionyloxyestra-1-en-3-one (**3d**, 80 mg, 0.221 mmol) according to *General procedure 2*, as a colourless needles crystalized from benzene (31 mg, 37%). mp = 177-181 °C (colourless needles, benzene). IR(KBr): 3459, 2928, 1732, 1191, 1078, 826, 765, 720 cm^{-1}. ^{1}H NMR (200 MHz, CDCl$_3$): 4.62 (dd, *J*=8.8 Hz, *J*=7.6 Hz, H$_\alpha$-C(17)), 3.84 (d, $J_{1,2}$=4.0 Hz, H$_\alpha$-C(1)), 3.49 (dd, $J_{1,2}$= 4.0 Hz, $J_{2,4}$= 2.6 Hz, H$_\alpha$-C(2)), 3.27 (d, $J_{2,4}$= 2.6 Hz, H$_\alpha$-C(4)), 2.74 (s, HO-C(10)), 2.41 (td, *J*=13.8; 4.4 Hz), 2.33 (q, *J*=7.6 Hz, CH$_2^{propionyl}$), 2.28-2.08 (m), 2.05-0.95 (m), 1.14 (t, *J*=7.6 Hz, H$_3$C^{propionyl}), 0.88 (s, H$_3$C(18)) ppm. ^{13}C NMR (50 MHz, CDCl$_3$): 199.58 (C(3)), 174.65 (C(O)Et), 82.12 (C(17)), 71.59 (C(10)), 70.10 (C(5)), 63.51 (C(1)), 62.60 (C(2)), 57.19 (C(4)), 49.51 (C(14)), 48.65 (C(9)), 42.34 (C(13)), 36.18 (C(16)), 33.98 (C(8)), 30.48 (C(6)), 28.79 (C(7)), 27.73 (C(O)-**C**H$_2$-CH$_3$), 27.31 (C(11)), 23.55 (C(12)), 20.61 (C(15)), 11.86 (**C**H$_3$-C(13)), 9.20 (H$_3$**C**-CH$_2$-C(O)) ppm. MS (DCI, m/e): 377 (MH$^+$, 100), 359 (MH$^+$-H$_2$O, 12), 303 (MH$^+$-C$_2$H$_5$COOH, 40). Anal. Calcd. for $C_{21}H_{28}O_6$ (376.443): C 67.00 H 7.498, found: C 67.18 H 7.522.

Epoxy-ring opening reaction with LiCl/Amberlyst 15 in acetone (**3a** → **5** + **6**)

A) Suspension of epoxyquinol **3a** (200 mg, 0.661 mmol), LiCl (90 mg, 2.123 mmol) and Amberlyst 15 (450 mg) in acetone (5 mL) was stirred at room temperature for 30 minutes. After filtration and evaporation, mixture was purified by dry-flash chromatography over SiO_2 (eluents

DCM/acetone 9:1→1:1). Epoxyquinol **3a** (61mg, 31%) and 4α-chloro-5β,10β-dihydroxyestra-1-en-3,17-dione (**6**, 105 mg, 47%, colourless plates) were isolated.

B) Suspension of epoxyquinol **3a** (200 mg, 0.661 mmol), LiCl (90 mg, 2.123 mmol) and Amberlyst 15 (450 mg) in acetone (5 mL) was stirred at room temperature for 48 hours. After filtration and evaporation, mixture was purified by dry-flash chromatography over SiO_2 (eluents DCM/acetone 9:1→1:1). Epoxyquinol **3a** (39 mg, 20%), 4-chloro-10β-hydroxyestra-1,4-dien-17-one (**5**, 80 mg, 42%, colourless plates) and 4α-chloro-5β,10β-dihydroxyestra-1-en-3,17-dione (**6**, 18 mg, 8%, colourless plates) were isolated and products **5** and **6** were crystalized from DIPE and methanol, respectively.

4-chloro-10β-hydroxyestra-1,4-dien-17-one (5): mp = 243-245 °C (colourless plates, DIPE). IR(KBr): 3433, 2940, 2901, 1736, 1659, 1635, 1595, 1091, 1000, 899, 837, 794 cm^{-1}. λ_{max}^{MeOH} = 242 (8000), 282 (4000). ^{1}H NMR (200 MHz, $CDCl_3$): 7.24 (d, $J_{1,2}$=10.5 Hz, H-C(1)), 6.26 (d, $J_{1,2}$=10.0 Hz, H-C(2)), 3.16 (ddd(dt), *J*=13.0; 3.5; 3.5 Hz, $H_α$-C(6)), 2.69 (ddd(^{irr}td), *J*=13.5; 13.0; 5.0 Hz, $H_β$-C(6)), 2.45 (ddd, *J*=19.0; 8.0; 1.0 Hz; $H_β$-C(16)), 2.17 (dddd(qd), *J*=11.0; 11.0; 11.0; 4.0 Hz, $H_β$-C(8)), 2.12-2.07 (m, $H_β$-C(7)), 2.08 (ddd(dt), *J*=19.0; 9.0; 9.0 Hz, $H_α$-C(16)), 2.02 (ddd, *J*=13.5; 12.5; 4.0 Hz, $H_β$-C(11)), 1.98 (dddd, *J*=12.0; 9.0; 6.0; 1.0 Hz, $H_α$-C(15)), 1.82-1.76 (m, 2H, $H_α$-C(11), $H_β$-C(12)), 1.65 (dddd(tt), *J*=12.5; 12.5; 9.0; 9.0 Hz, $H_β$-C(15)), 1.33 (ddd, *J*=12.5; 11.5; 5.5 Hz, $H_α$-C(14)), 1.26 (ddd(td), *J*=13.5; 13.5; 4.5 Hz, $H_α$-C(12)), 1.17 (ddd(dt), *J*=11.5; 4.0; 4.0 Hz, $H_α$-C(9)), 1.14 (dddd, *J*=13.5; 12.5; 10.5; 4.0 Hz, $H_α$-C(7)), 0.94 (s, H_3C(18)) ppm. ^{13}C NMR (50 MHz, $CDCl_3$): 223.29(C(17)), 179.92(C(3)), 163.51 (C(5)), 154.52 (C(1)), 127.46 (C(4)), 126.92(C(2)), 73.31 (C(10)), 57.14 (C(9)), 51.22 (C(14)), 49.23 (C(13)), 36.66 (C(16)), 35.96 (C(8)), 32.53 (C(7)), 32.40 (C(12)), 29.70 (C(6)), 22.70 (C(11)), 23.08 (C(15)), 14.26 (C(18)) ppm. Anal. Calcd. for $C_{18}H_{21}ClO_3$ (320.811): C 59.19 H 5.795, found: C 59.35 H 5.815.

4α-chloro-5β,10β-dihydroxyestra-1-en-3,17-dione (6): mp = 216-219 °C (colourless plates, MeOH). IR (KBr): 3541, 3361, 1737, 1678, 1277, 860, 835, 795 cm^{-1}. λ_{max}^{MeOH} = 225 (13000). ^{1}H NMR (250 MHz, aceton-d_6): 6.89 (d, $J_{1,2}$=10.6 Hz, H-C(1)), 5.92 (dd, $J_{1,2}$=10.6 Hz, $J_{2,4}$=1.4 Hz, H-C(2)), 5.24 (s, HO-), 4.22 (s, HO-), 4.21 (d, $J_{2,4}$=1.4 Hz, $H_β$-C(4)), 2.50-2.30 (m), 1.85-1.18 (m), 0.91 (s, H_3C-C(13)) ppm. ^{13}C NMR-DEPT (50 MHz, aceton-d_6) : 219.78 (C(17)), 206.57 (C(3)), 153.24 (C(1)), 124.56 (C(2)), 75.46 (C(5)), 73.17 (C(10)), 62.41 (C(4)), 50.99 (C(14)), 48.59 (C(9)), 47.99 (C(13)), 35.84 (C(7)), 35.70 (C(16)), 33.84 (C(8)), 32.11 (C(11)), 27.27 (C(6)), 22.39 (C(12)), 21.70 (C(15)), 13.87 (**C**H_3-C(13)) ppm. Anal. Calcd. for $C_{18}H_{23}ClO_4$ (338.826): C 63.81 H 6.84, found: C 63.75 H 6.917.

---

[1]D. Milić, T. Kop. Z. Juranić, M. J. Gašić, B. Tinant, G. Pocsfalvi and B. A. Šolaja, *Steroids*, 2005, **70**, 922-932.

[2] L.C. Ciobanu, R.P.Boivin, V. Luu-The, F.Labrie and D. Poirier, *J. Med. Chem.* 1999, **42**, 2280-2286.

[3] R.P.Boivin, V. Luu-The, R. Lachance, F.Labrie and D. Poirier, *J. Med. Chem.* 2000, **43**, 4465-4478.

[4]D. R. Milić, M. J. Gašić, W. Muster, J. J. Csanadi and B. A. Šolaja, *Tetrahedron*, 1997, **53**, 14073-14084.

[5]L. Prokai, V. Nguyen, S. Szarka, P. Garg,G. Sabnis,H. A. Bimonte-Nelson, K. J. McLaughlin, J. S. Talboom,C. D. Conrad, P. J. Shughrue, T. D. Gould, A. Brodie,I. Merchenthaler, P. Koulen, K. Prokai-Tatrai, *Sci. Transl. Med.*, 2015, **7**, 297ra113.

2. Table S1: Antineoplastic activity of newly synthesized derivatives

Compound	IC50 ±SD (µM)[a]		
	Fem-X[b]	HeLa[c]	K562[d]
**2b**	>100	90.71±6.80	92.56±5.29
**2d**	62	24	34.5
**2e**	70.61±7.97	49.63±15.81	36.13±6.31
**2f**	41.73±2.67	35.05±9.32	26.74±5.66
**5**	81.38±1.21	47.69±1.37	42.62±10.35
**3b**	8.18±0.69	8.36±2.74	5.67±0.33
**3d**	1.16±0.37	1.48±0.16	1.30±0.98
**3e**	5.05±1.71	5.50±2.85	3.72±2.16
**3f**	4.65±1.55	3.95±1.43	1.00±0.37
**4c**	0.51±0.02	1.10±0.06	0.66±0.05
**4d**	0.55±0.03	0.87±0.38	0.84±0.07
**6**	3.22±0.96	7.04±2.96	3.88±0.45

[a] concentrations that induce 50% decrease in cell survival; [b] human malignant melanoma; [c] human cervix carcinoma; [d] human myelogenous leukemia.

3. NMR spectra of compounds

3.1. Spectra of compound **2b** 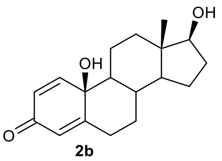

2b

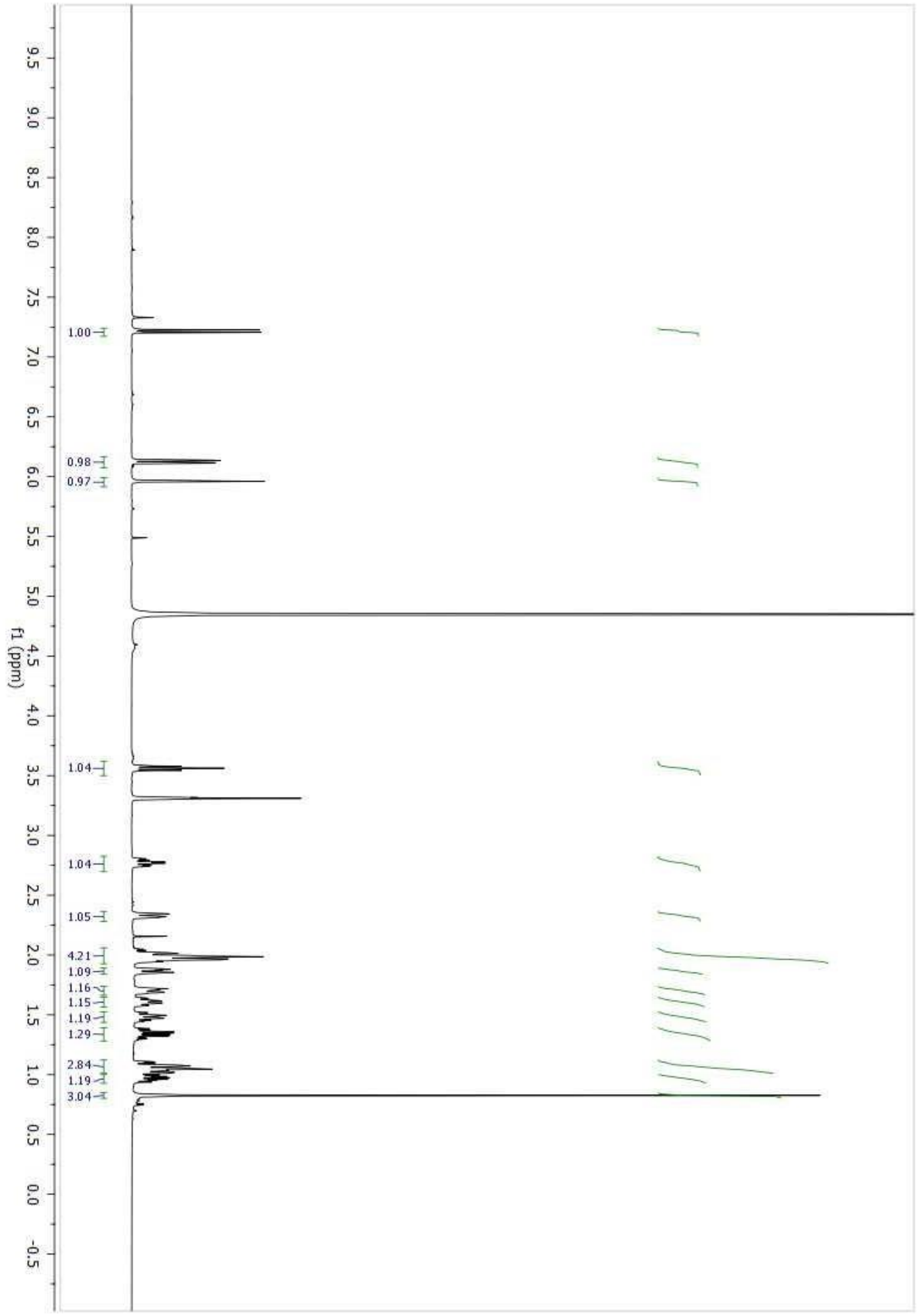

^{1}H NMR spectrum (500 MHz, Methanol-d_4) of compound **2b**

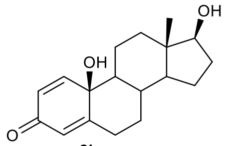

2b

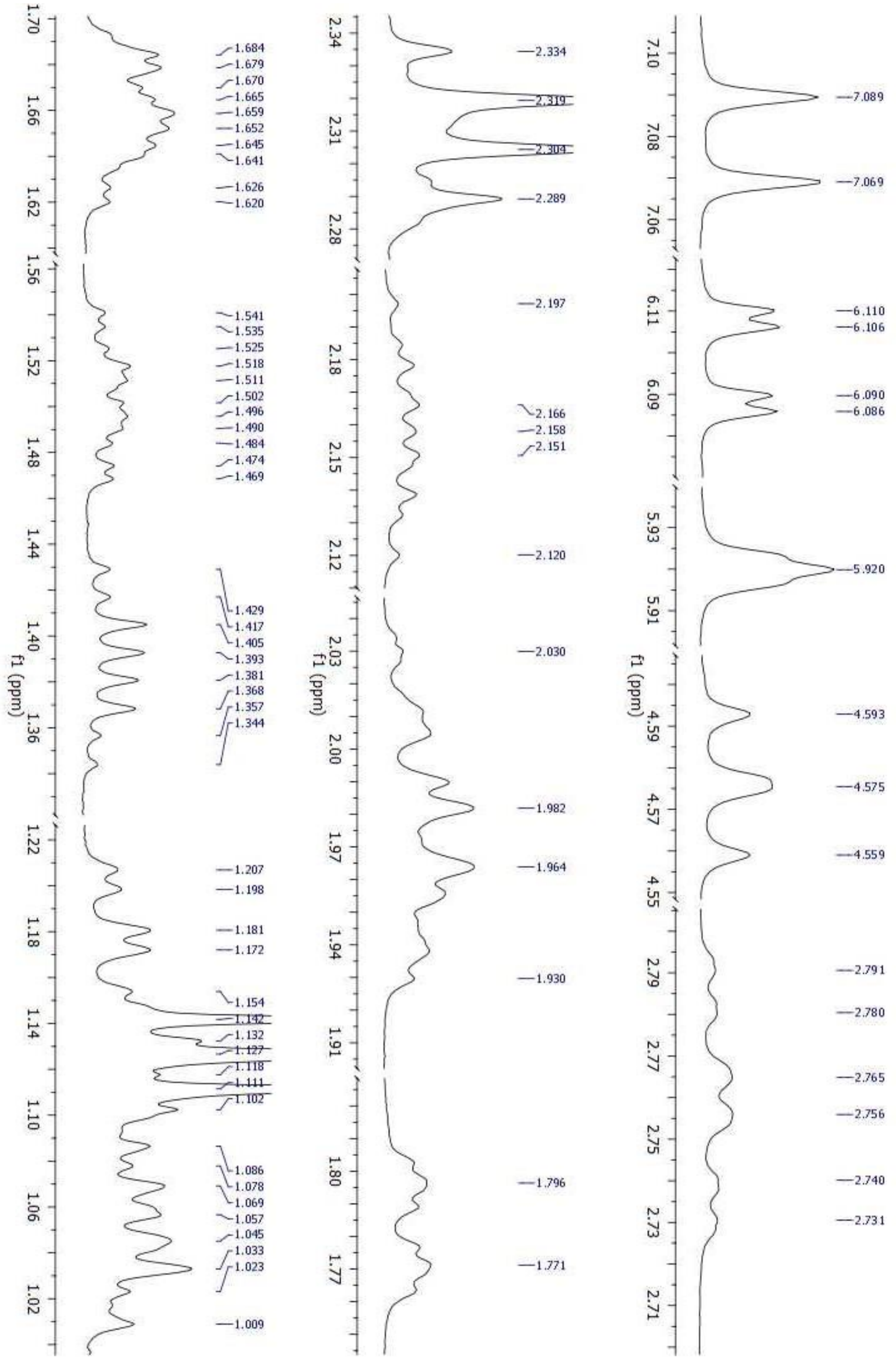

^{1}H NMR spectrum (500 MHz, Methanol-d_4) of compound **2b**

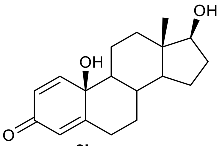

2b

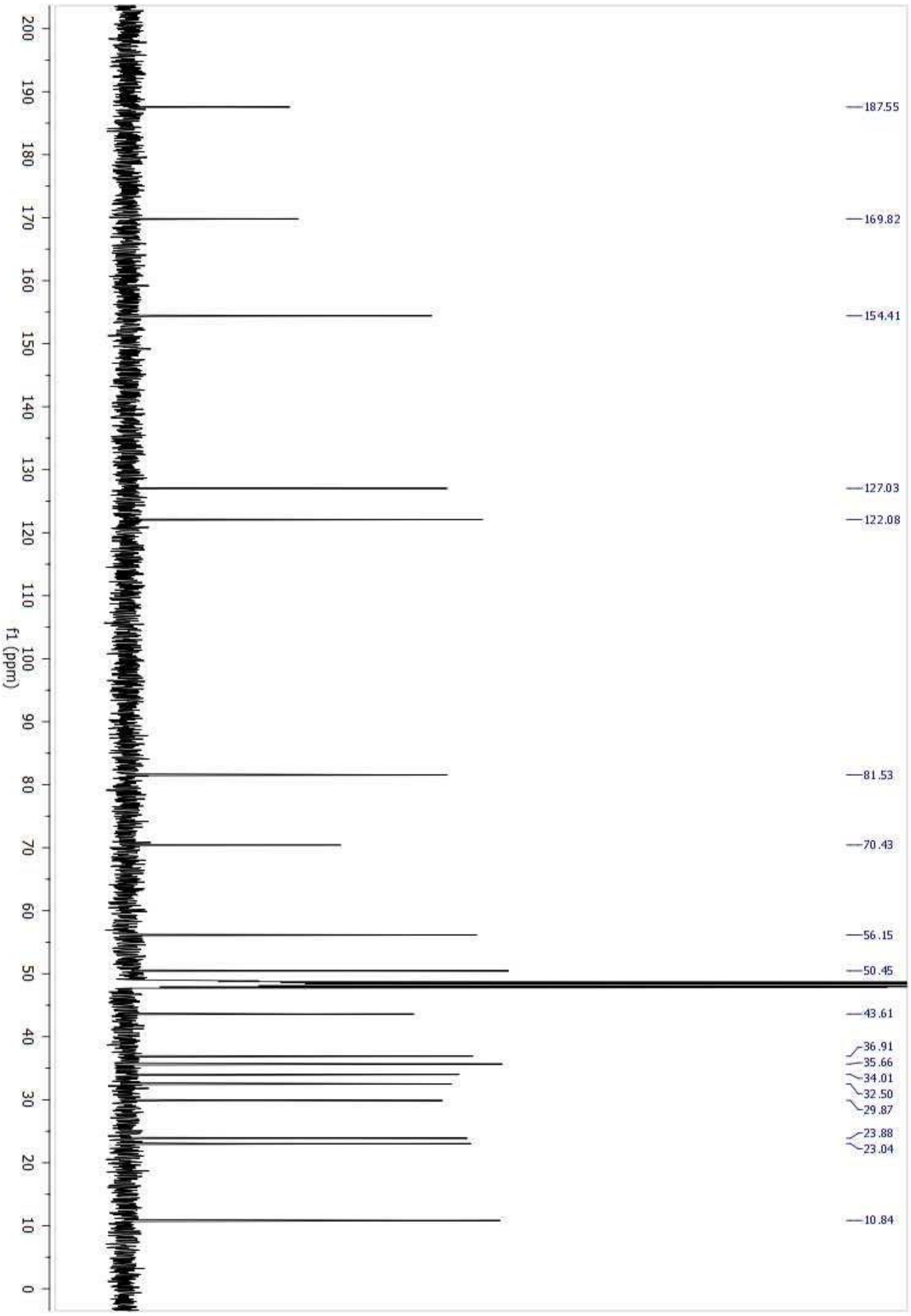

^{13}C NMR spectrum (125 MHz, Methanol-d_4) of compound **2b**

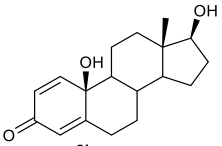

2b

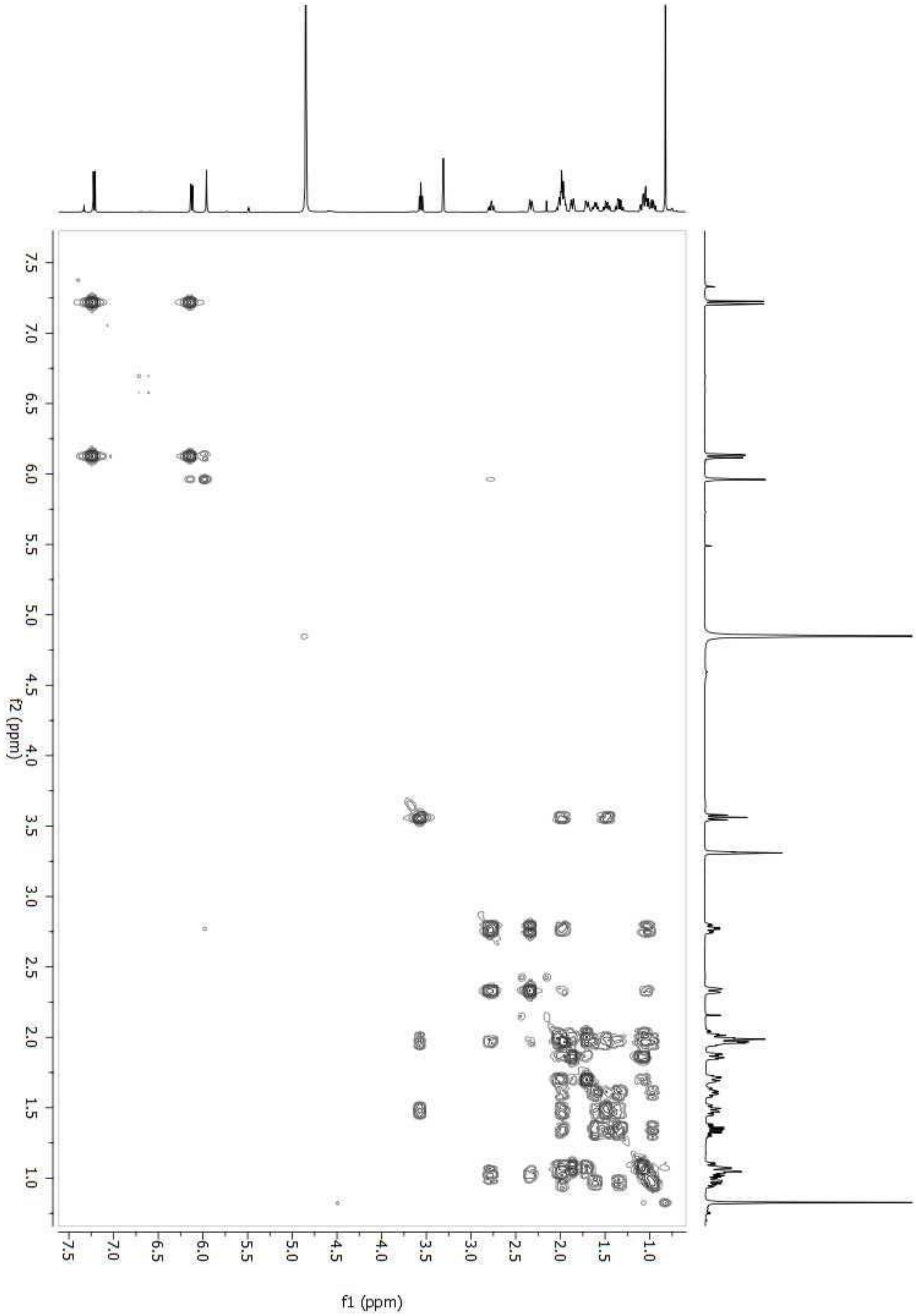

COSY spectrum of compound **2b**(Methanol-*d*$_4$)

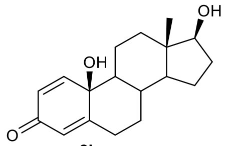

2b

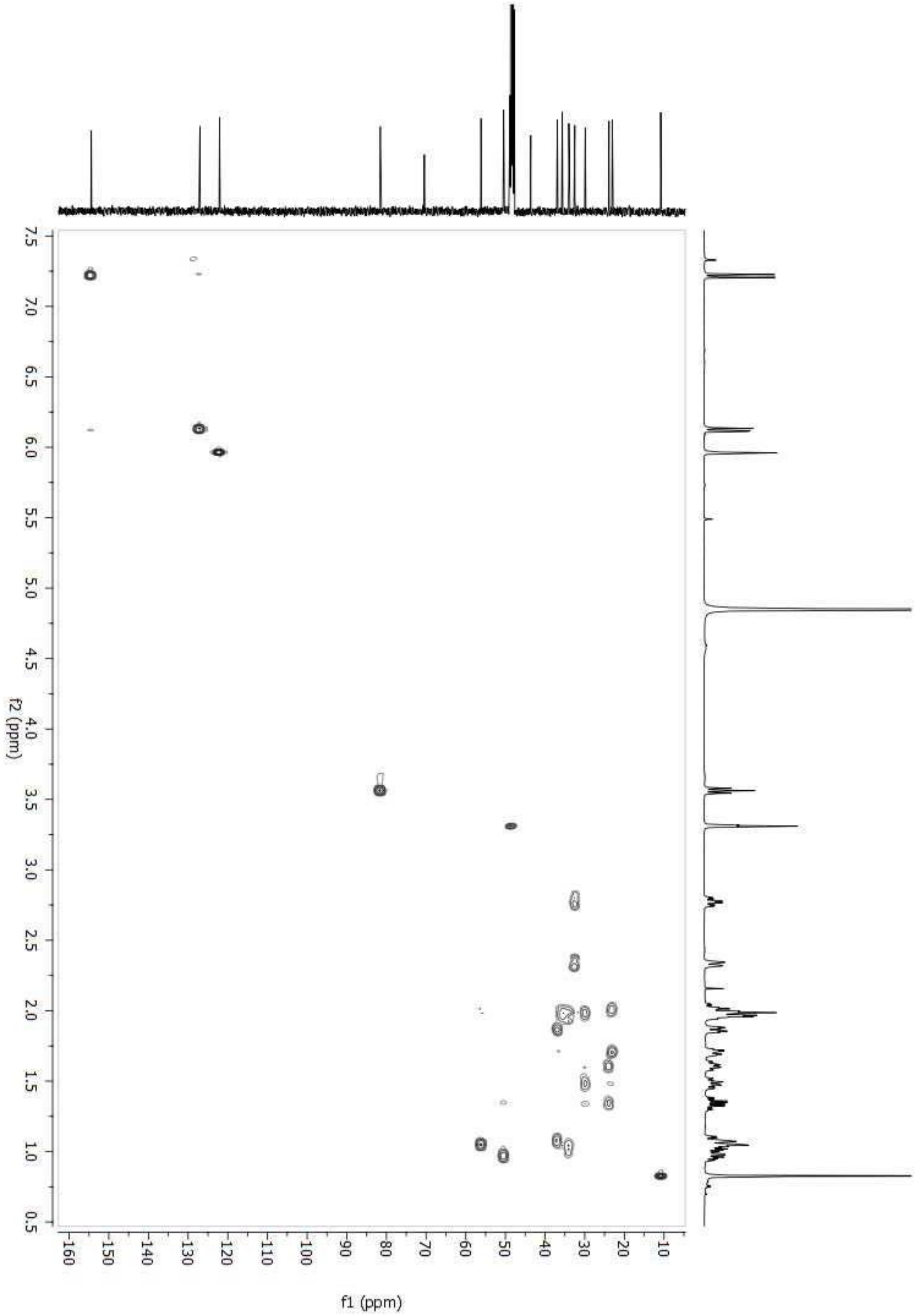

HSQC spectrum of compound **2b**(Methanol-d_4)

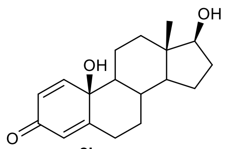

2b

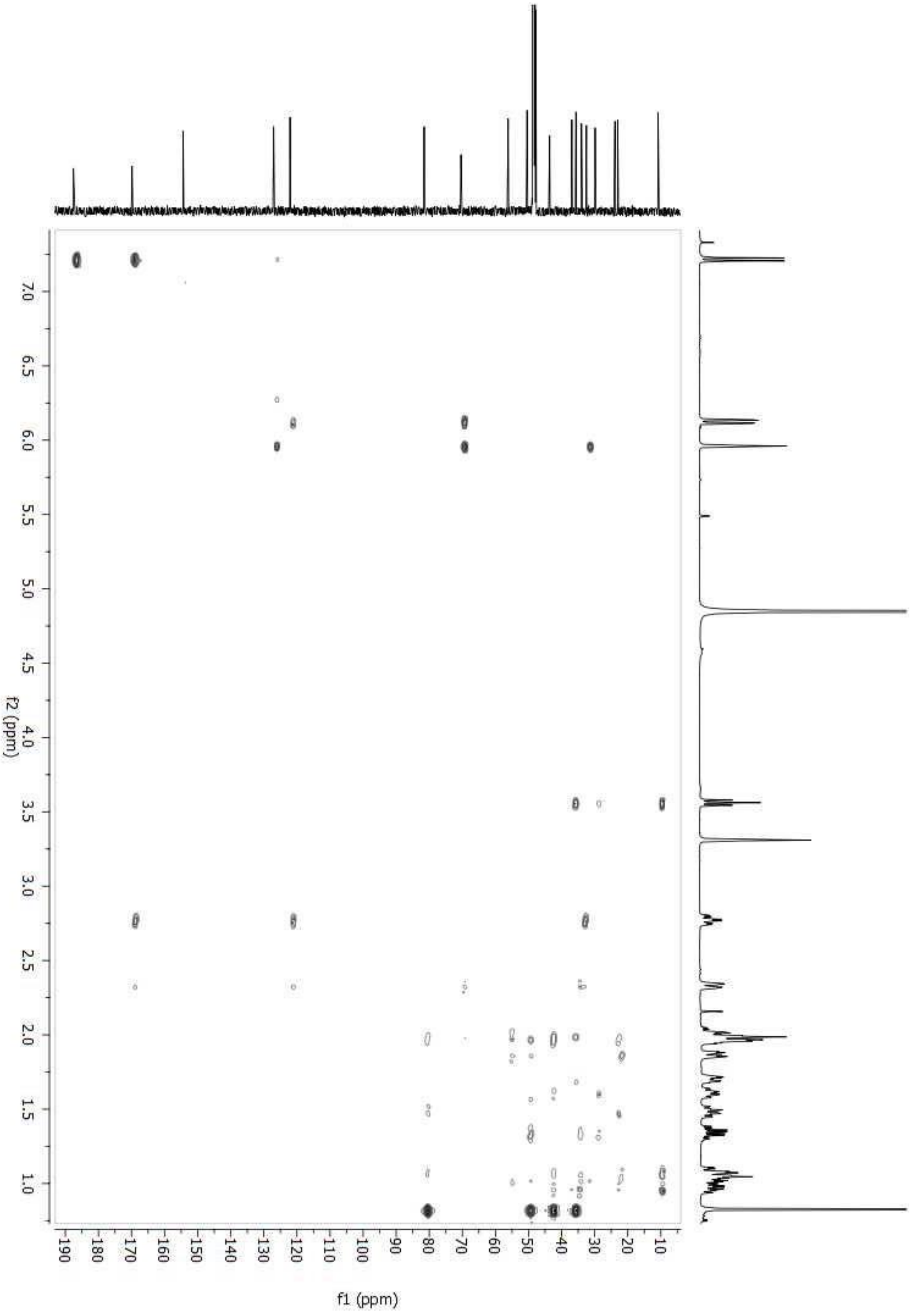

HMBC spectrum of compound **2b**(Methanol-d_4)

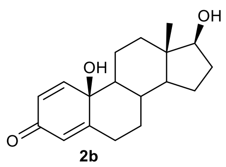

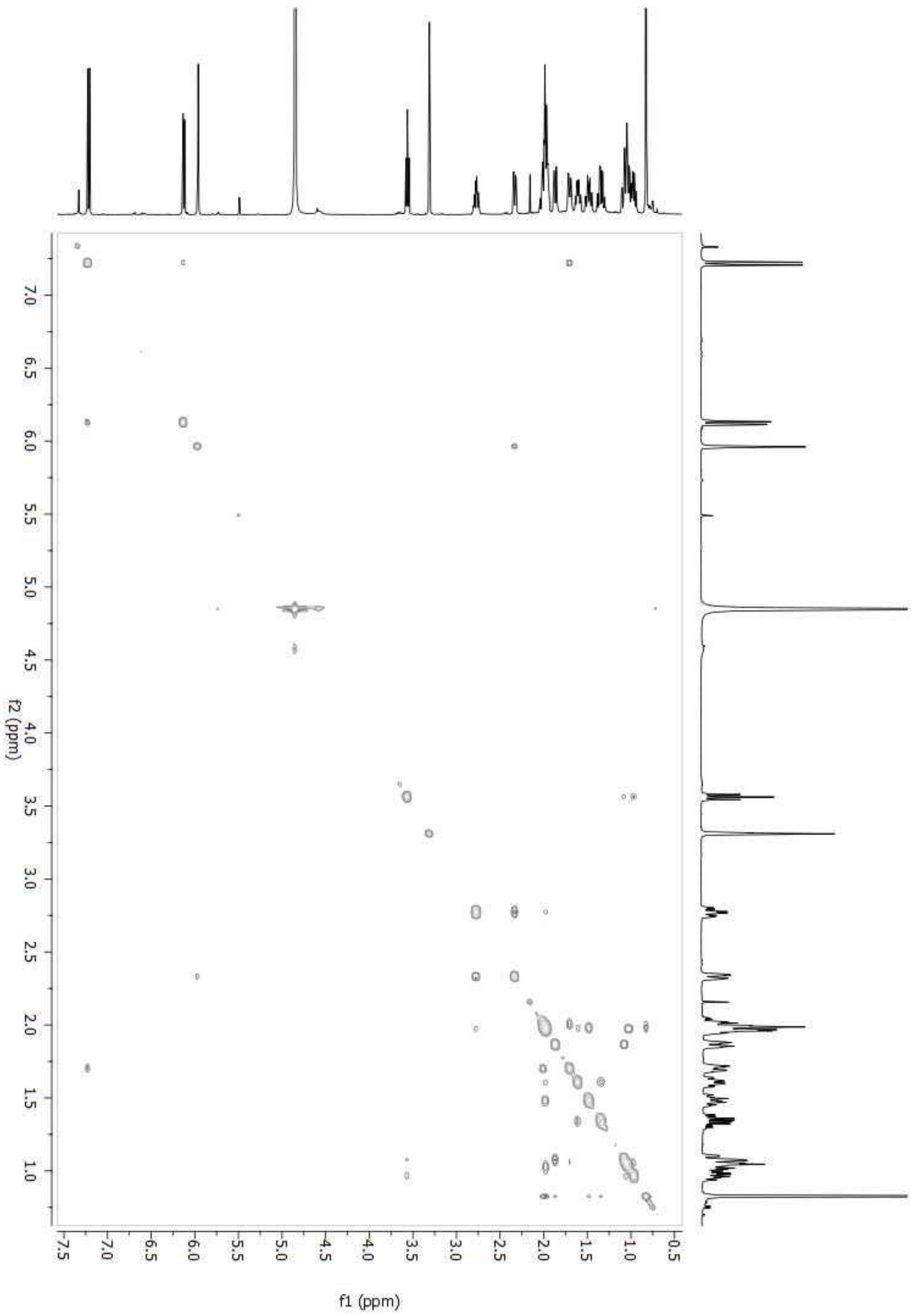

NOESY spectrum o f compound **2b**(Methanol-d_4)

3.2. Spectra of compound **2d** 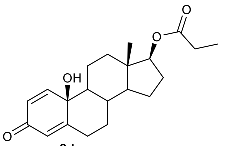

2d

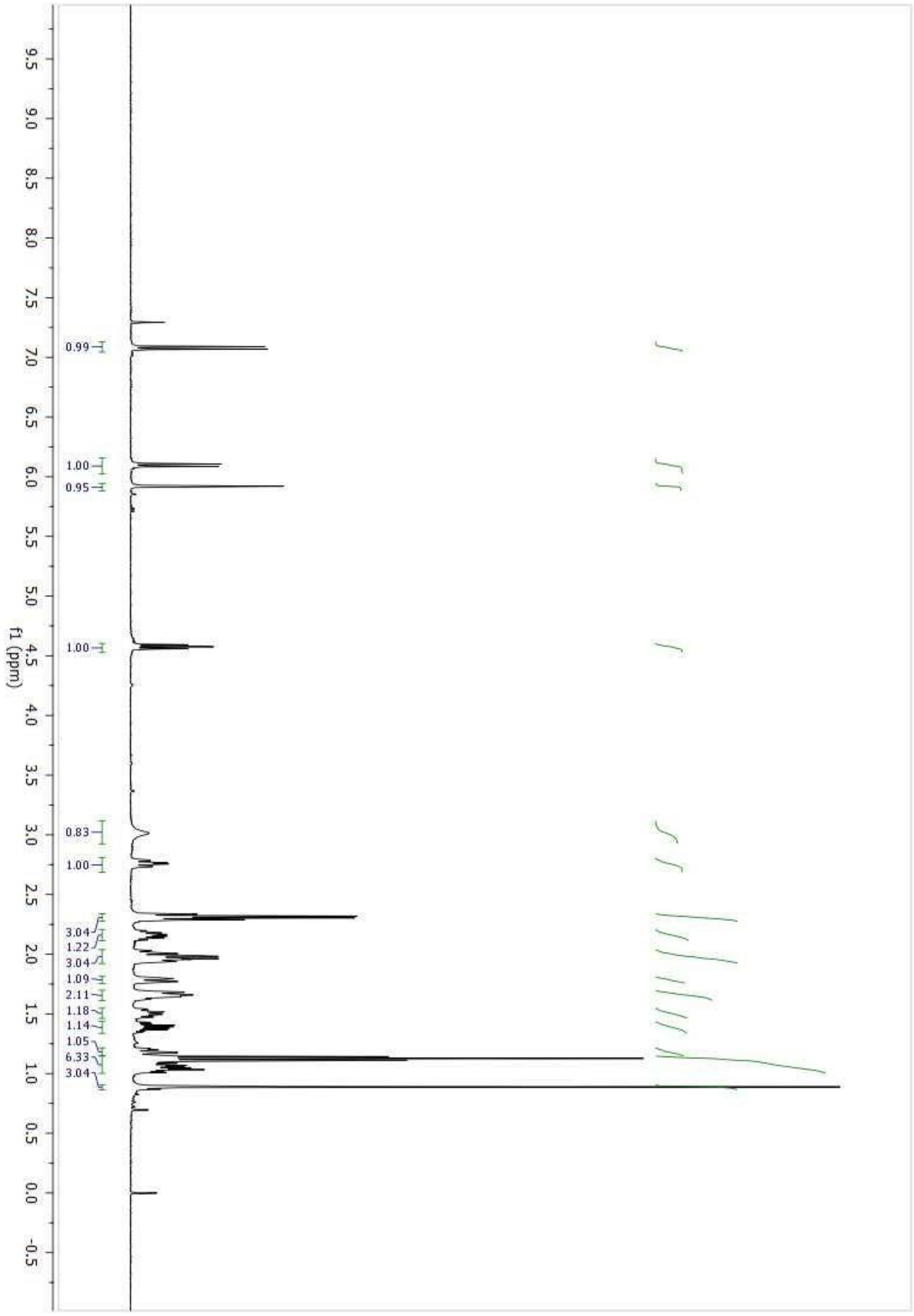

^{1}H NMR spectrum of compound **2d**(500 MHz, $CDCl_3$)

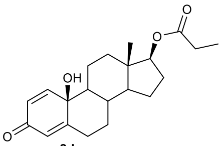

2d

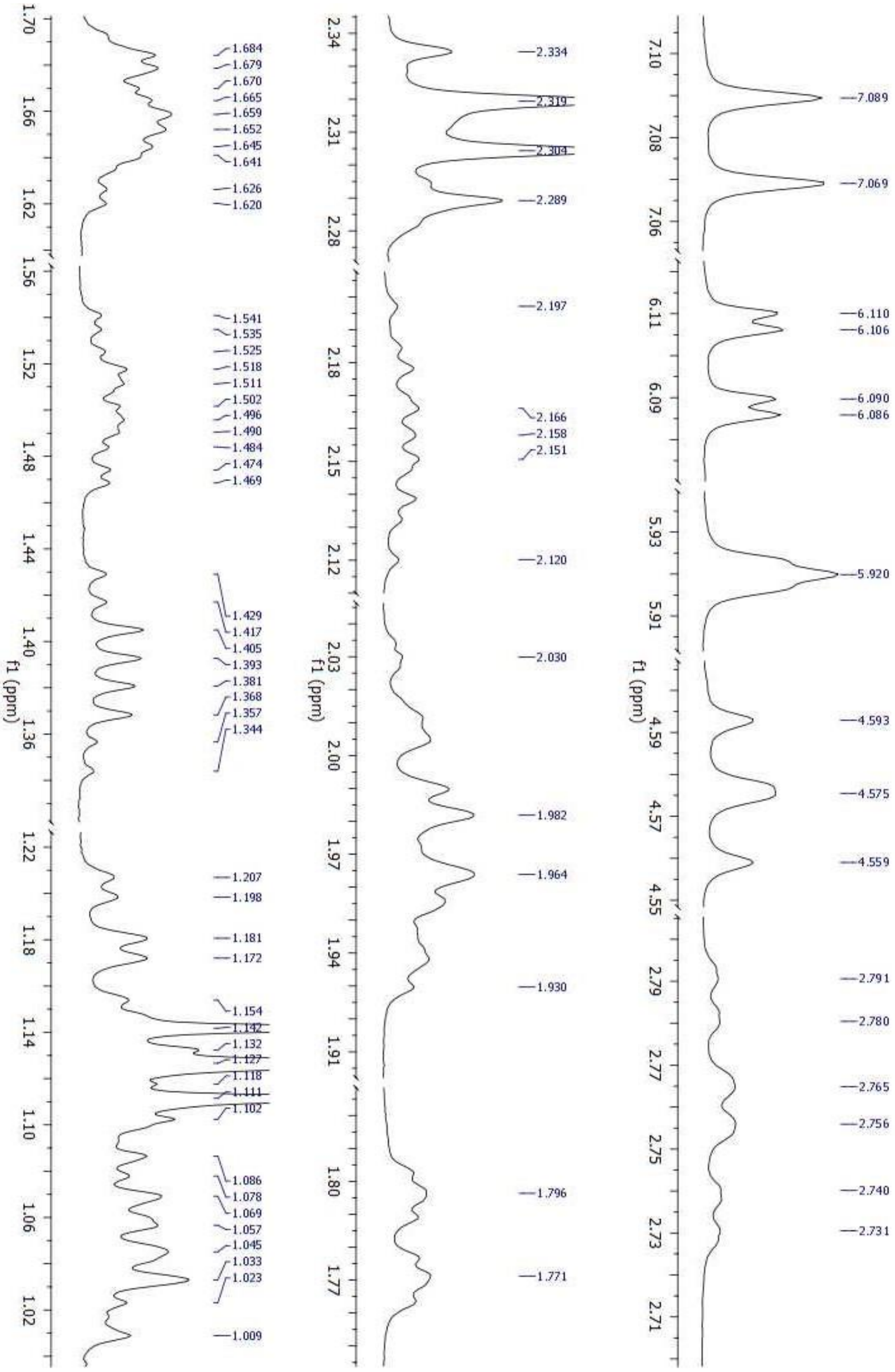

^{1}H NMR spectrum of compound **2d** (500 MHz, $CDCl_3$)

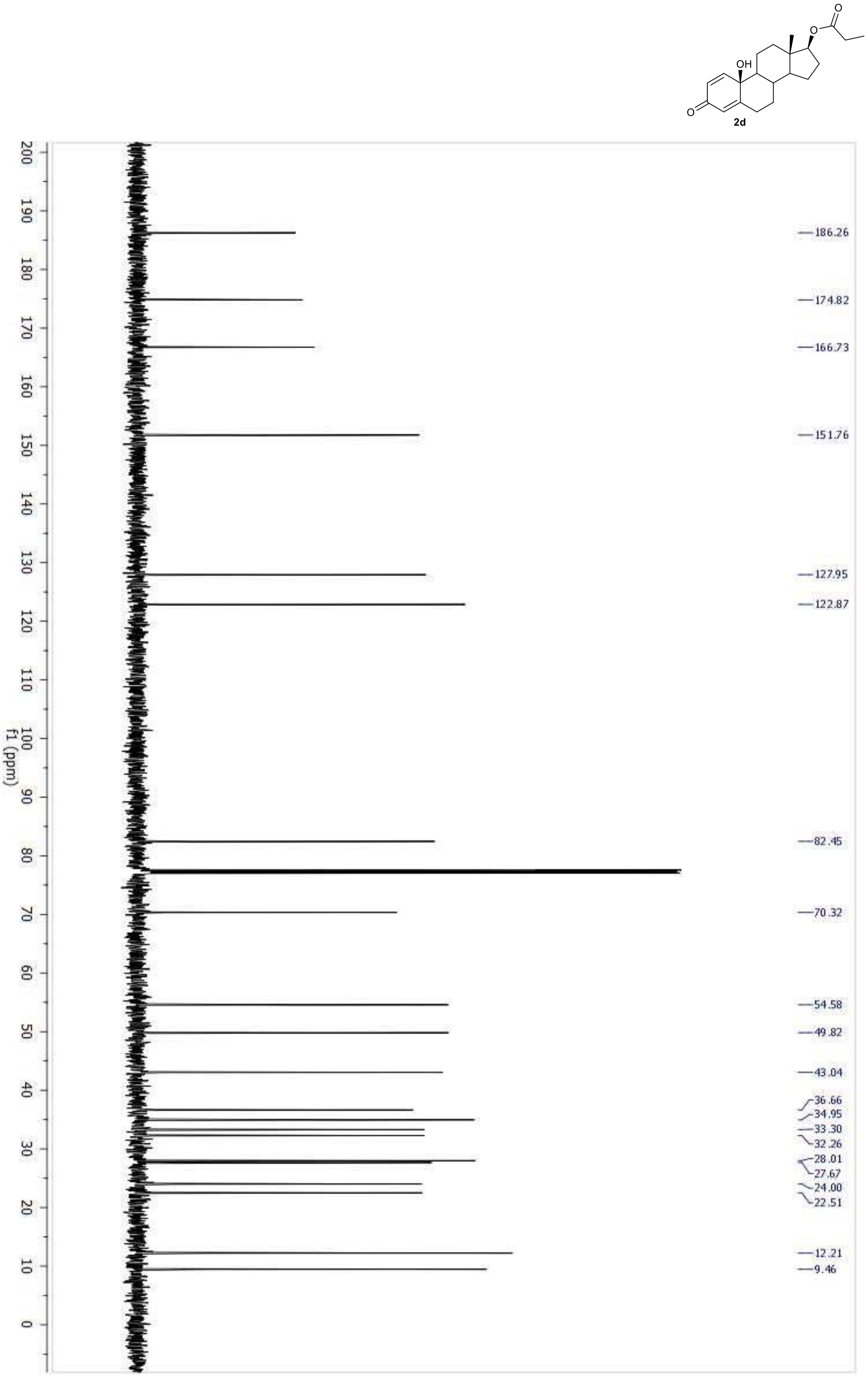

^{13}C NMR spectrum of compound **2d**(125 MHz, $CDCl_3$)

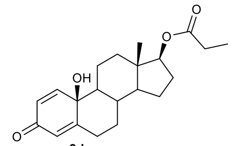

2d

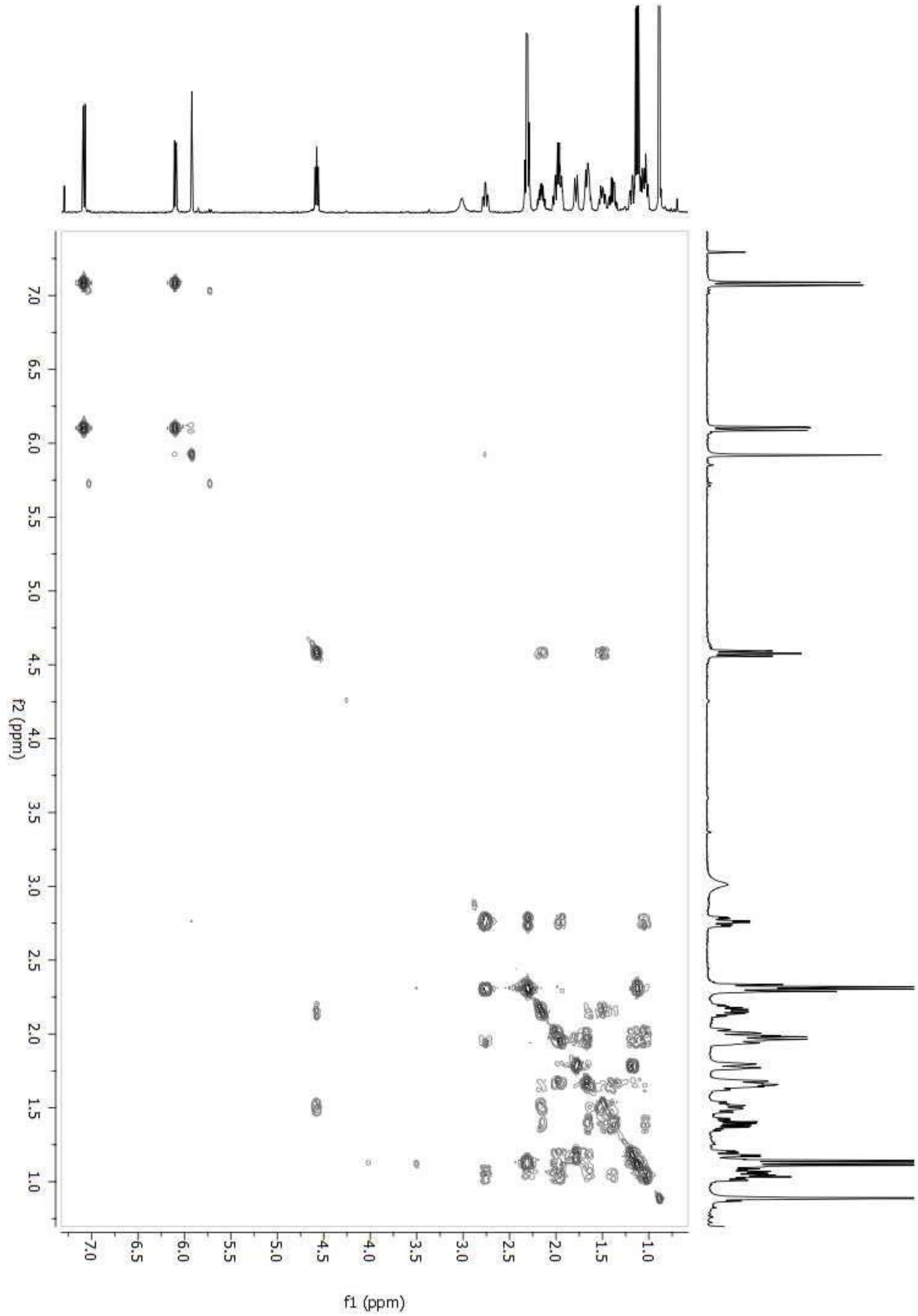

COSY spectrum of compound **2d**($CDCl_3$)

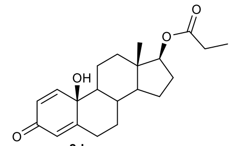

2d

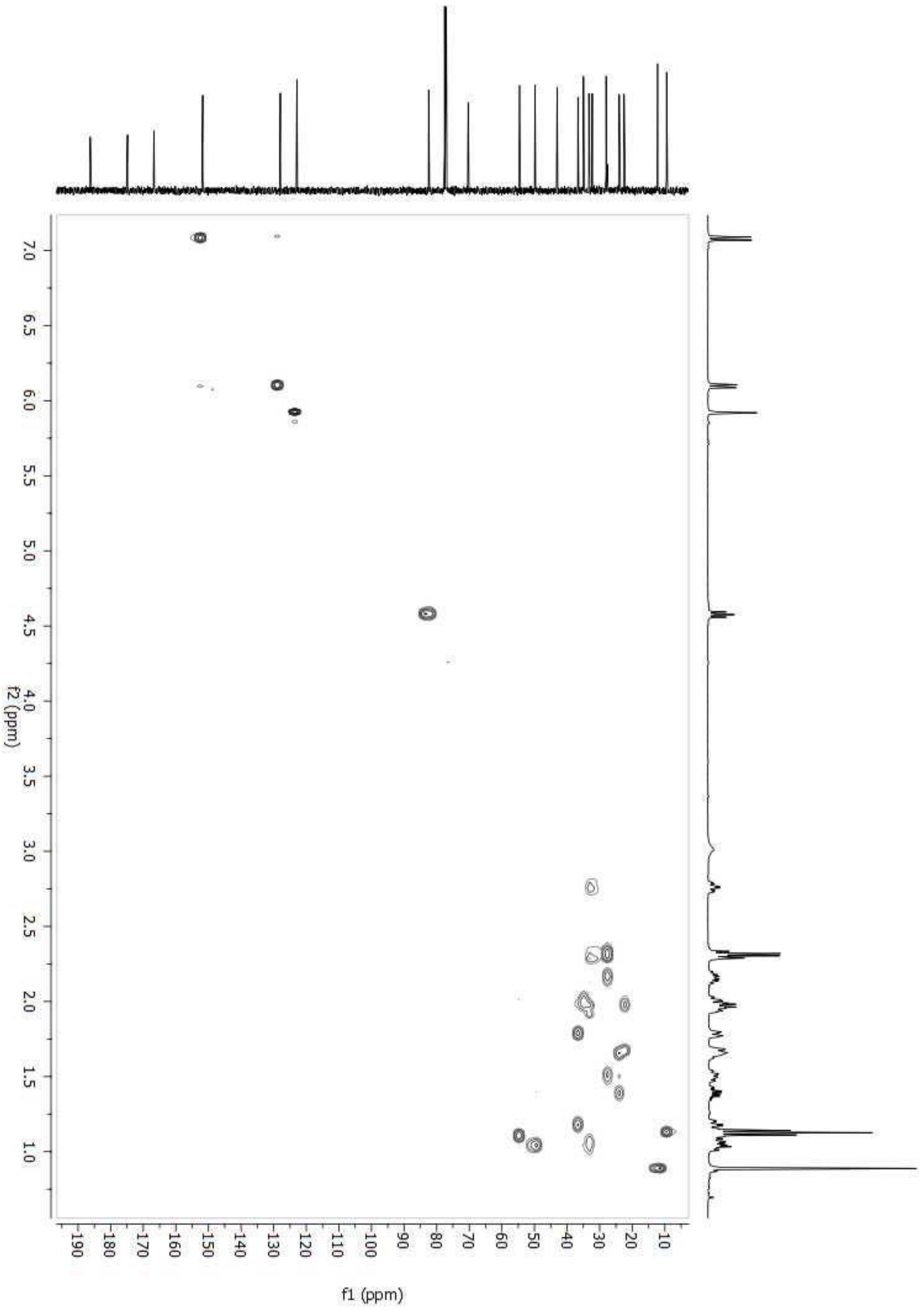

HSQC spectrum of compound **2d**($CDCl_3$)

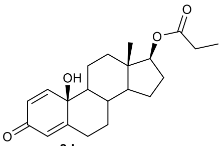

2d

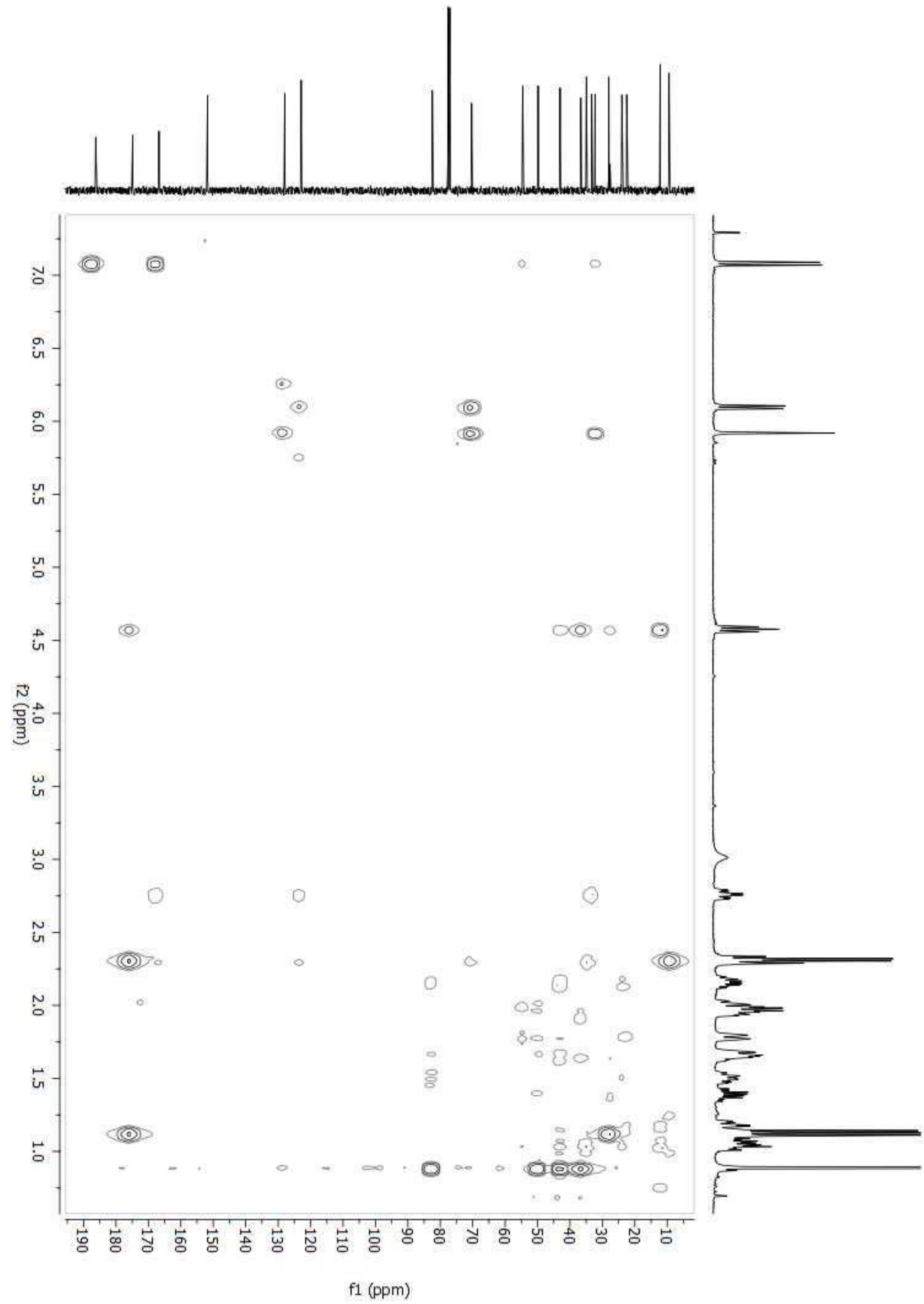

HMBC spectrum of compound **2d**($CDCl_3$)

3.3. Spectra of compound **2e** 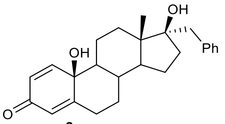 2e

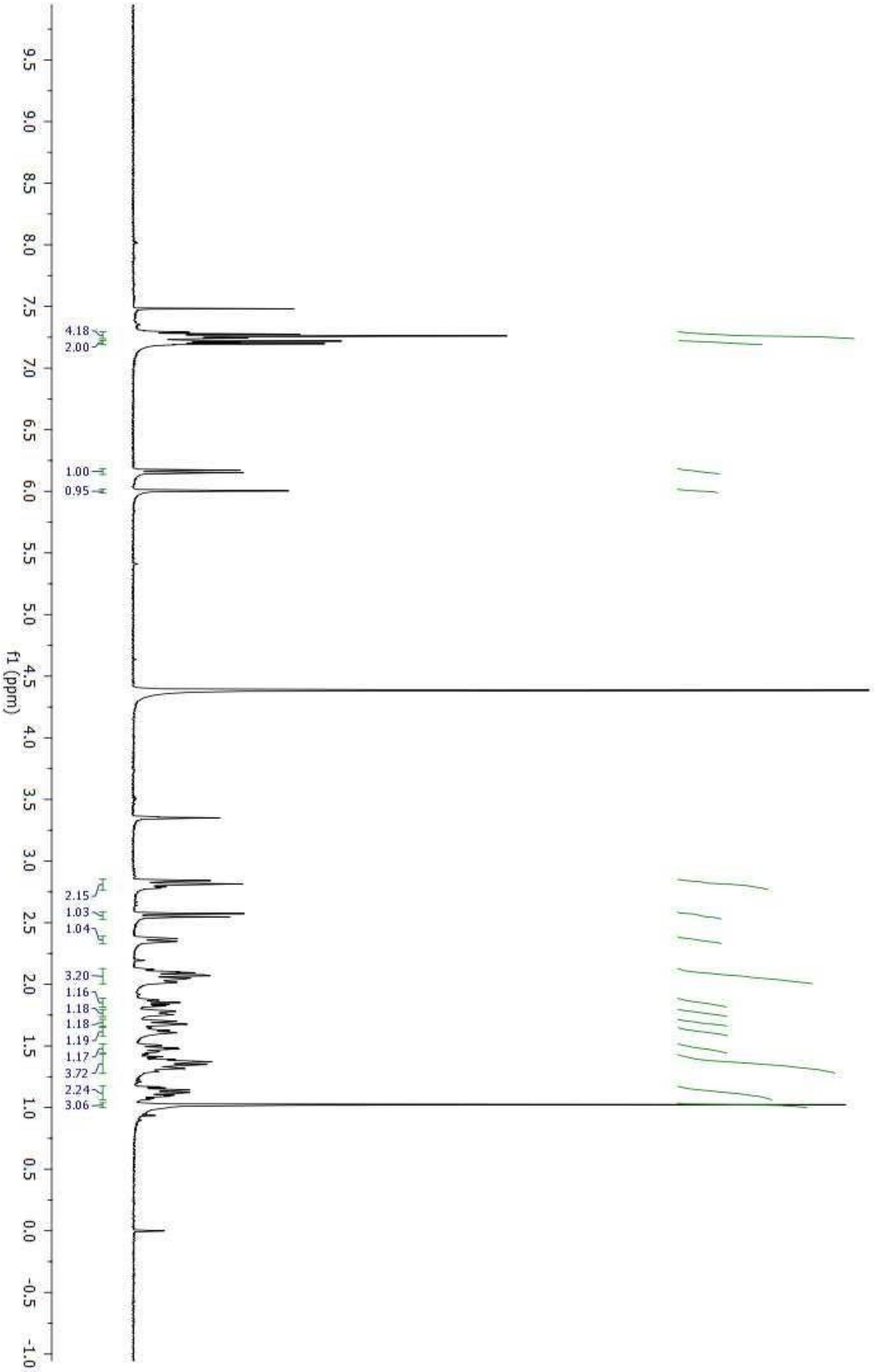

^{1}H NMR spectrum of compound **2e**(500 MHz, $CDCl_3$)

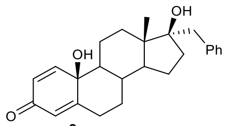

2e

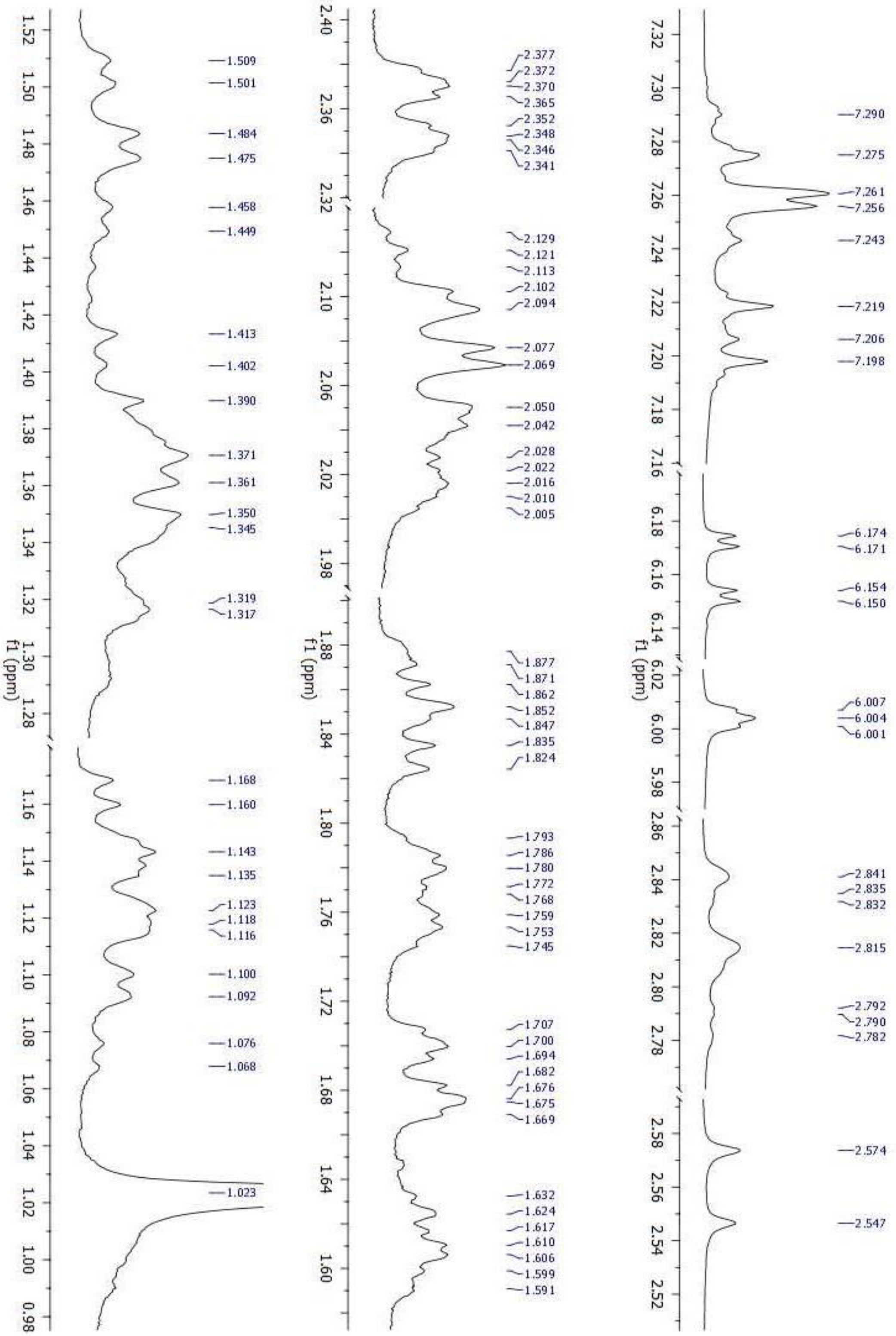

^{1}H NMR spectrum of compound **2e**(500 MHz, $CDCl_3$)

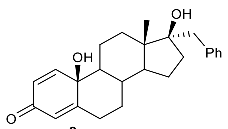

2e

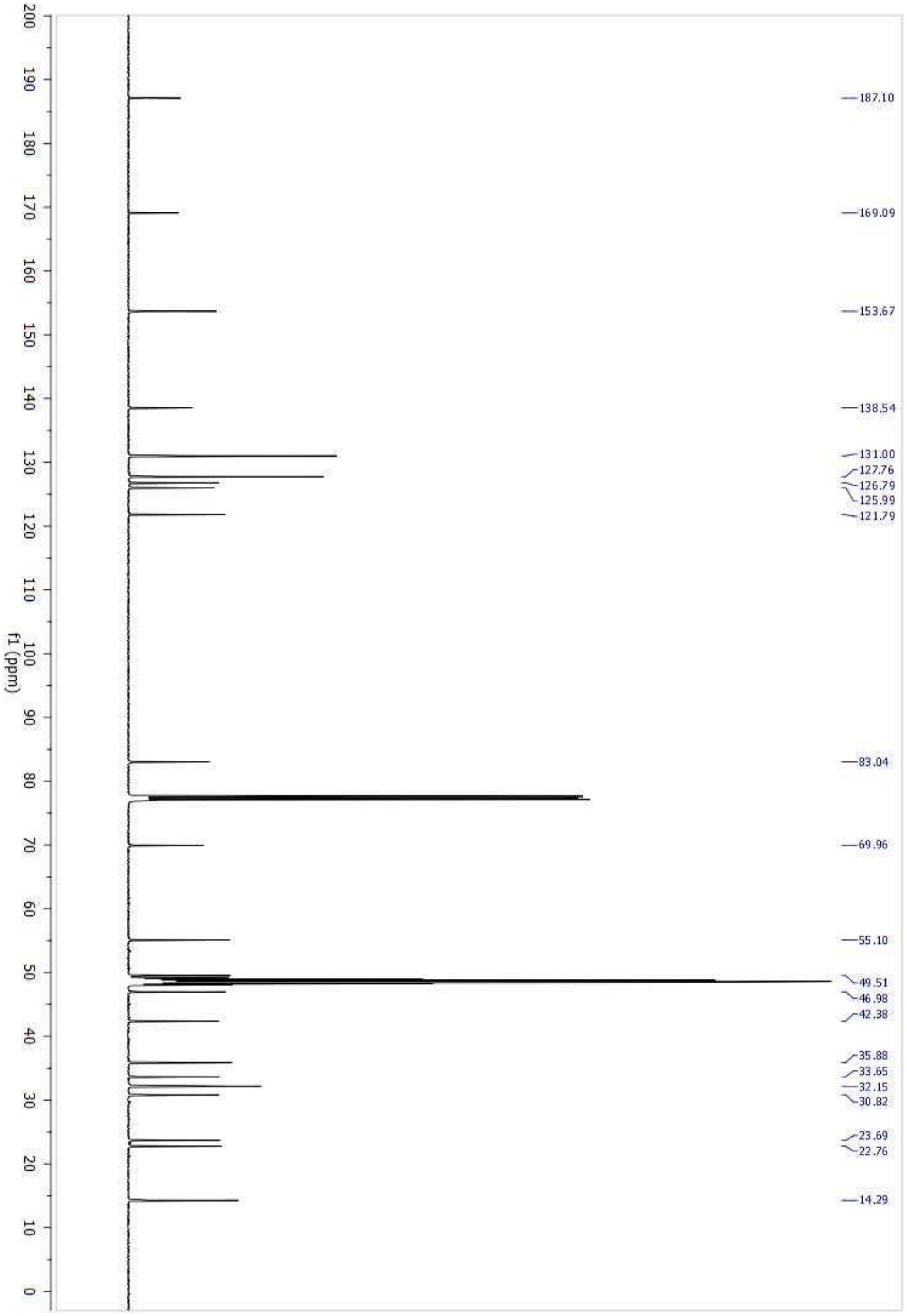

^{13}C NMR spectrum of compound **2e**(125 MHz, $CDCl_3$)

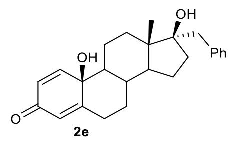

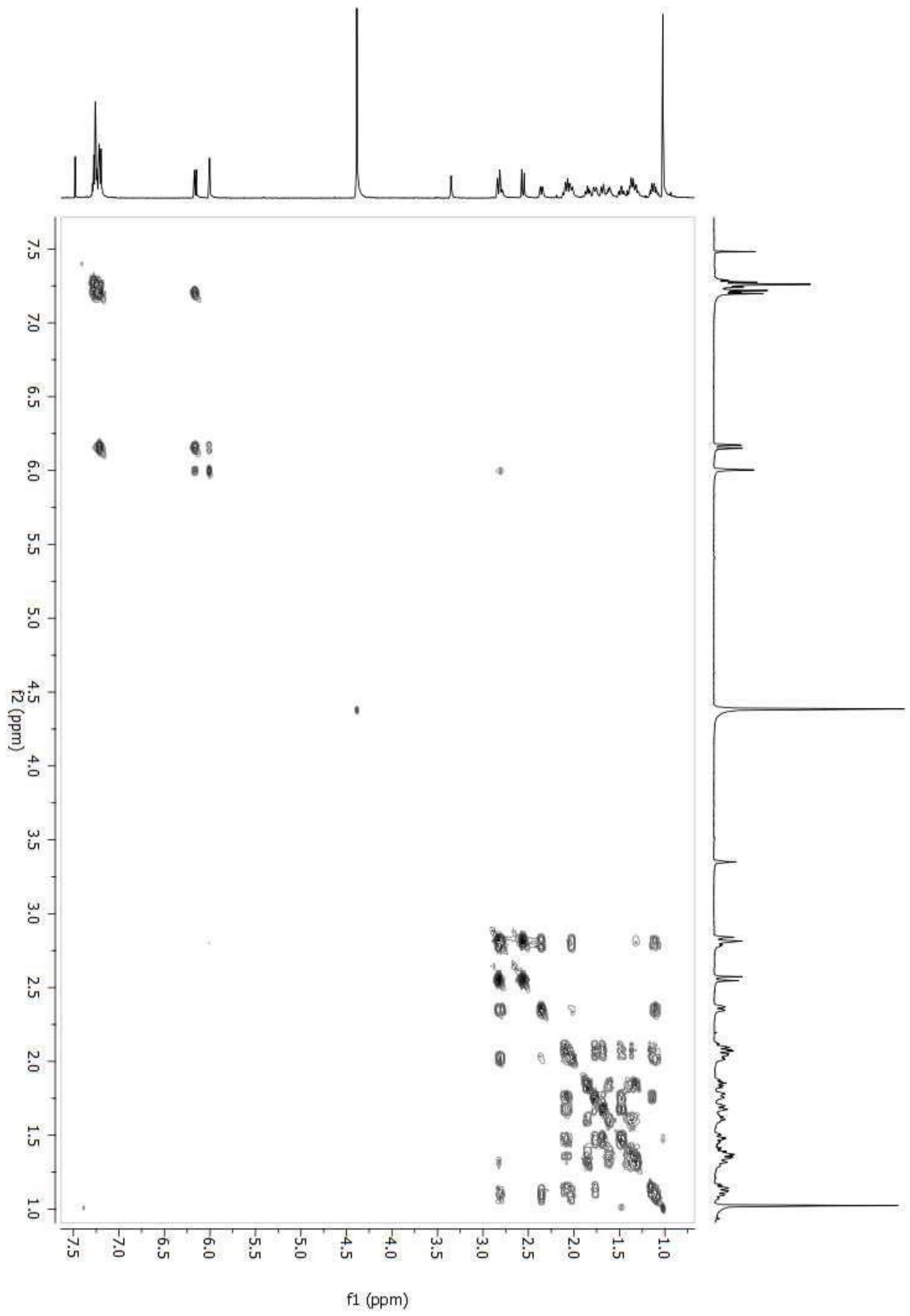

COSY spectrum of compound **2e**($CDCl_3$)

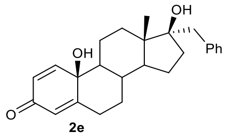

2e

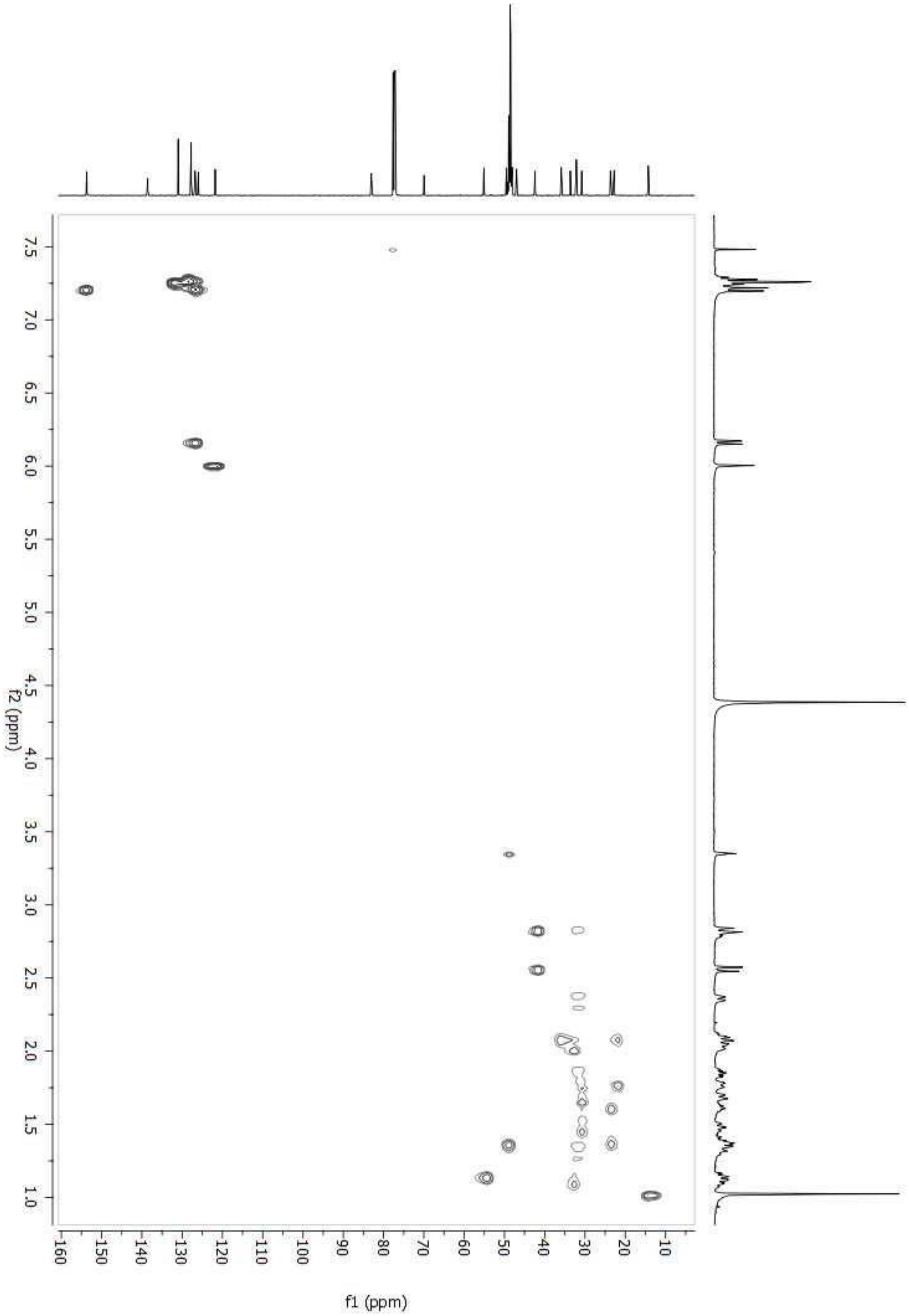

HSQC spectrum of compound **2e**($CDCl_3$)

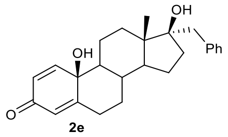

2e

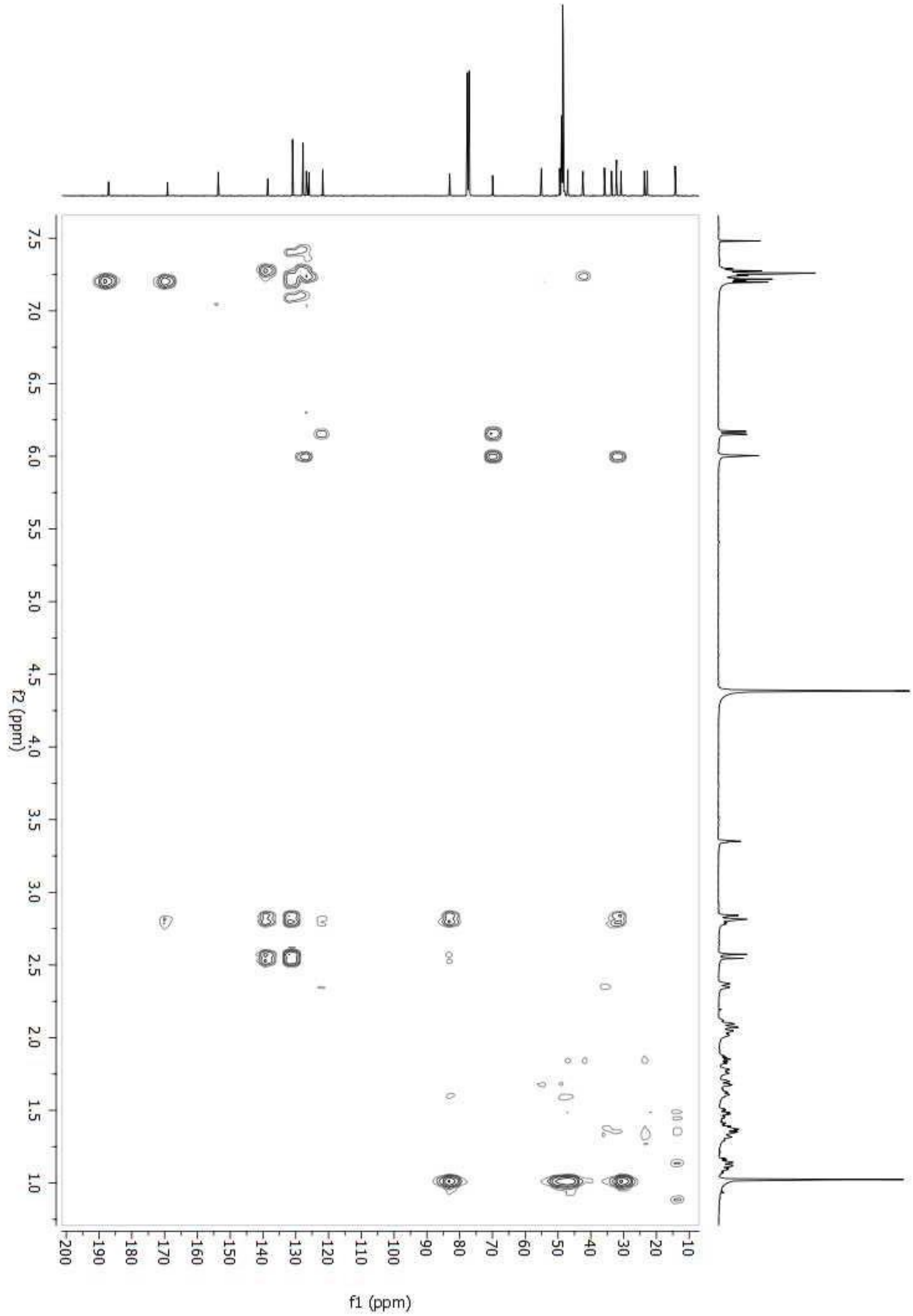

HMBC spectrum of compound **2e**($CDCl_3$)

3.4. Spectra of compound **2f** 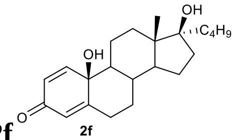

2f

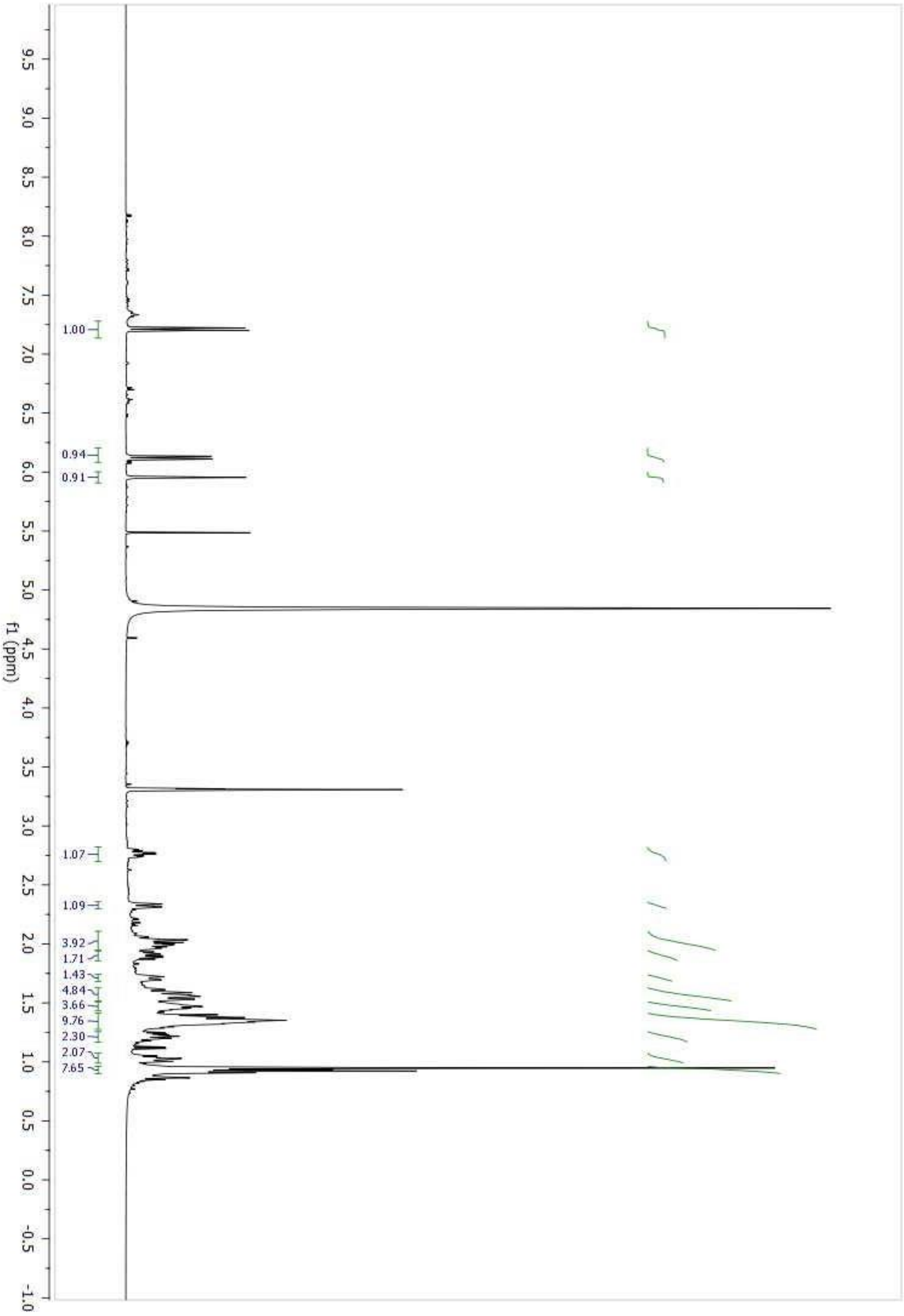

^{1}H NMR spectrum of compound **2f**(500 MHz, Methanol-d_4)

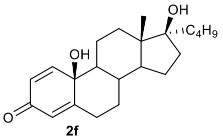

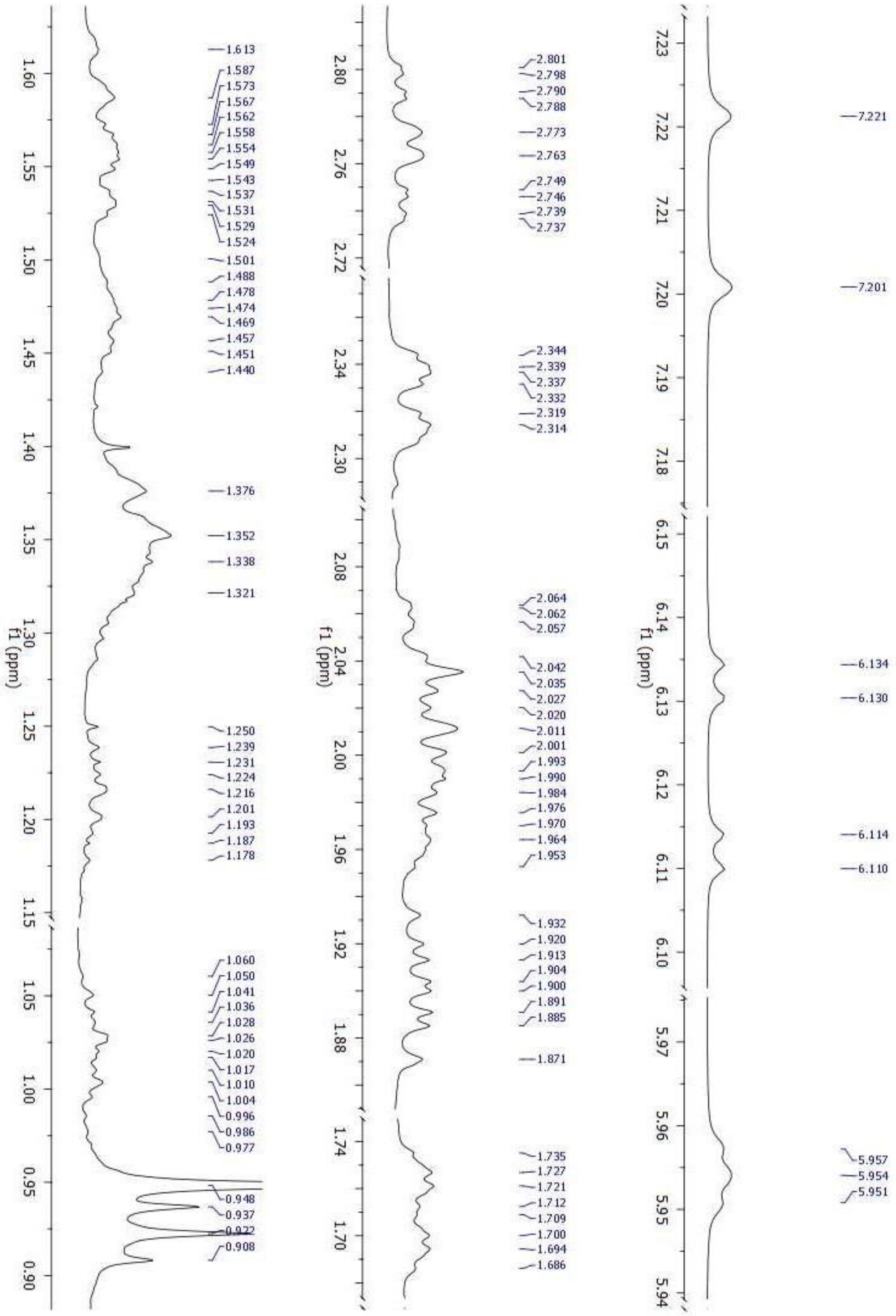

^{1}H NMR spectrum of compound **2f**(500 MHz, Methanol-d_4)

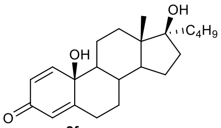

2f

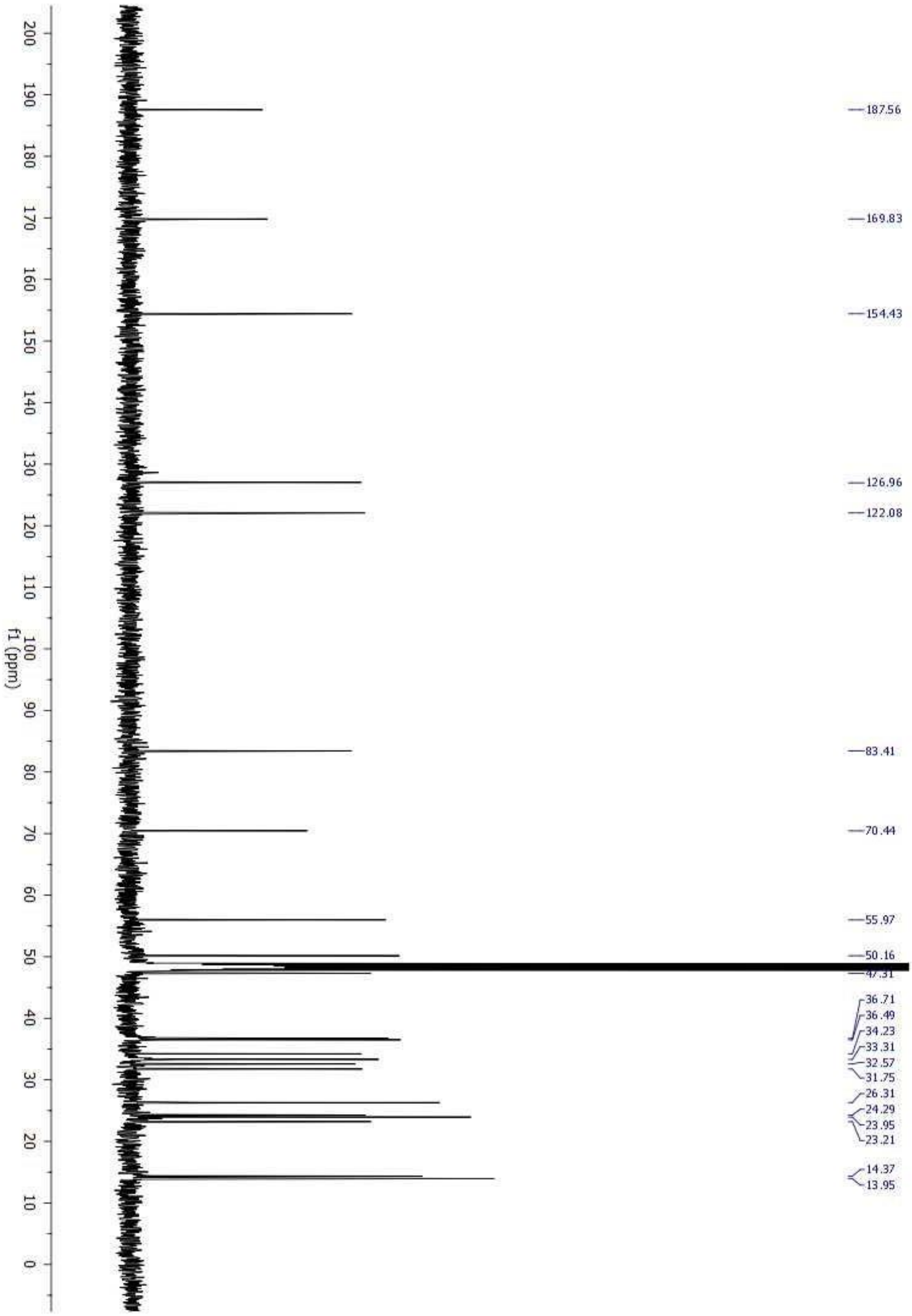

^{13}C NMR spectrum of compound **2f**(125 MHz, Methanol-d_4)

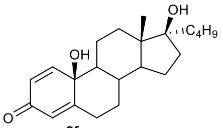

2f

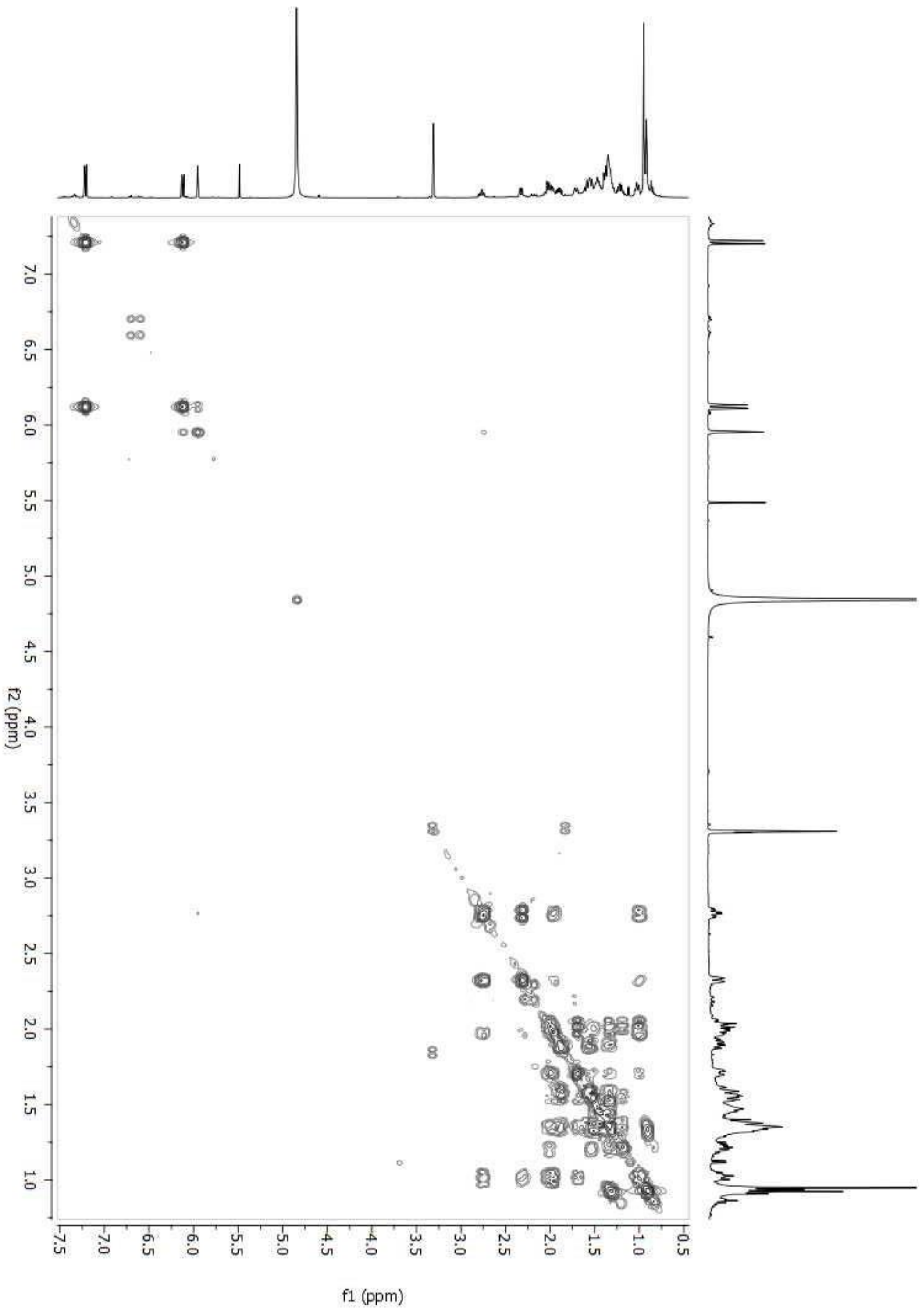

COSY spectrum of compound **2f**(Methanol-d_4)

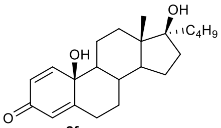

2f

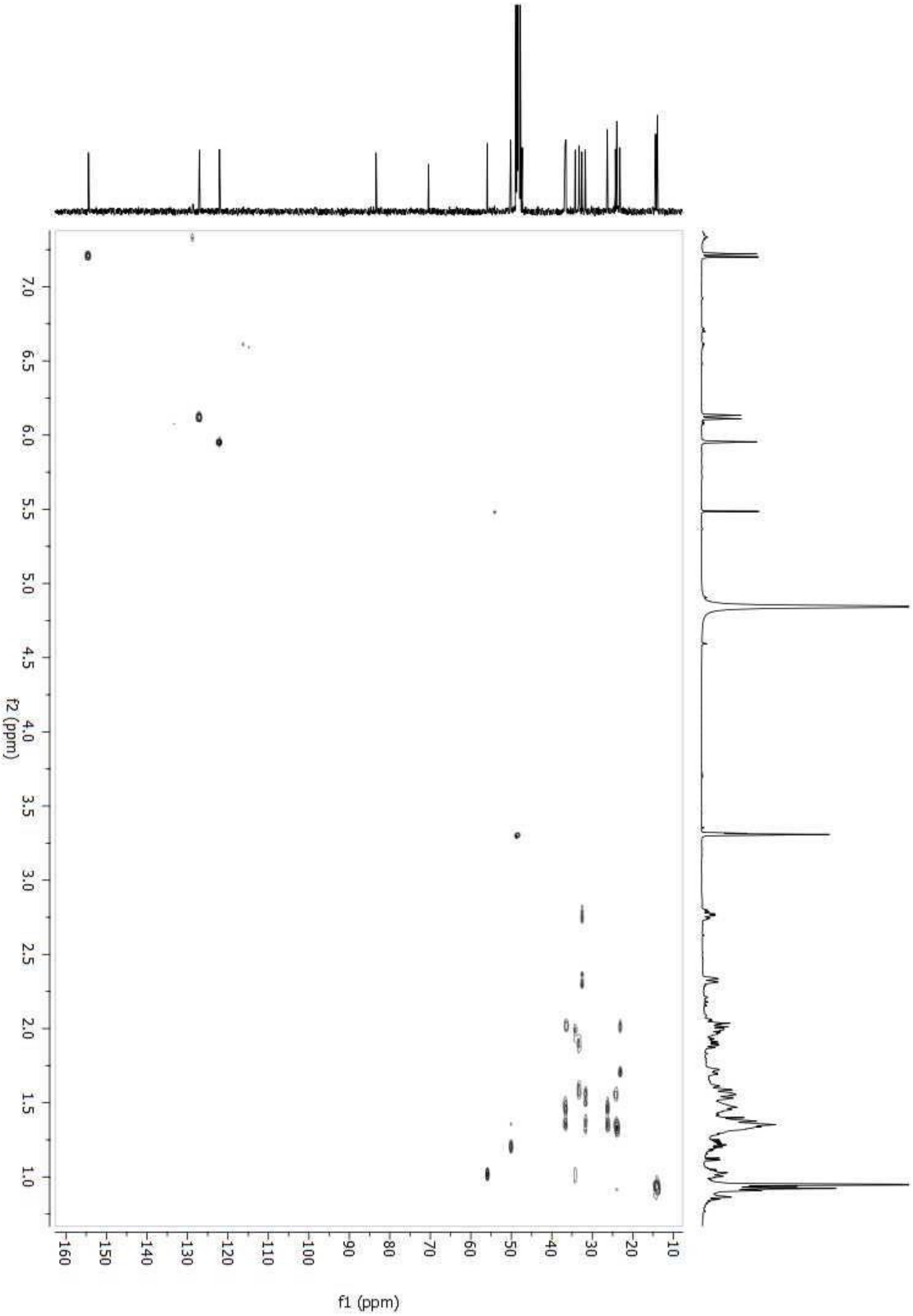

HSQC cpectrum of compound **2f**(Methanol-d_4)

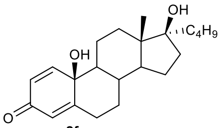

2f

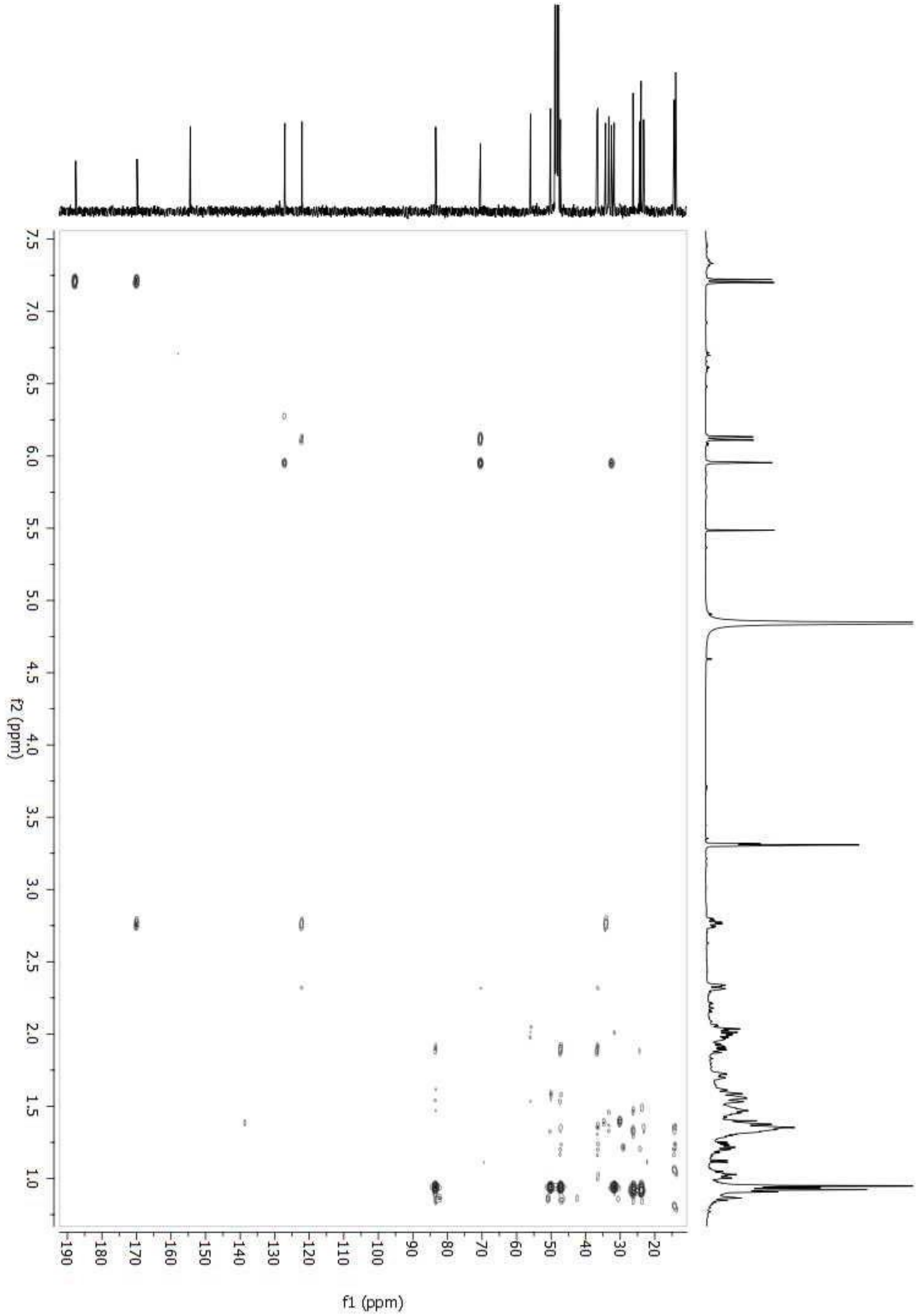

HMBC spectrum of compound **2f**(Methanol-*d_4*)

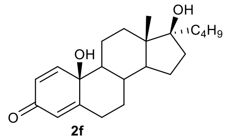

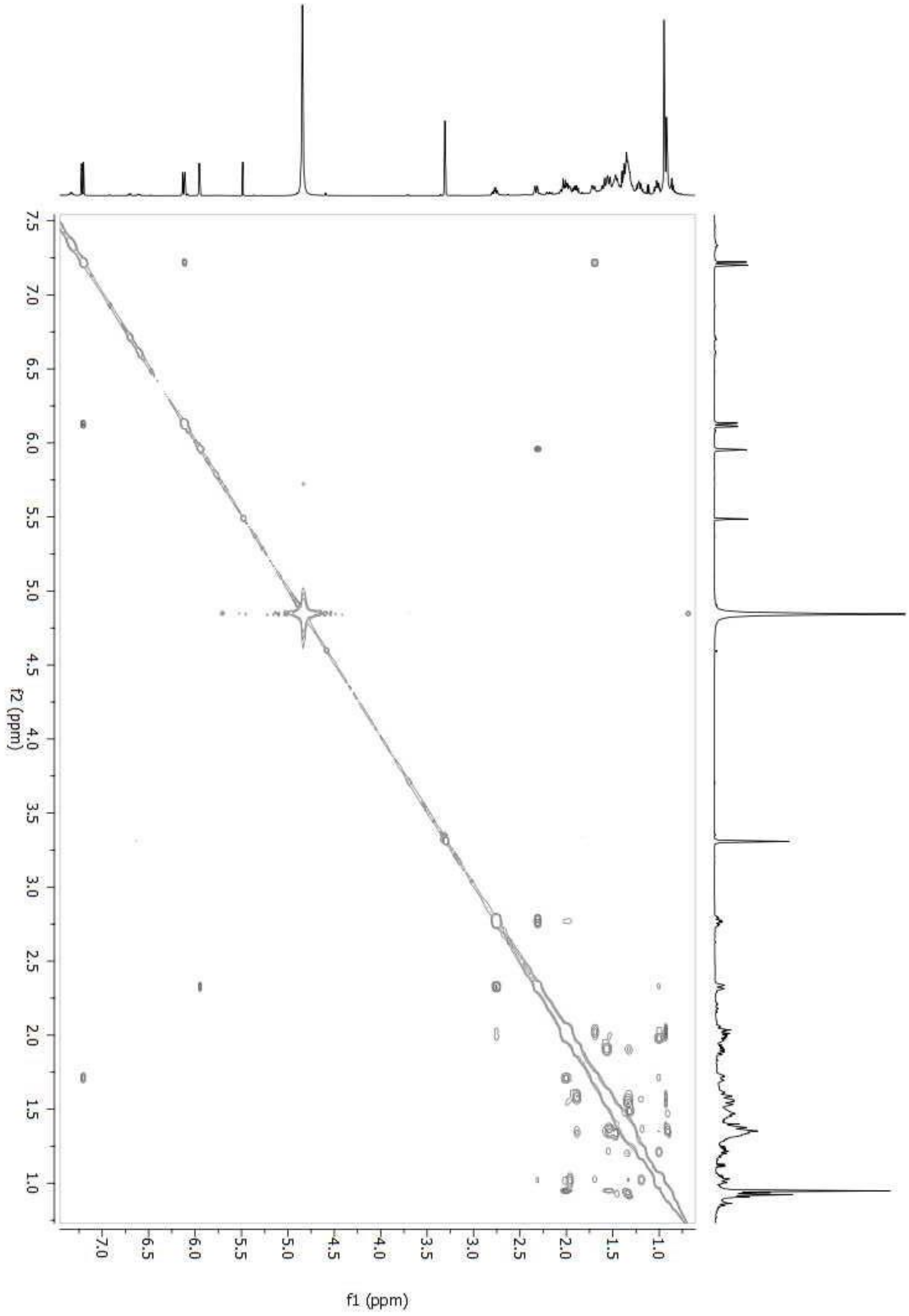

NOESY spectrum of compound **2f**(Methanol-d_4)

3.5. Spectra of compound **3b** 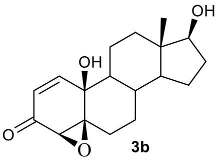

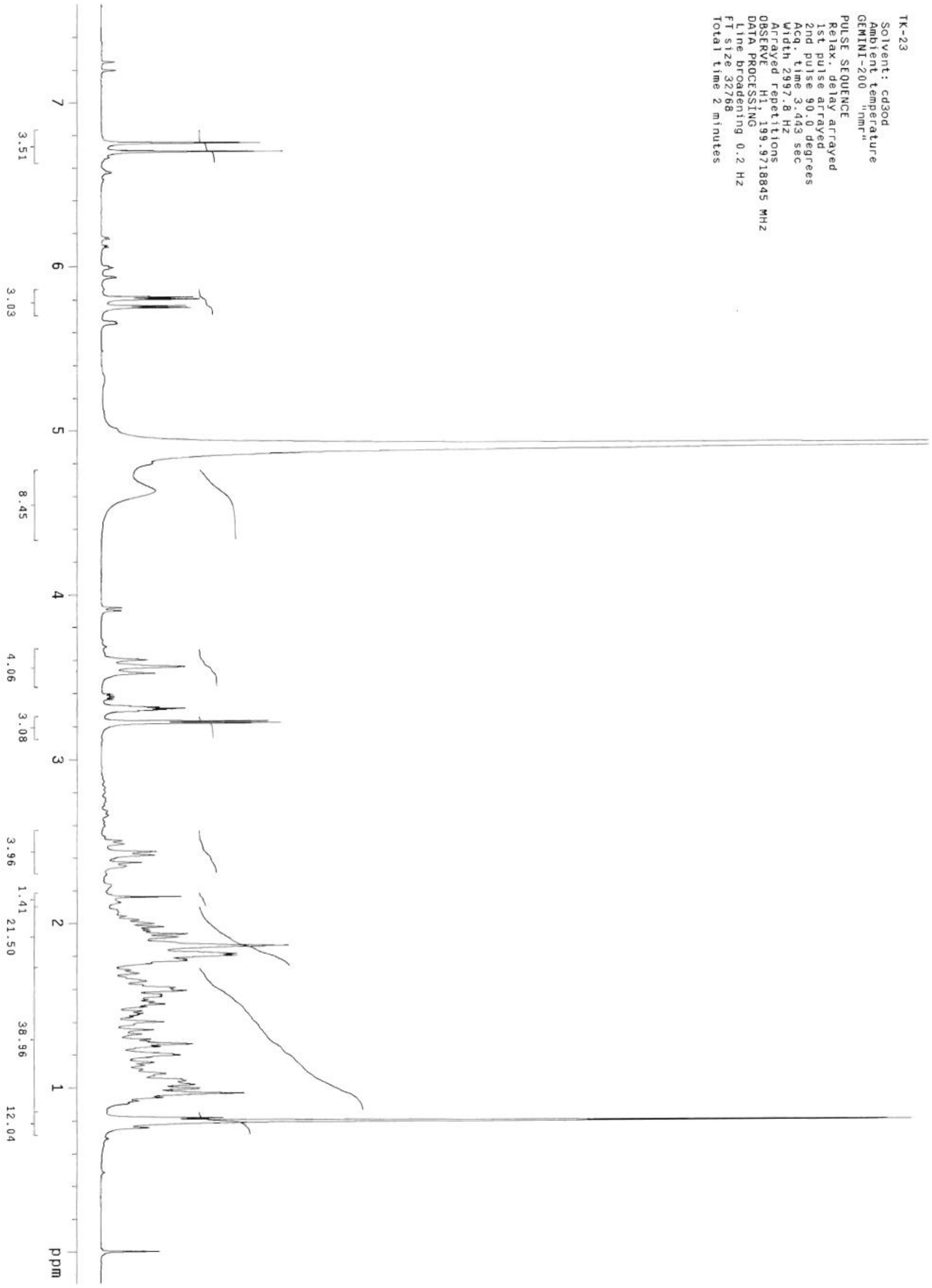

^{1}H NMR spectrum of compound**3b**(200 MHz, Acetone-d_6)

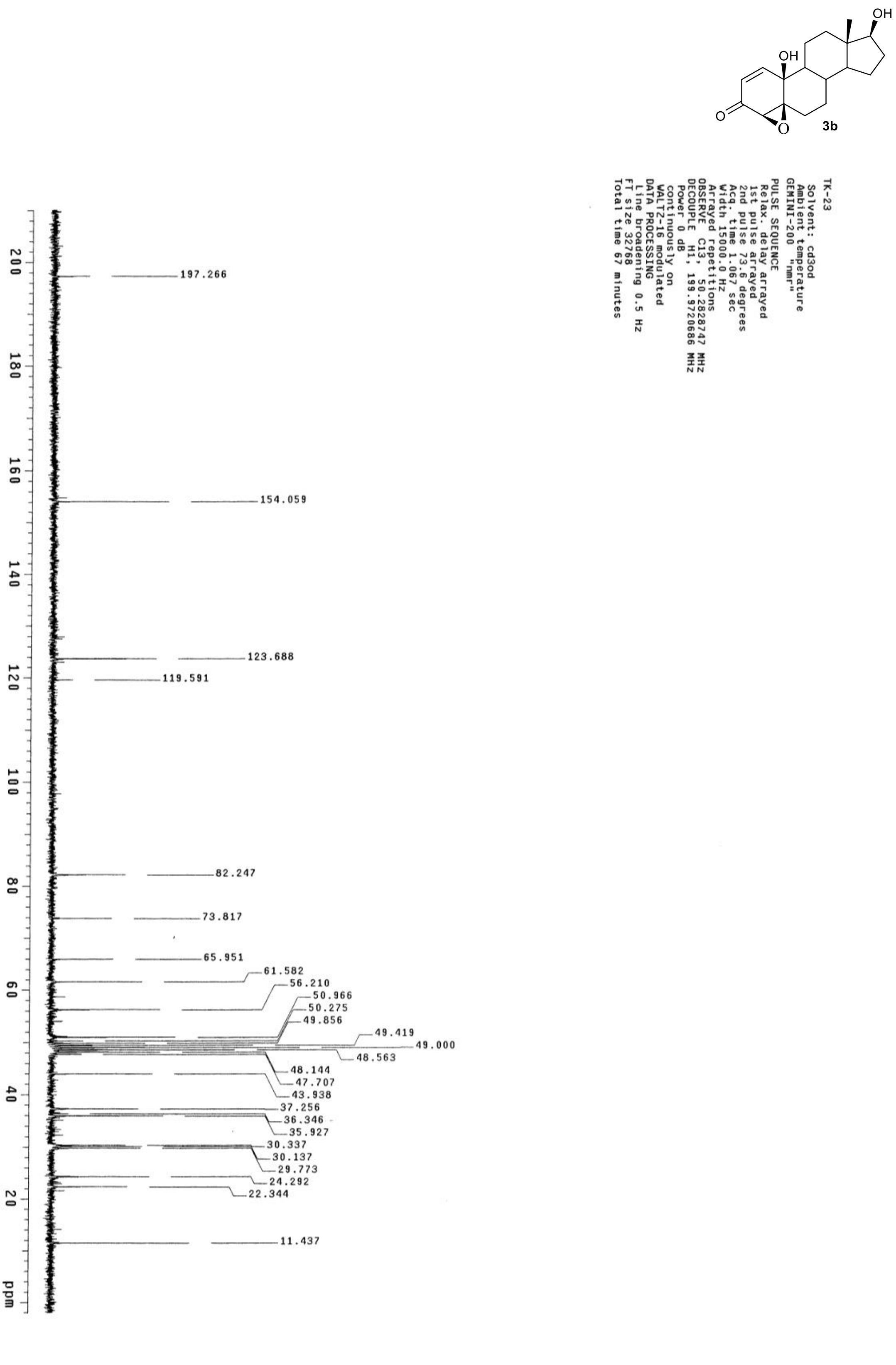

^{13}C NMR spectrum of compound**3b**(50 MHz, Acetone d_6)

3.6. Spectra of compound **3d** 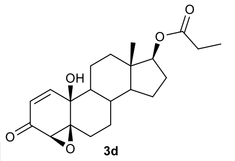

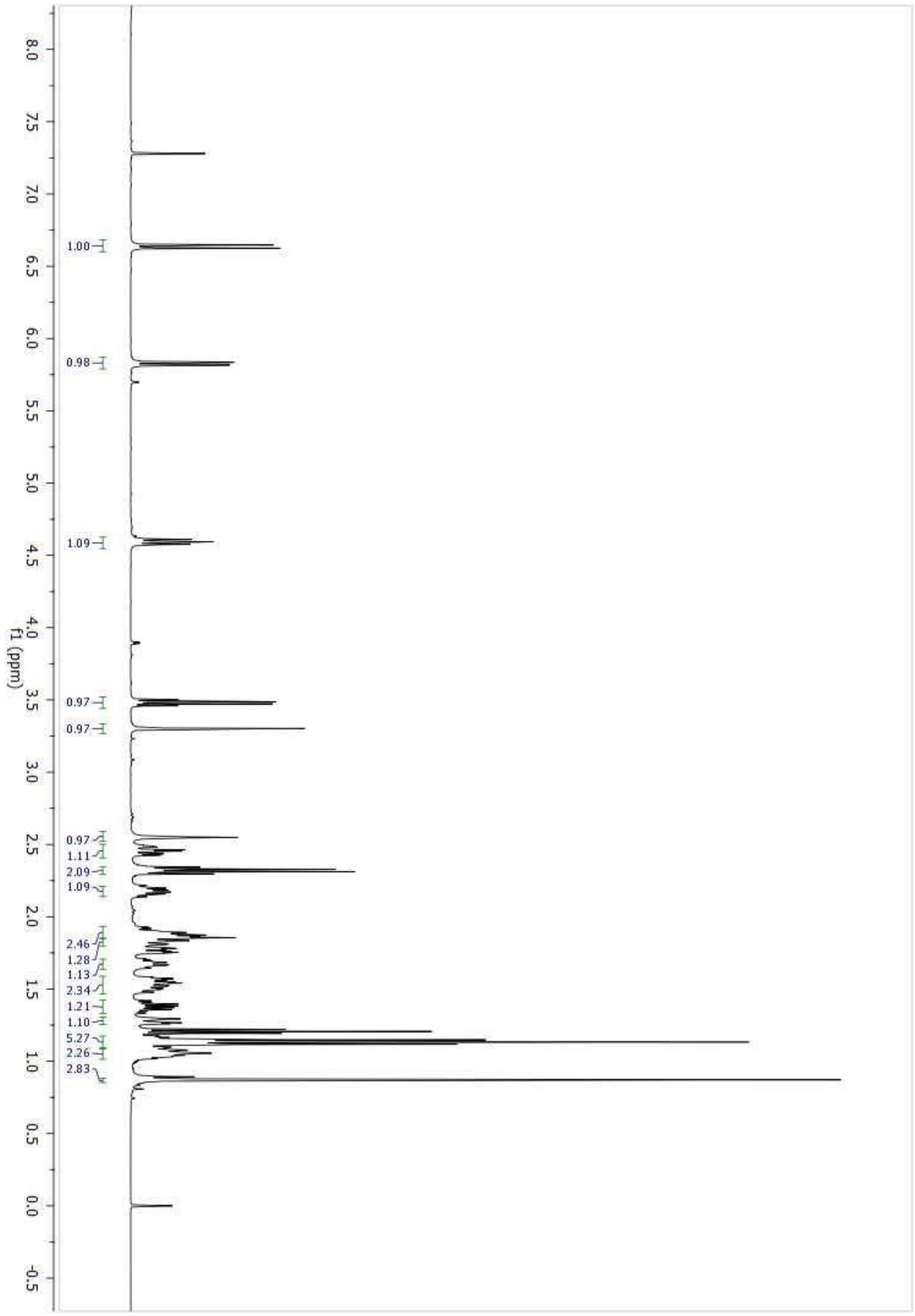

^{1}H NMR spectrum of compound**3d**(500 MHz, $CDCl_3$)

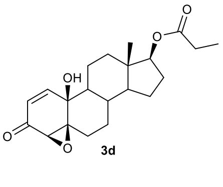

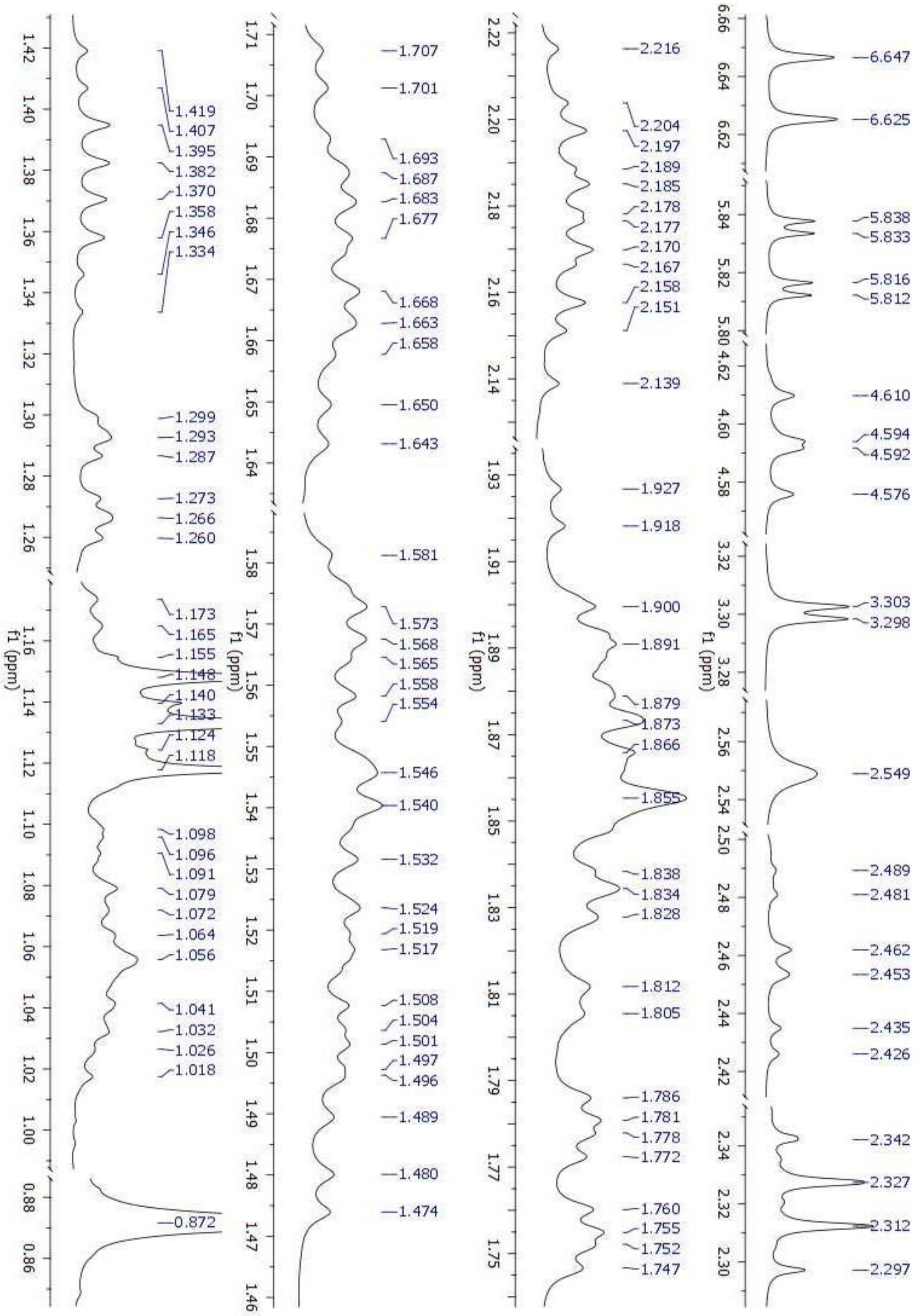

^{1}H NMR spectrum of compound**3d**(500 MHz, $CDCl_3$)

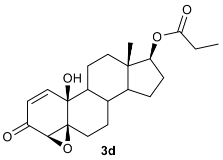

3d

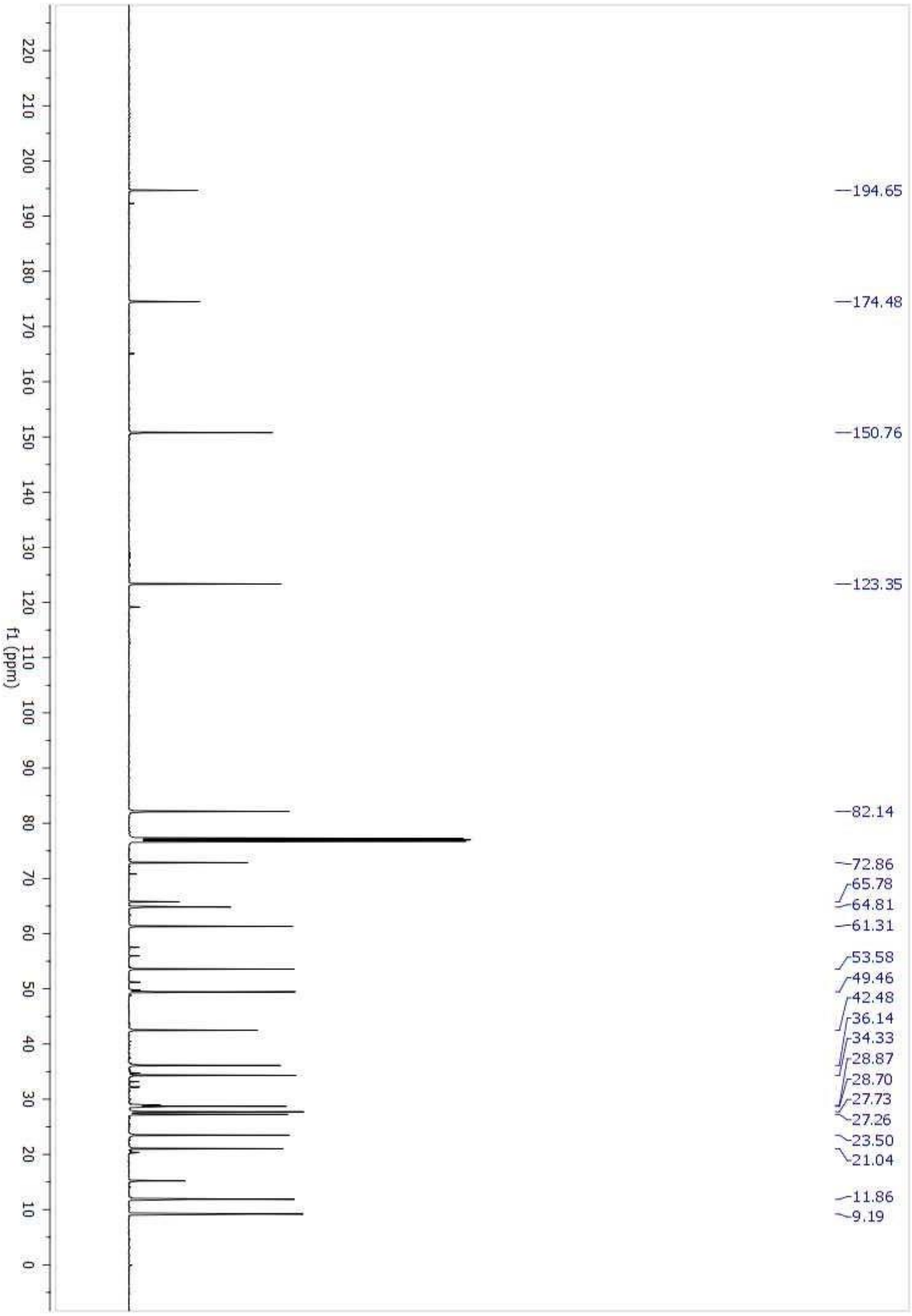

^{13}C NMR spectrum of compound**3d**(125 MHz, $CDCl_3$)

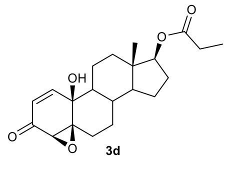

3d

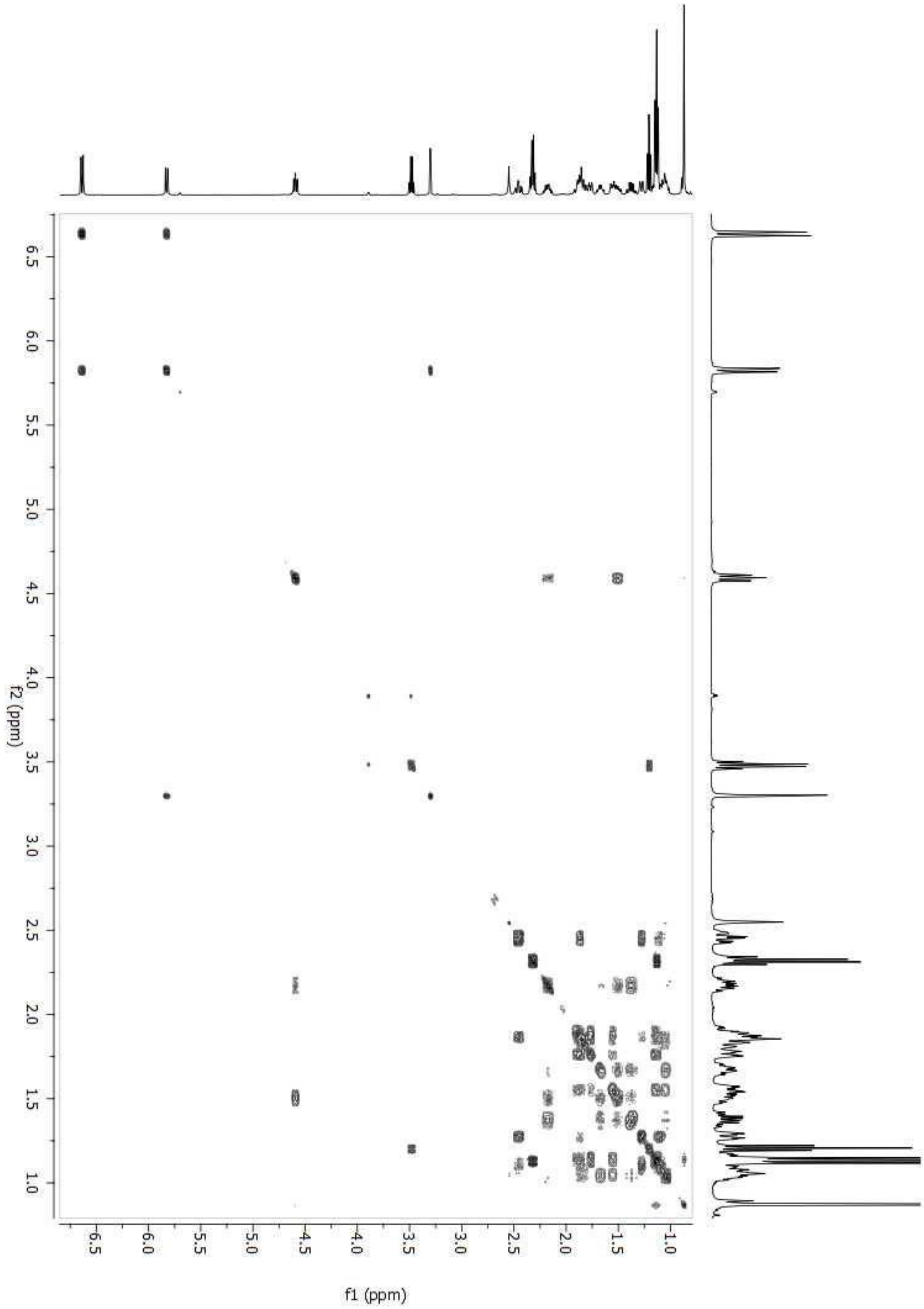

COSY spectrum of compound **3d**($CDCl_3$)

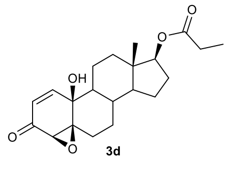

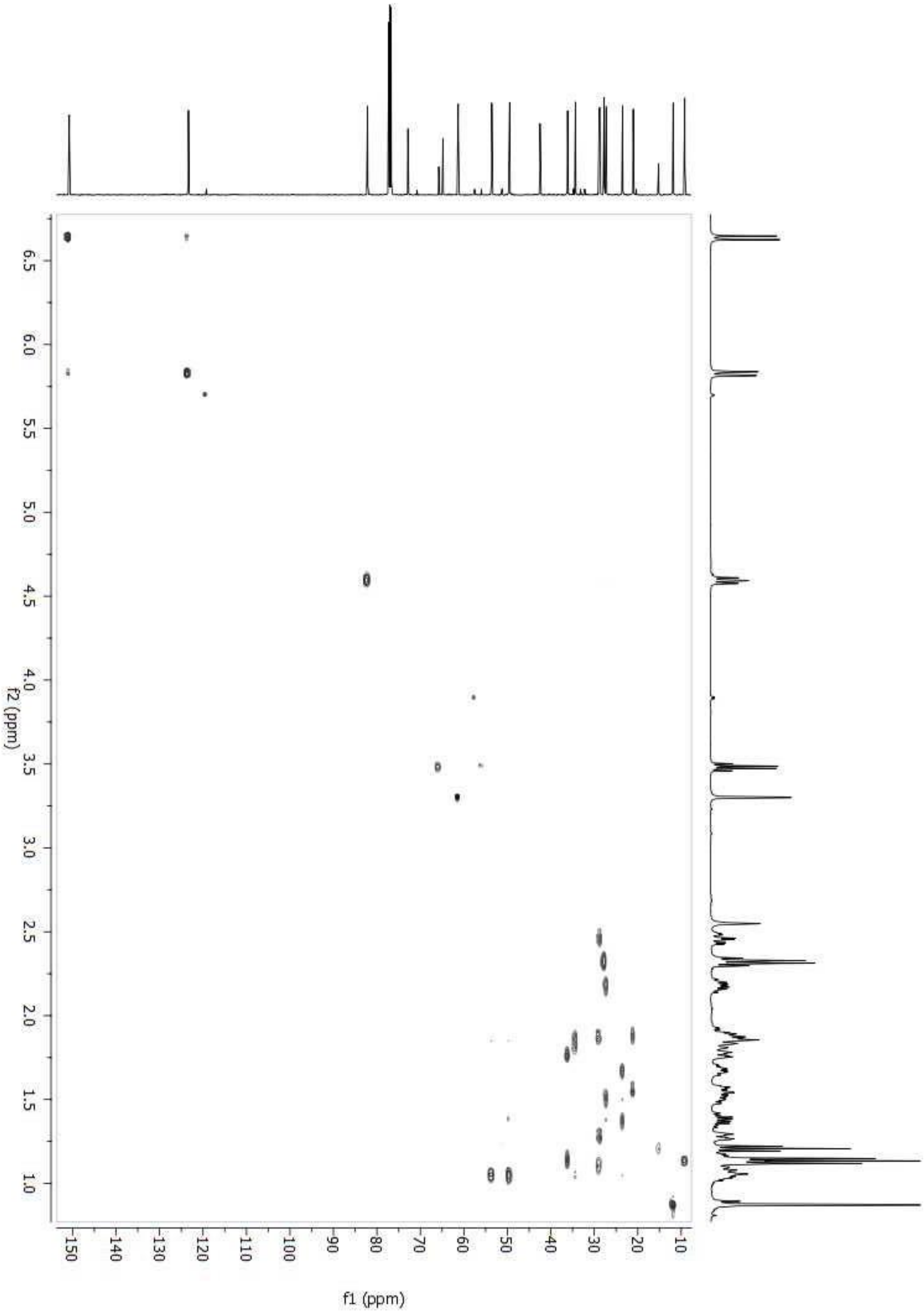

HSQC spectrum of compound **3d**($CDCl_3$)

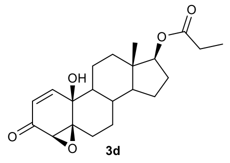

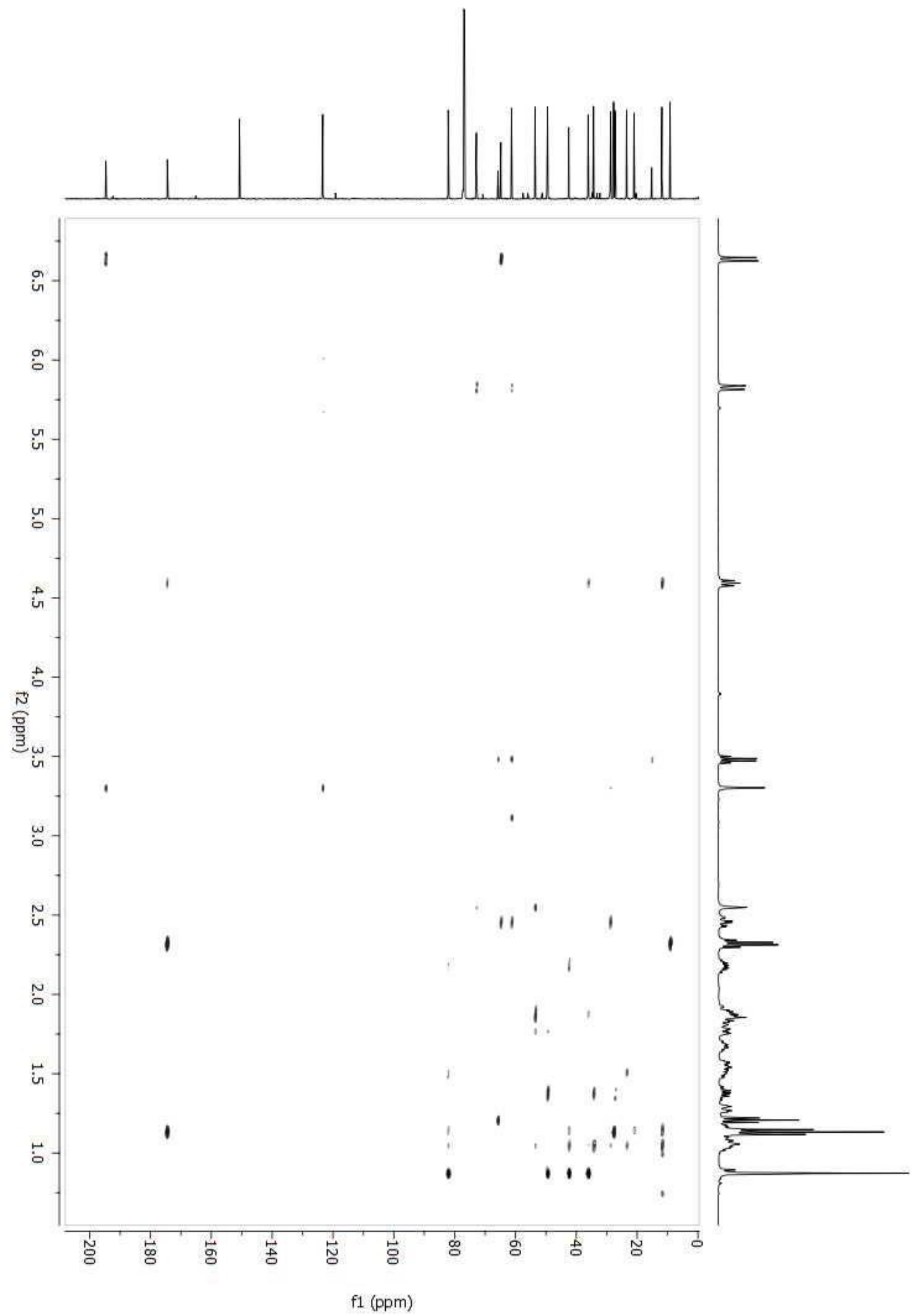

HMBC spectrum of compound **3d**($CDCl_3$)

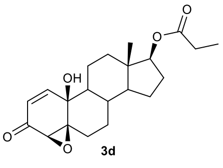

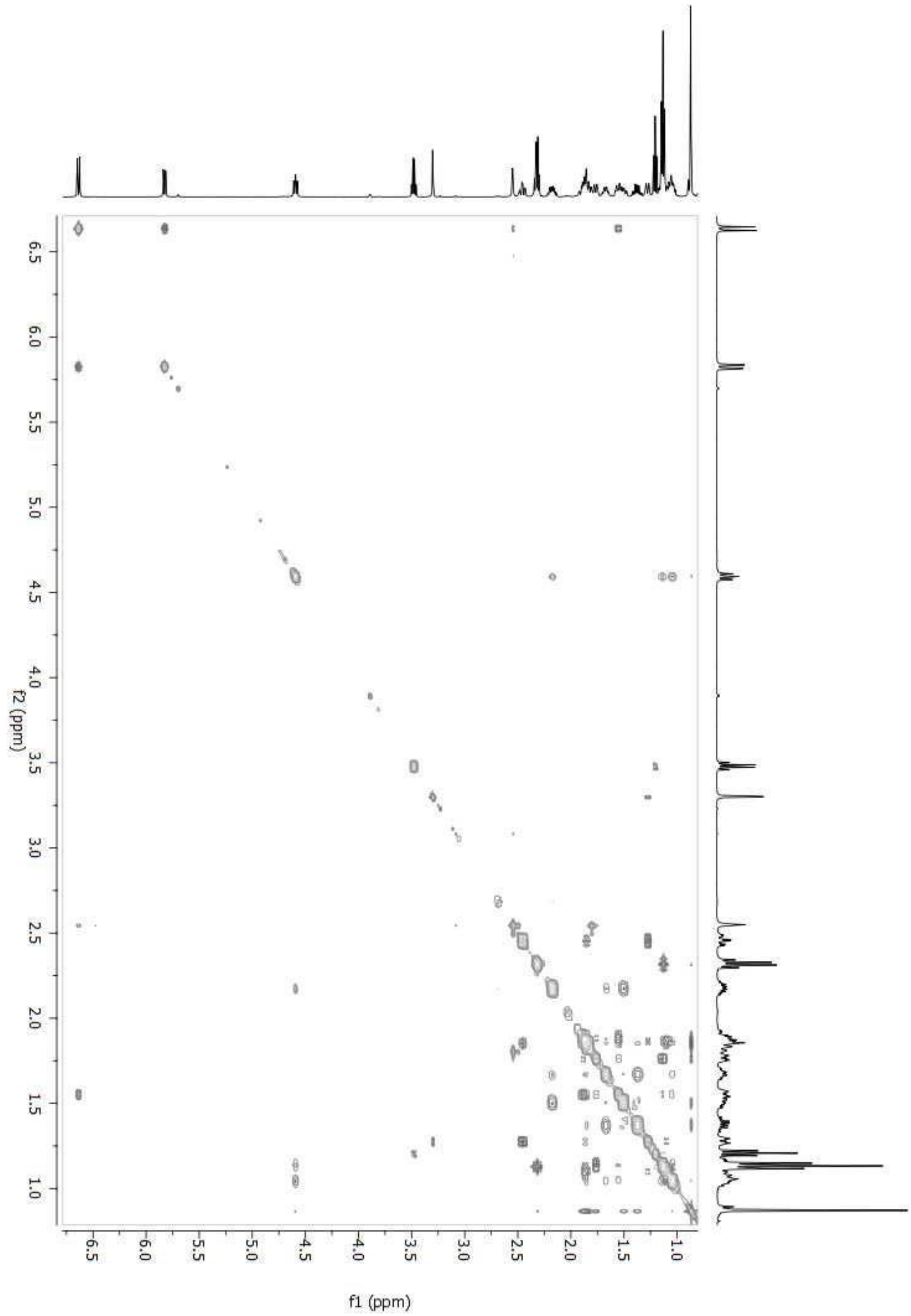

NOESY spectrum of compound **3d**($CDCl_3$)

3.7. Spectra of compound **3e** 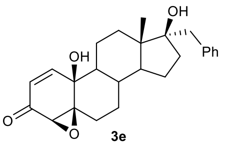

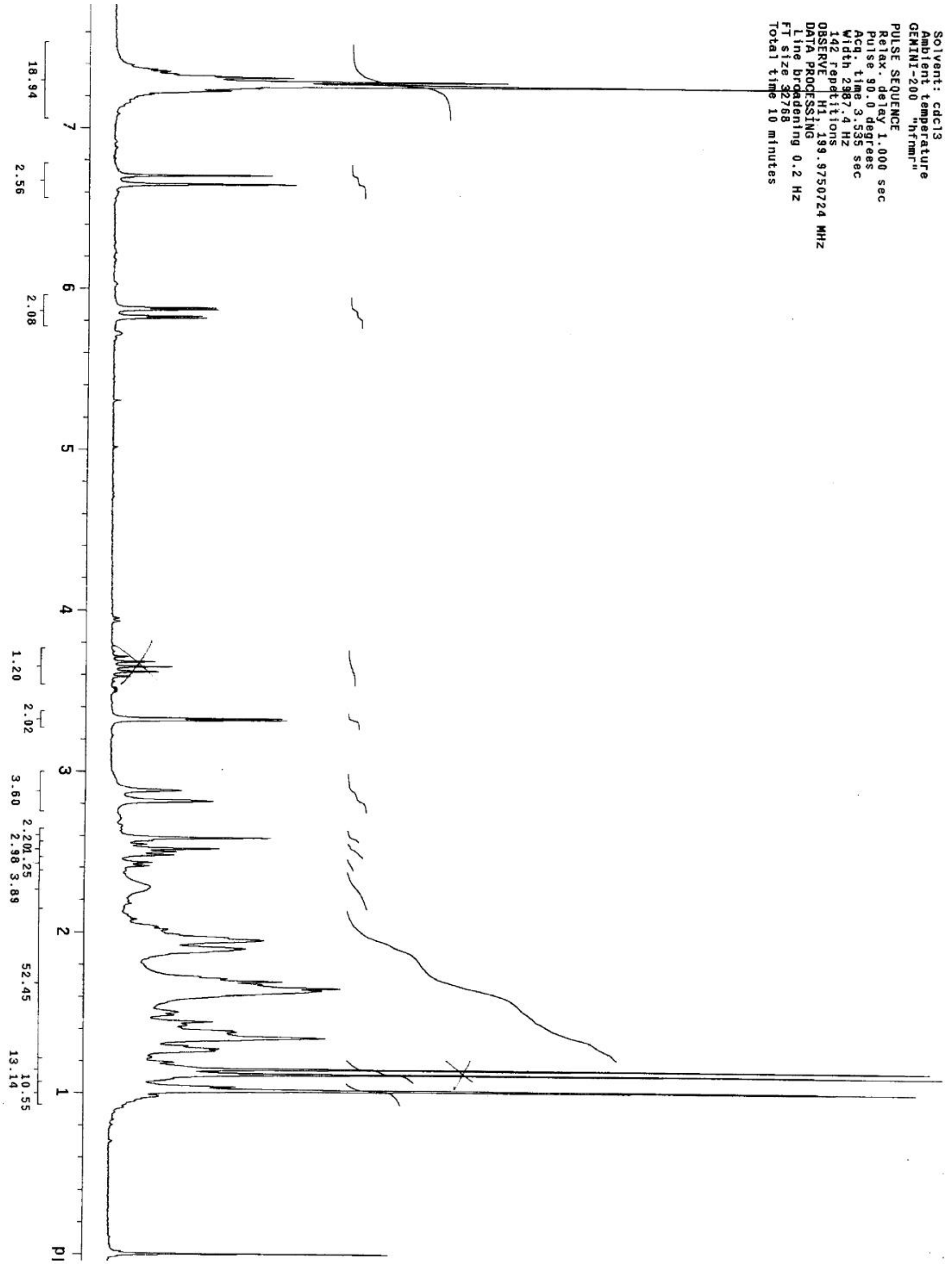

^{1}H NMR spectrum of compound**3e**(200 MHz, $CDCl_3$)

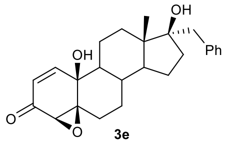

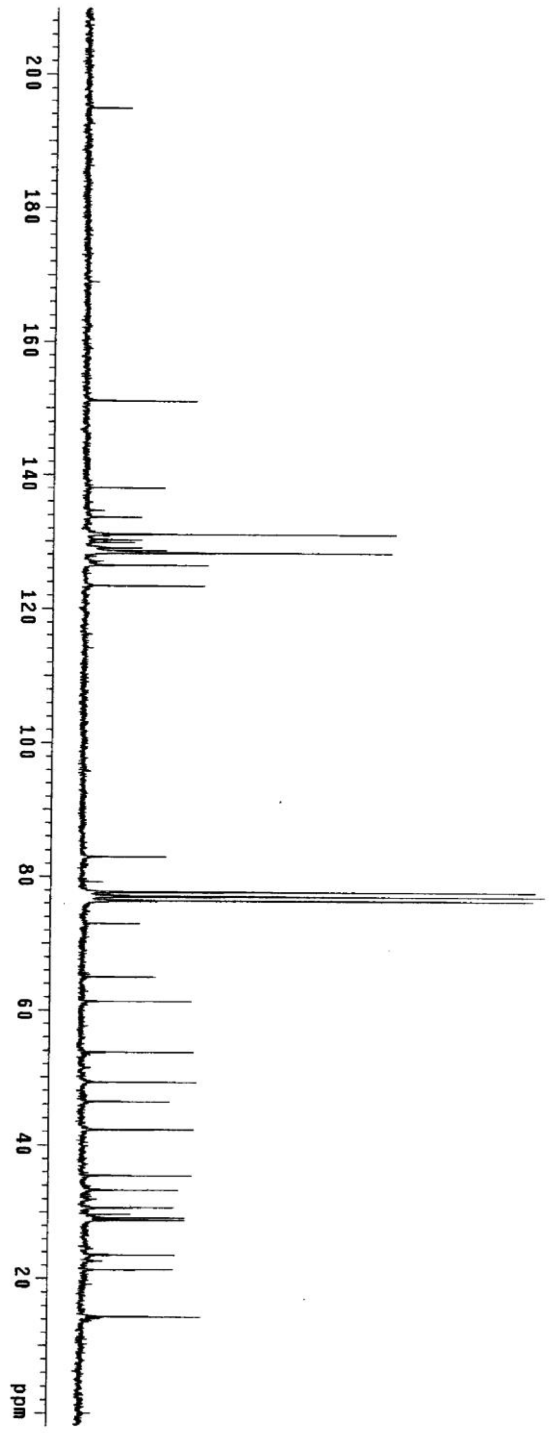

^{13}C NMR spectrum of compound**3e**(50 MHz, $CDCl_3$)

3.8. Spectra of compound **3f** 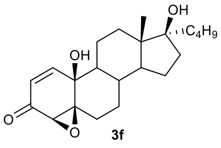

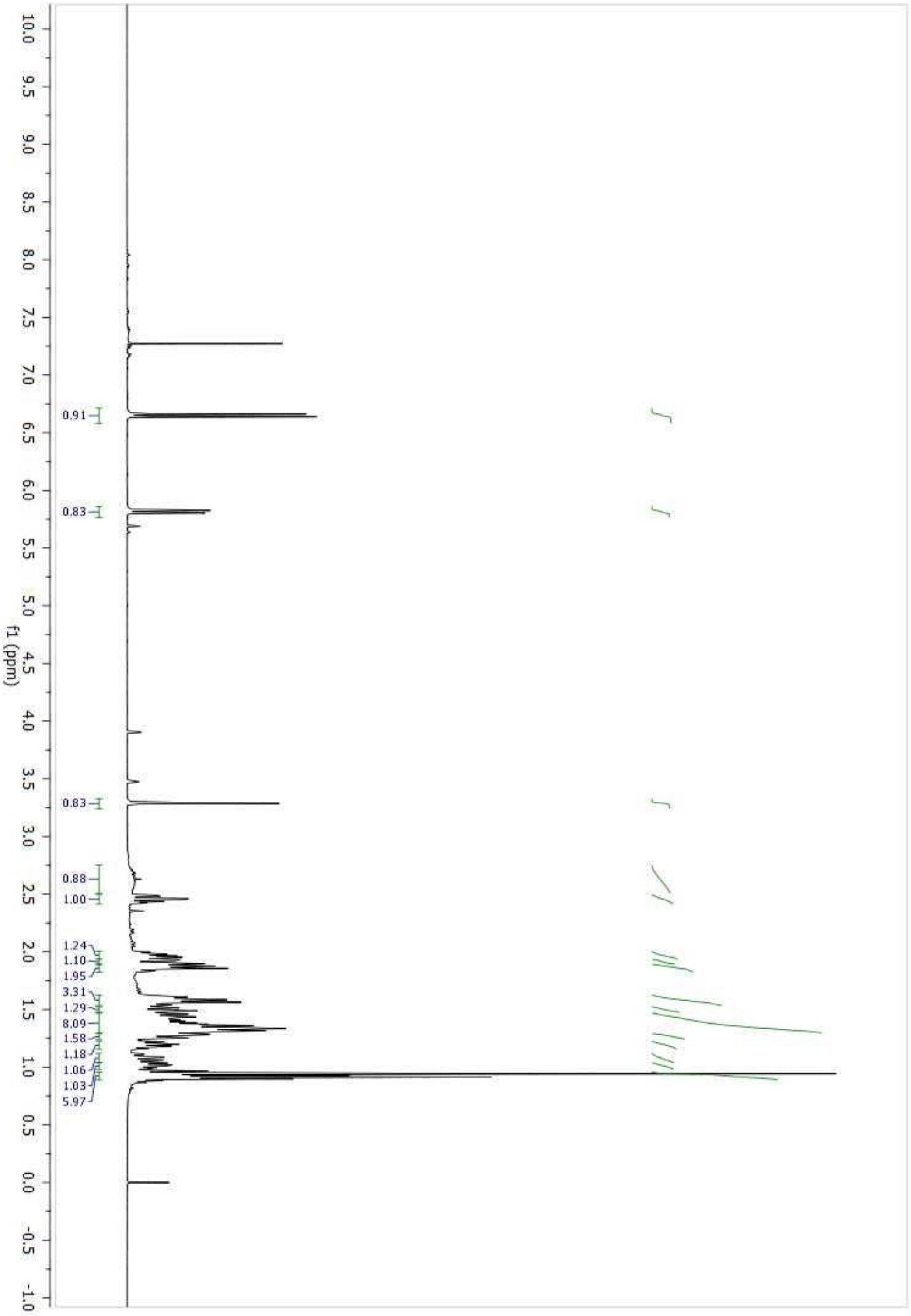

^{1}H NMR spectrum of compound**3f**(500 MHz, $CDCl_3$)

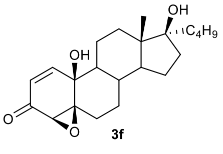

3f

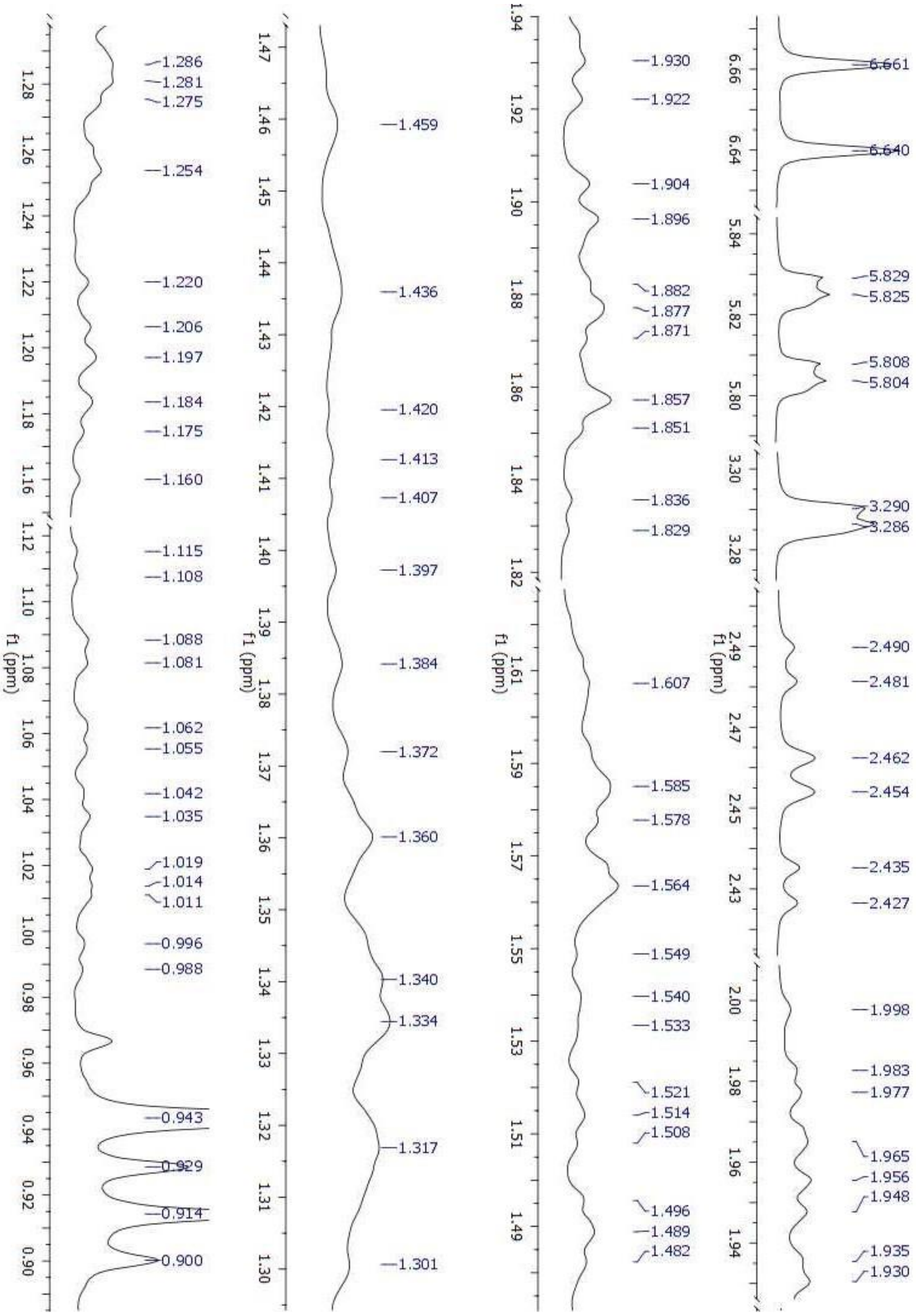

^{1}H NMR spectrum of compound**3f**(500 MHz, $CDCl_3$)

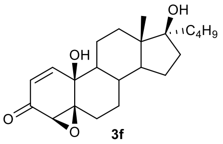

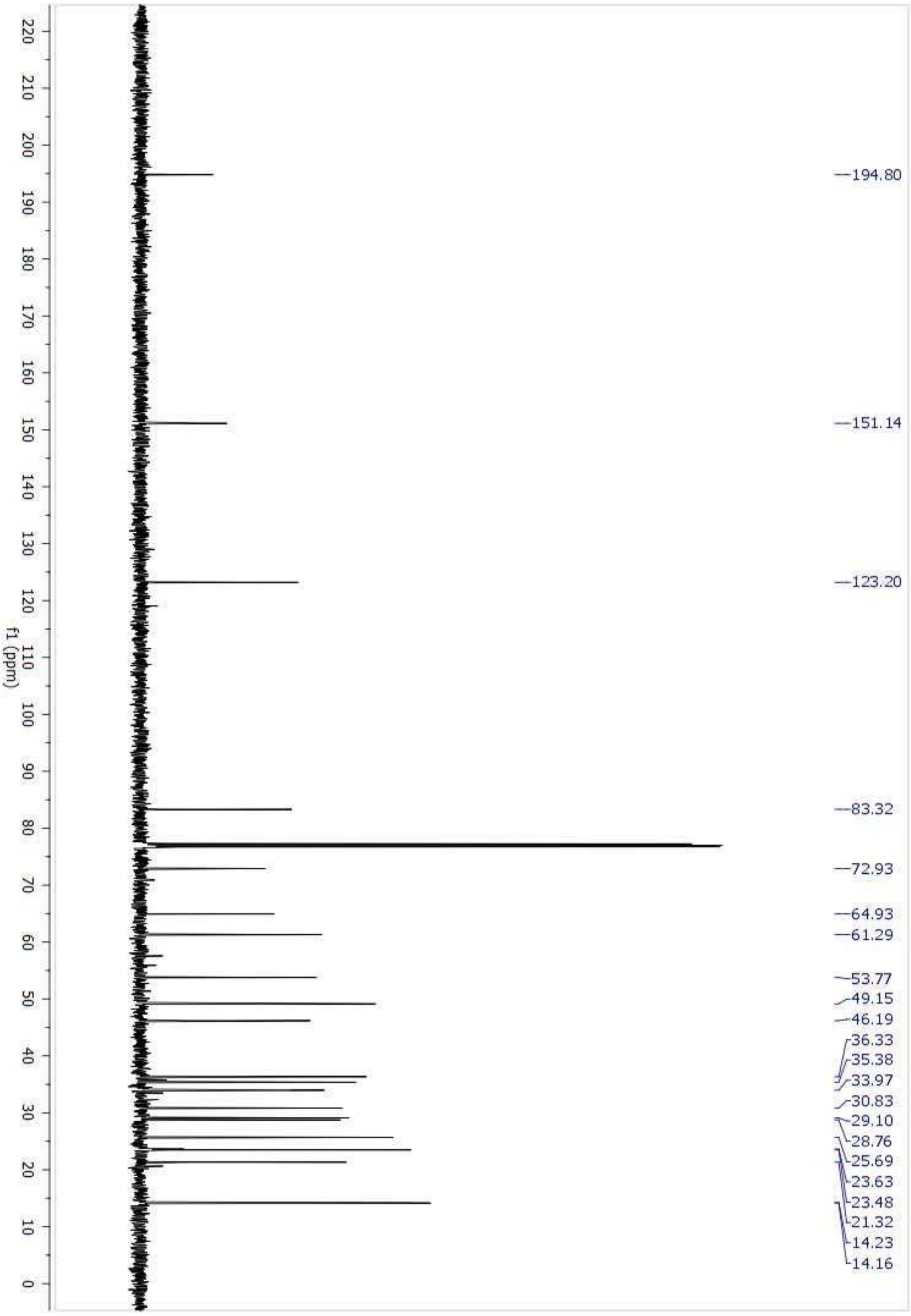

^{13}C NMR spectrum of compound **3f** (125 MHz, $CDCl_3$)

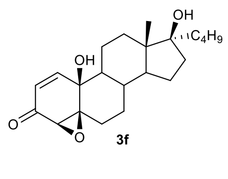

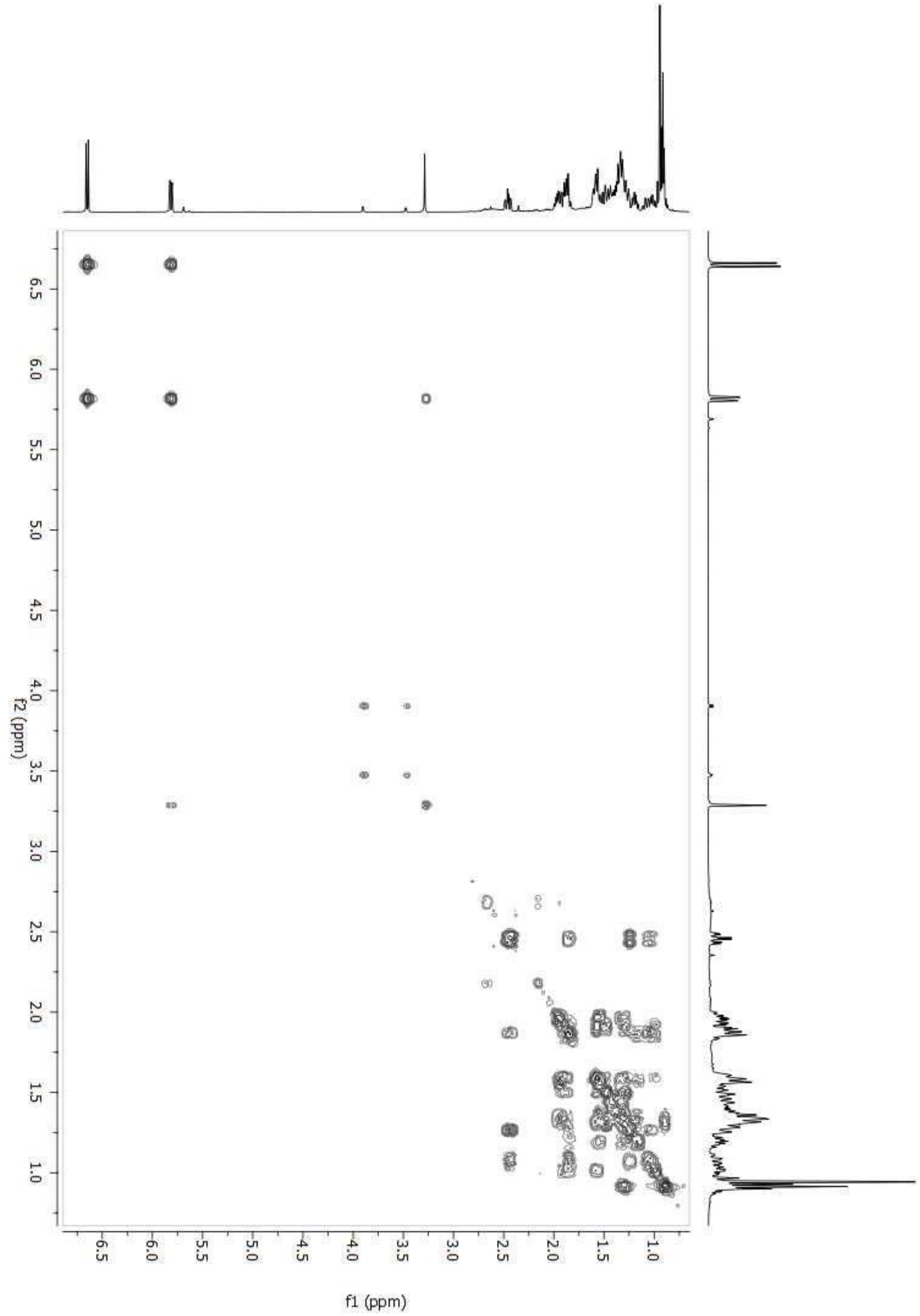

COSY spectrum of compound **3f**($CDCl_3$)

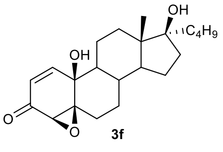

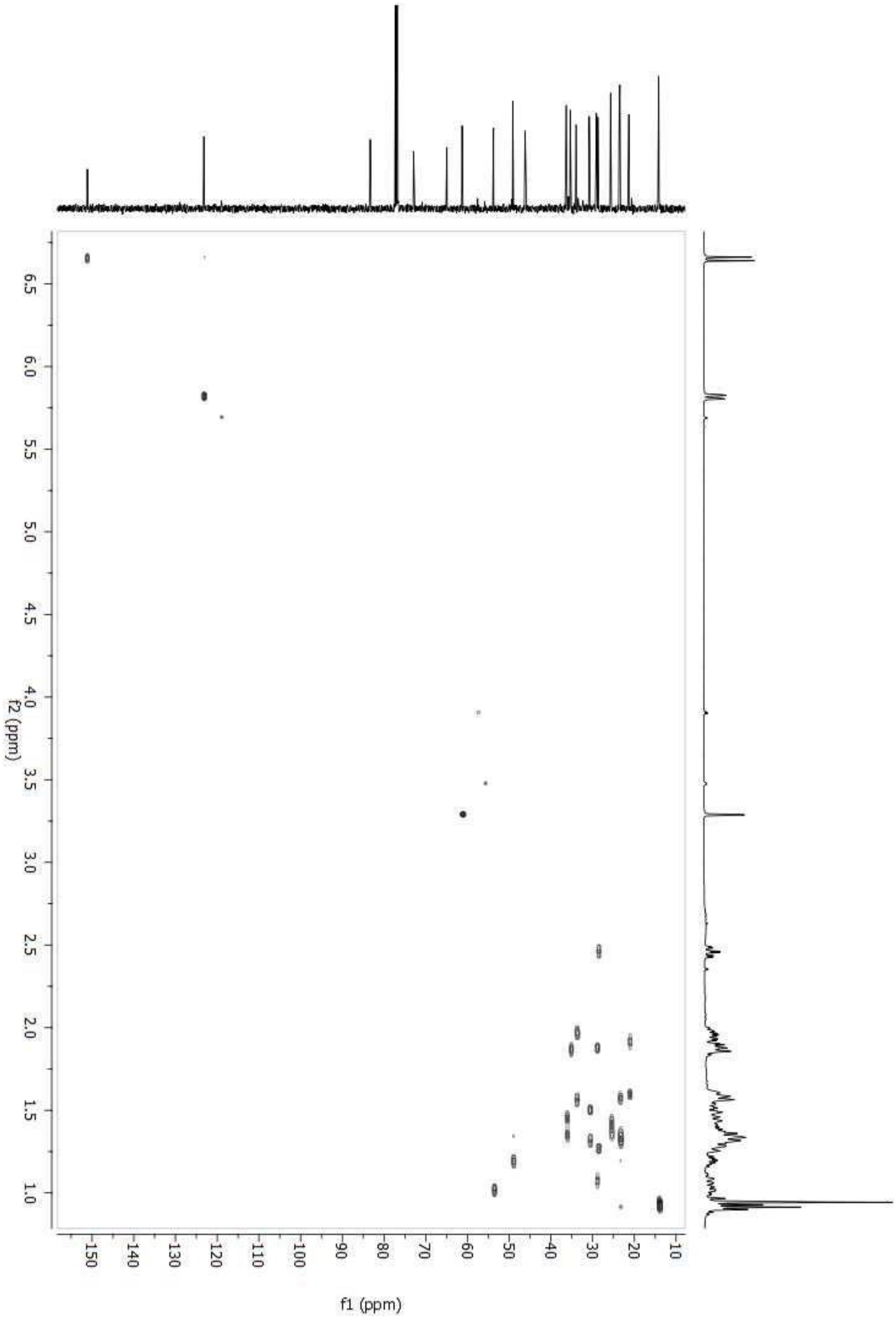

HSQC spectrum of compound **3f**($CDCl_3$)

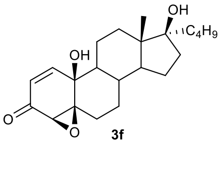

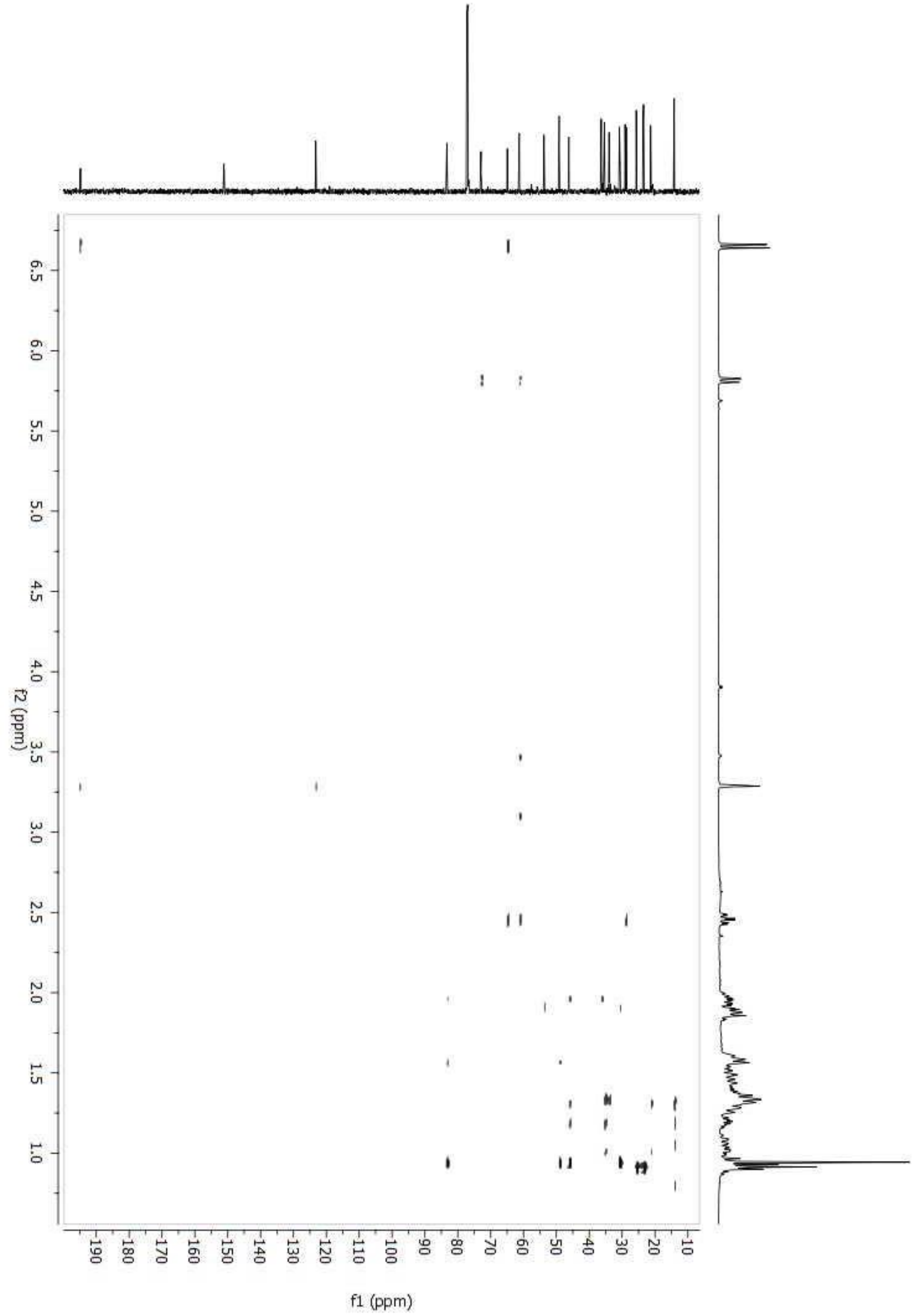

HMBC spectrum of compound **3f**($CDCl_3$)

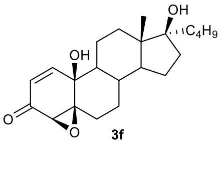

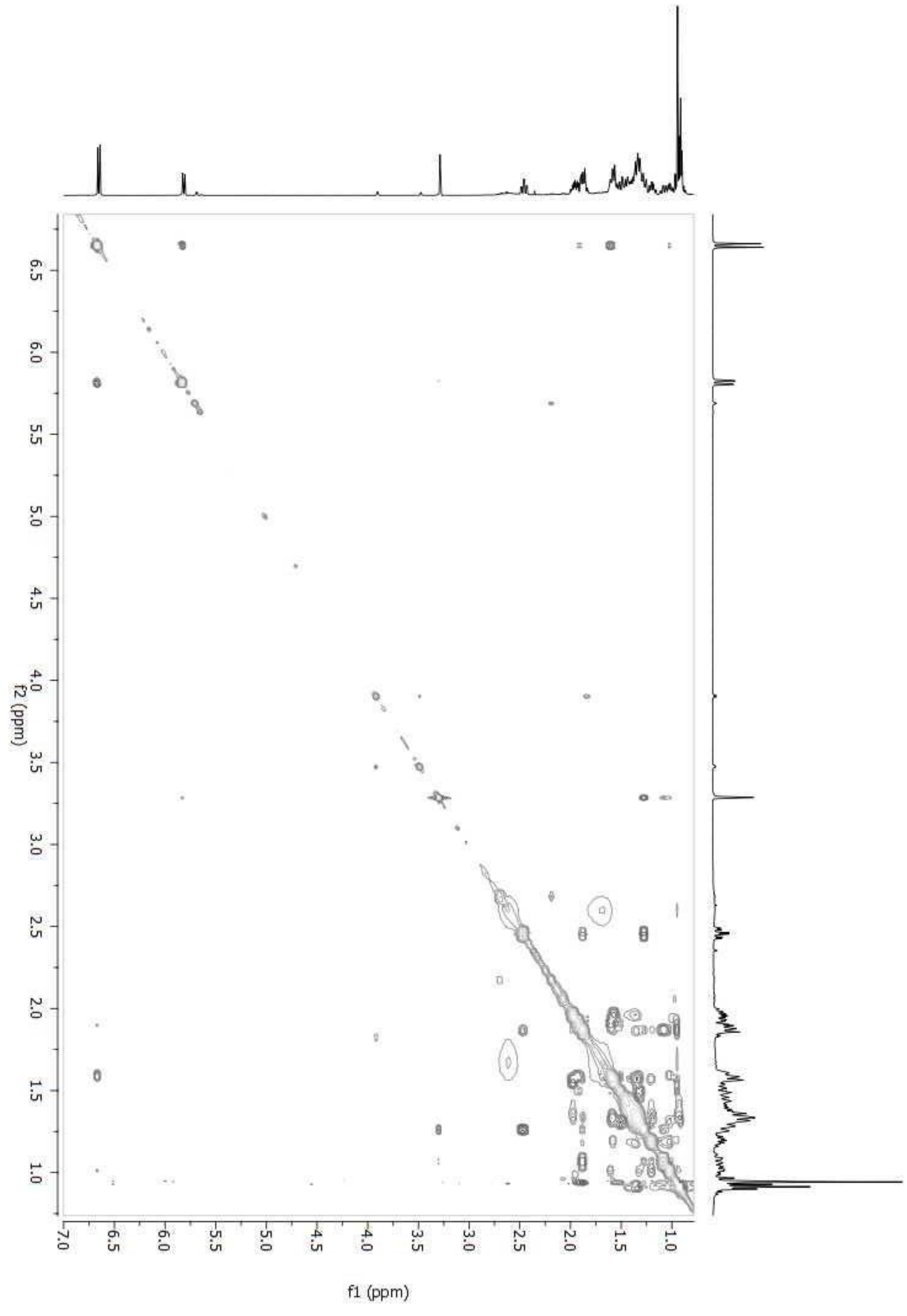

NOESY spectrum of compound **3f**($CDCl_3$)

3.9. Spectra of compound **4c** 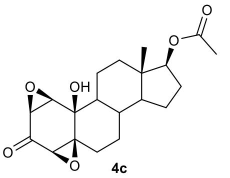 4c

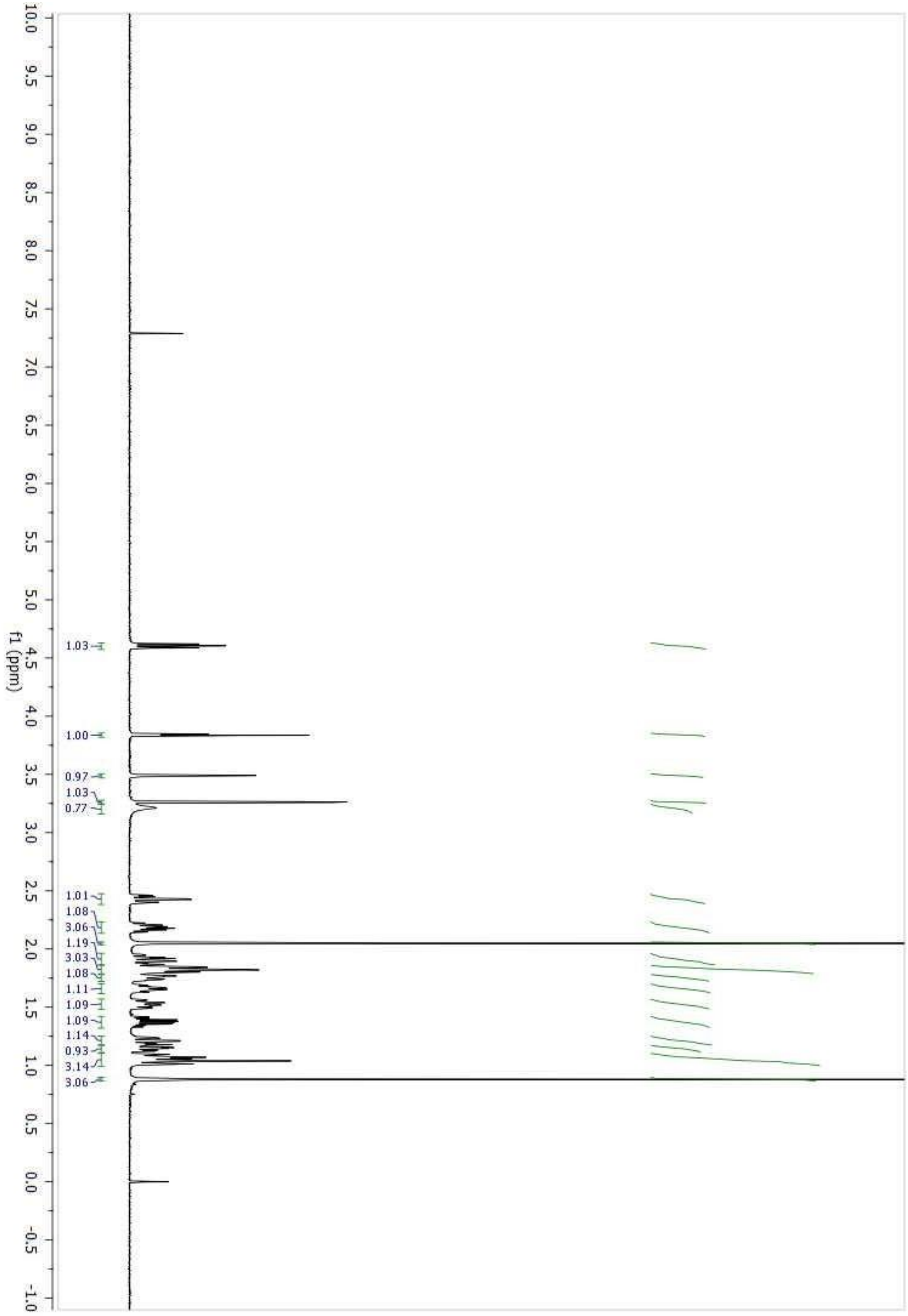

^{1}H NMR spectrum of compound**4c**(500 MHz, $CDCl_3$)

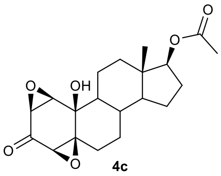

4c

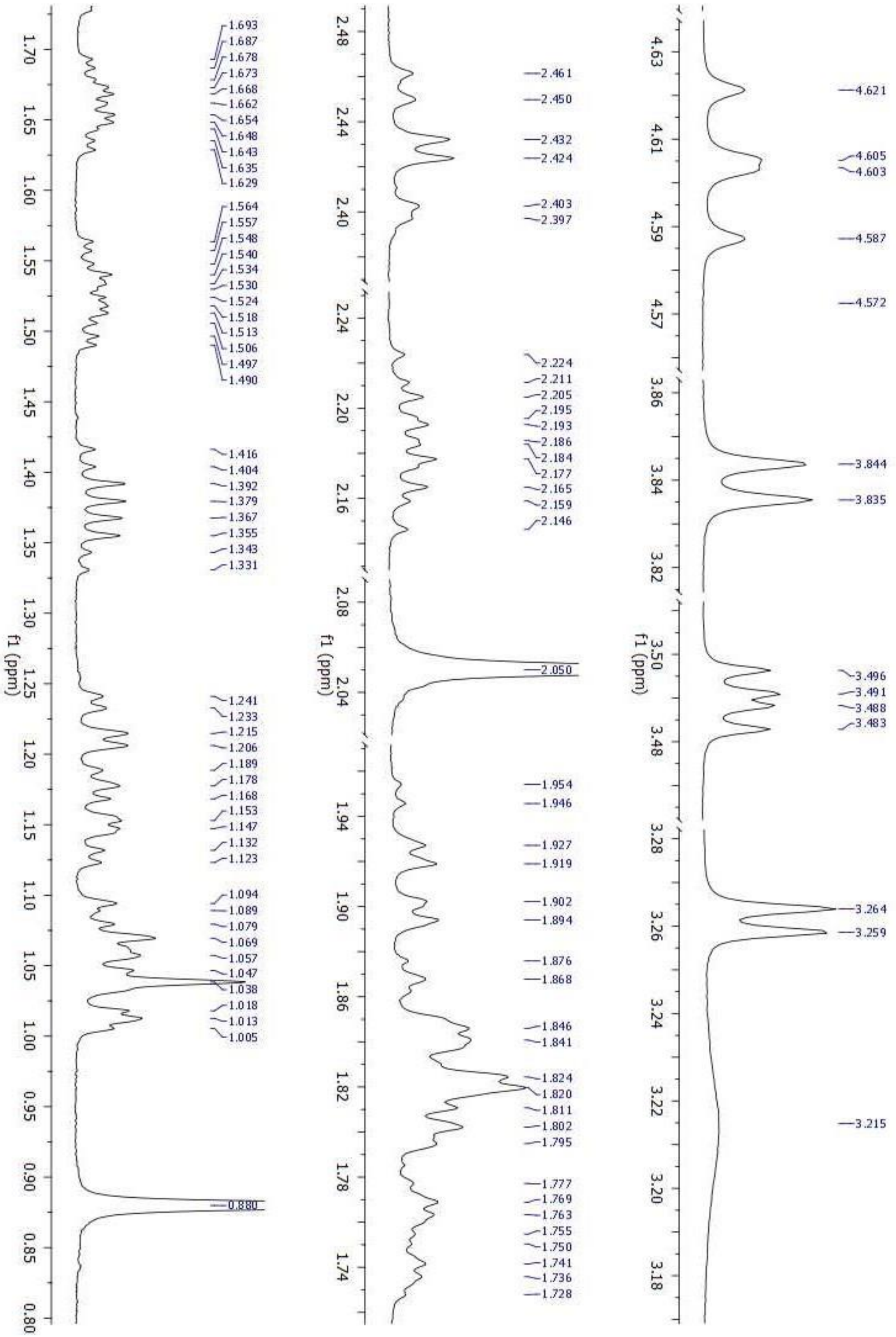

^{1}H NMR spectrum of compound **4c** (500 MHz, $CDCl_3$)

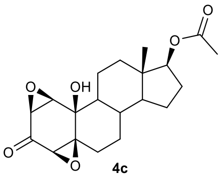

4c

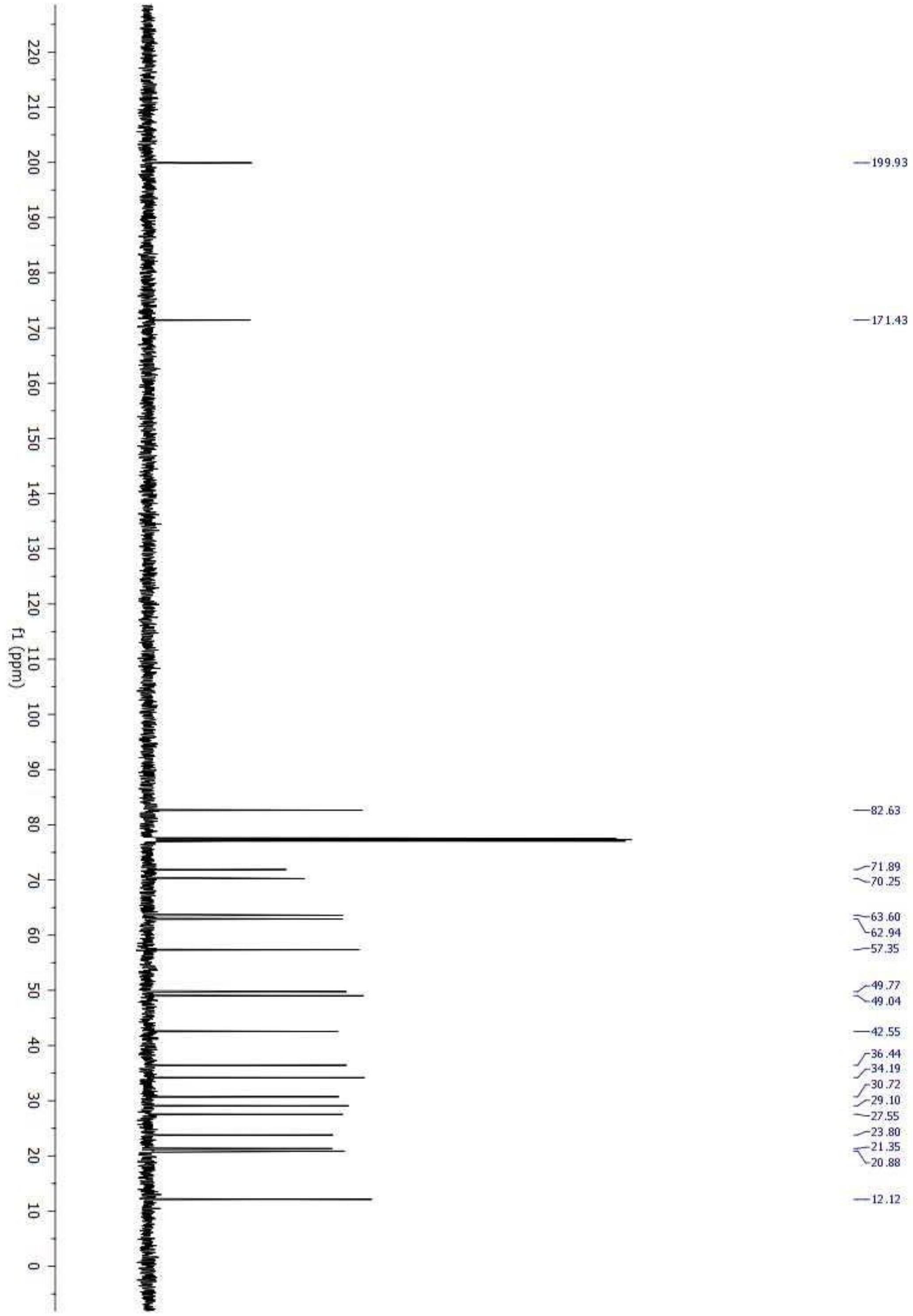

^{13}C NMR spectrum of compound **4c** (125 MHz, $CDCl_3$)

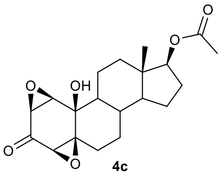

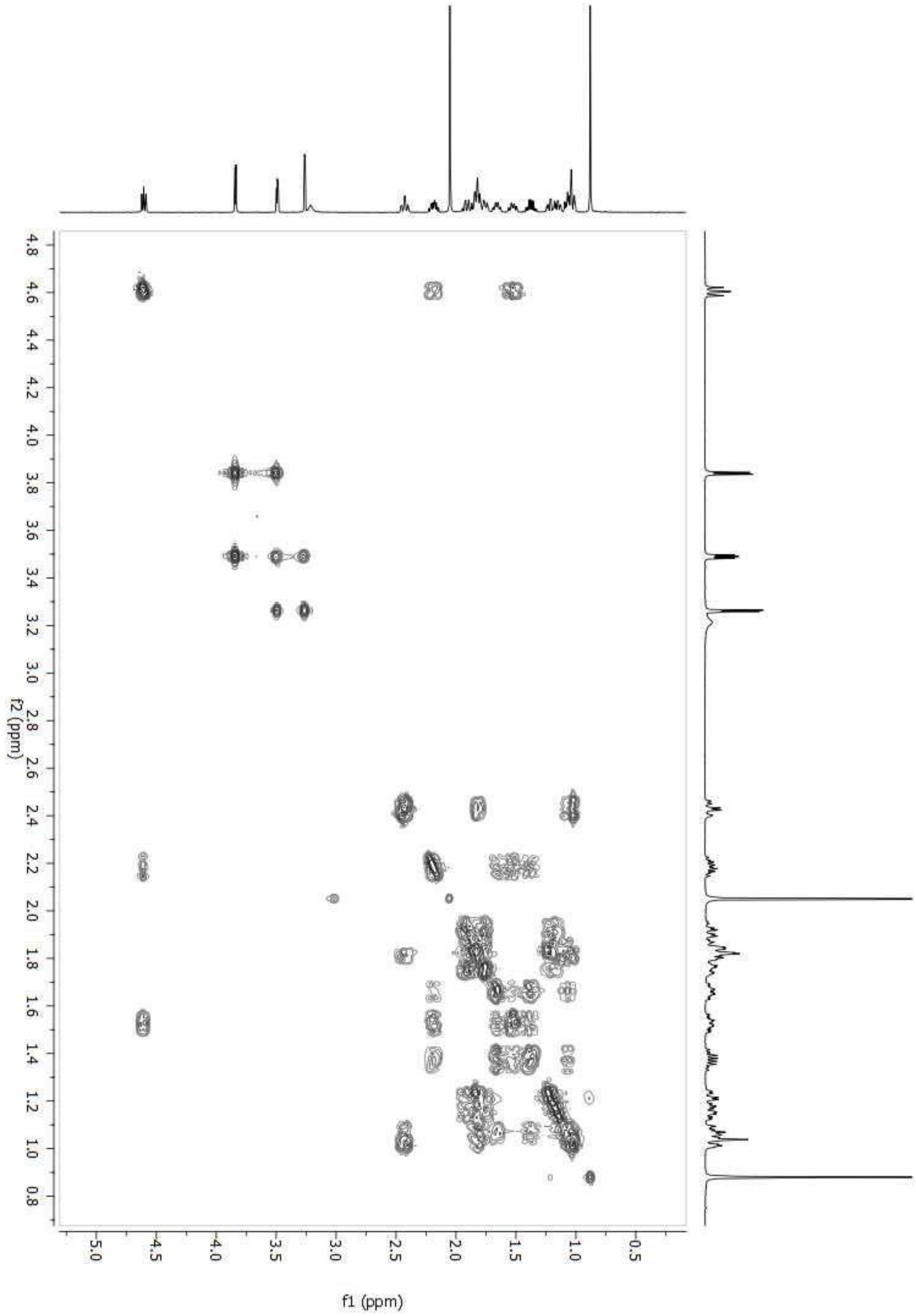

COSY spectrum of compound **4c**($CDCl_3$)

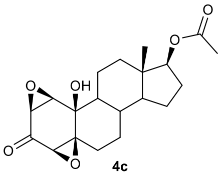

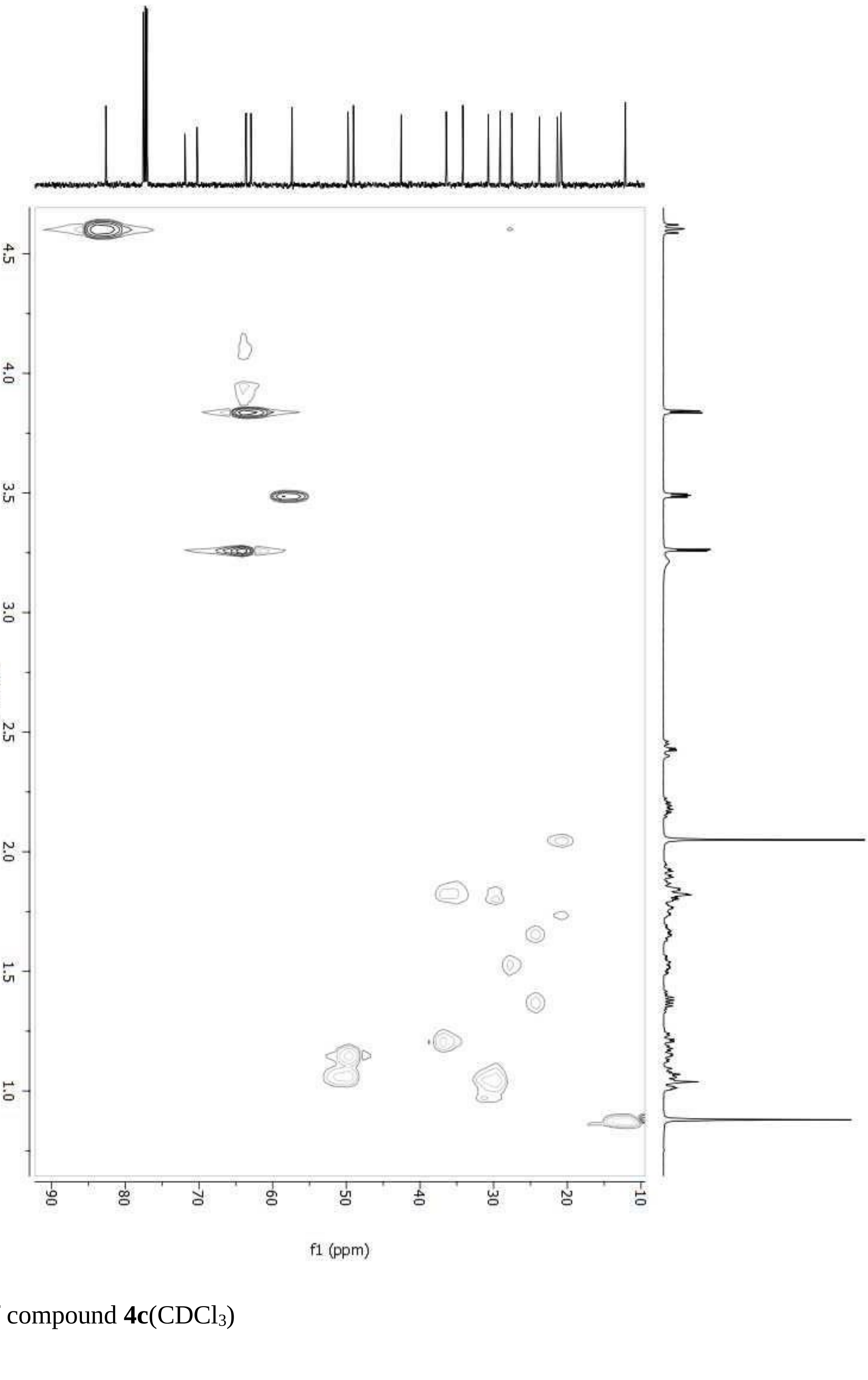

HSQC spectrum of compound **4c**($CDCl_3$)

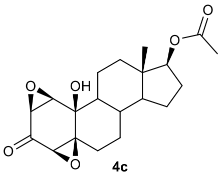

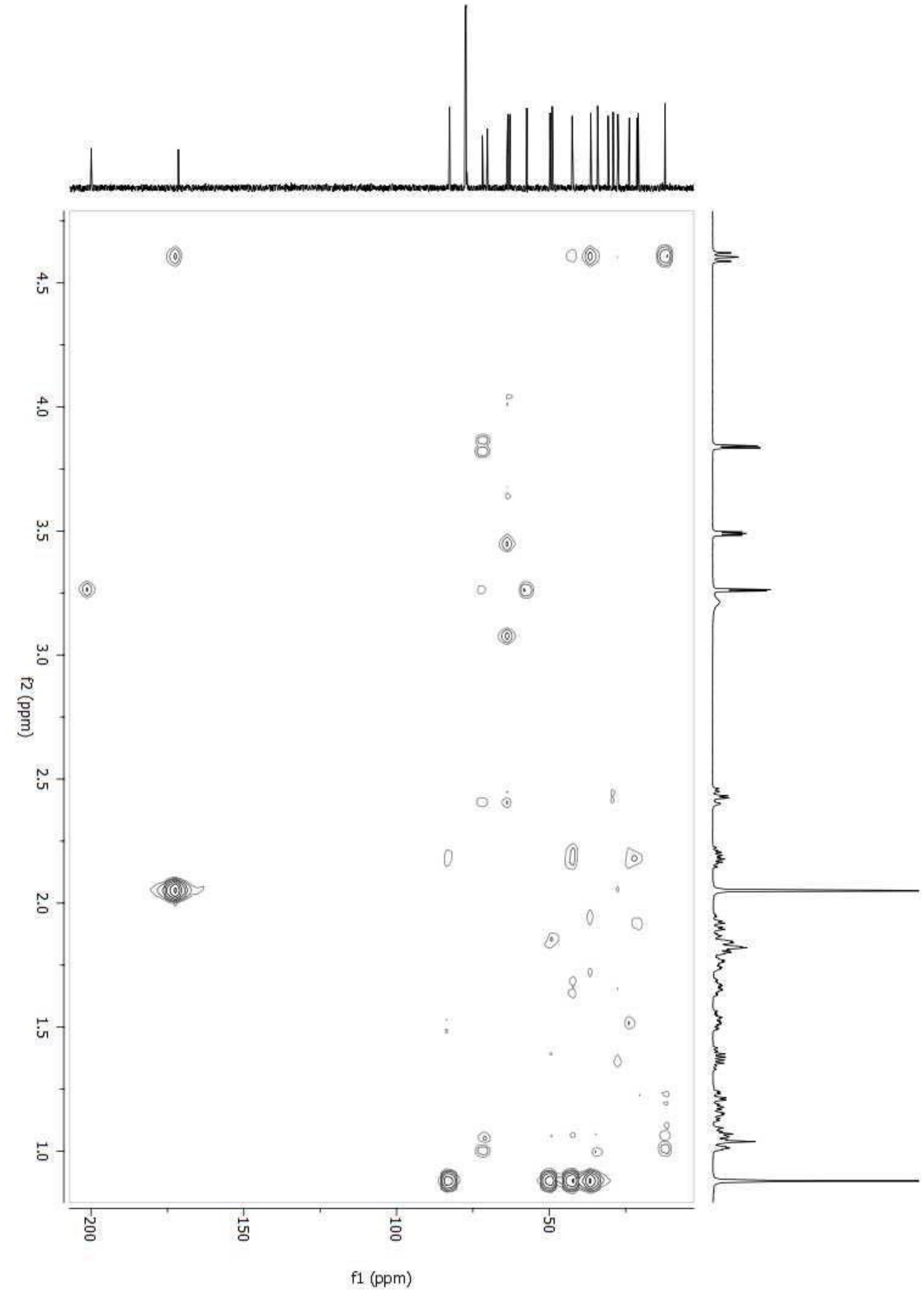

HMBC spectrum of compound **4c**($CDCl_3$)

3.10. Spectra of compound **4d** 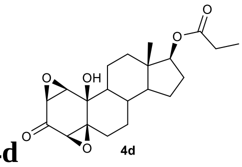

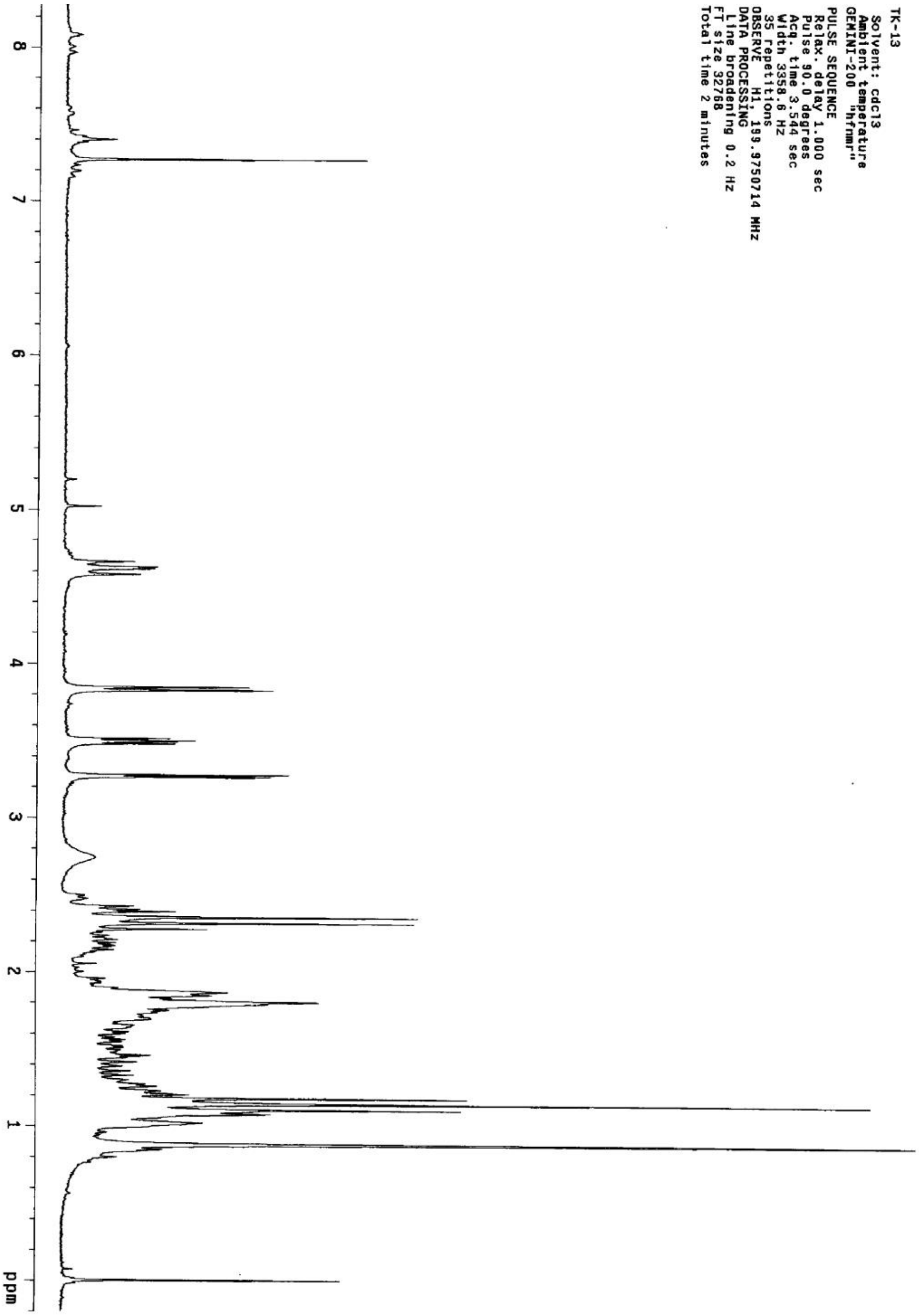

^{1}H NMR spectrum of compound**4d**(200 MHz, $CDCl_3$)

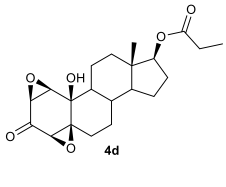

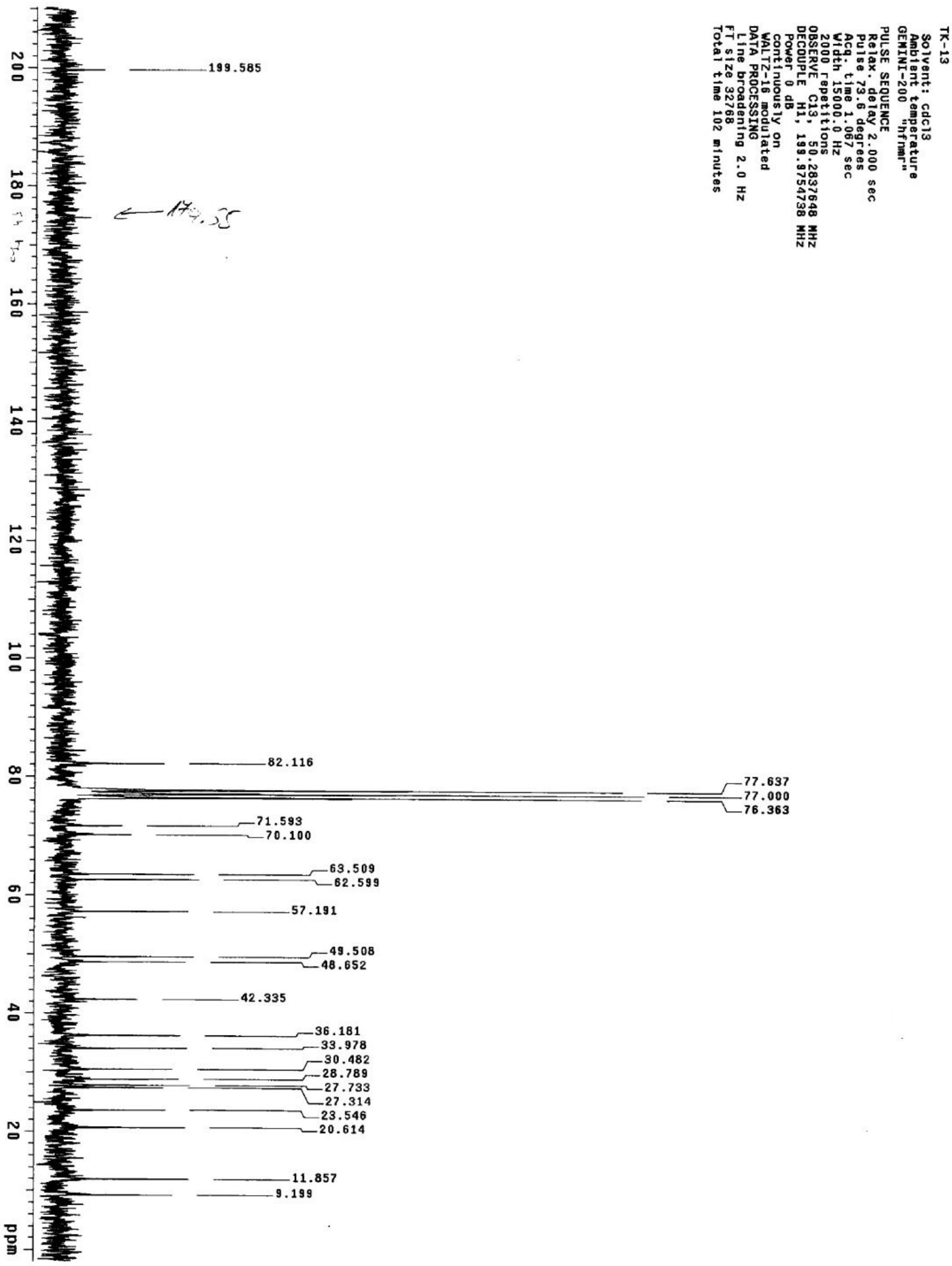

^{13}C NMR spectrum of compound**4d**(50 MHz, $CDCl_3$)

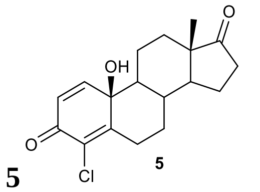

3.11. Spectra of compound **5**

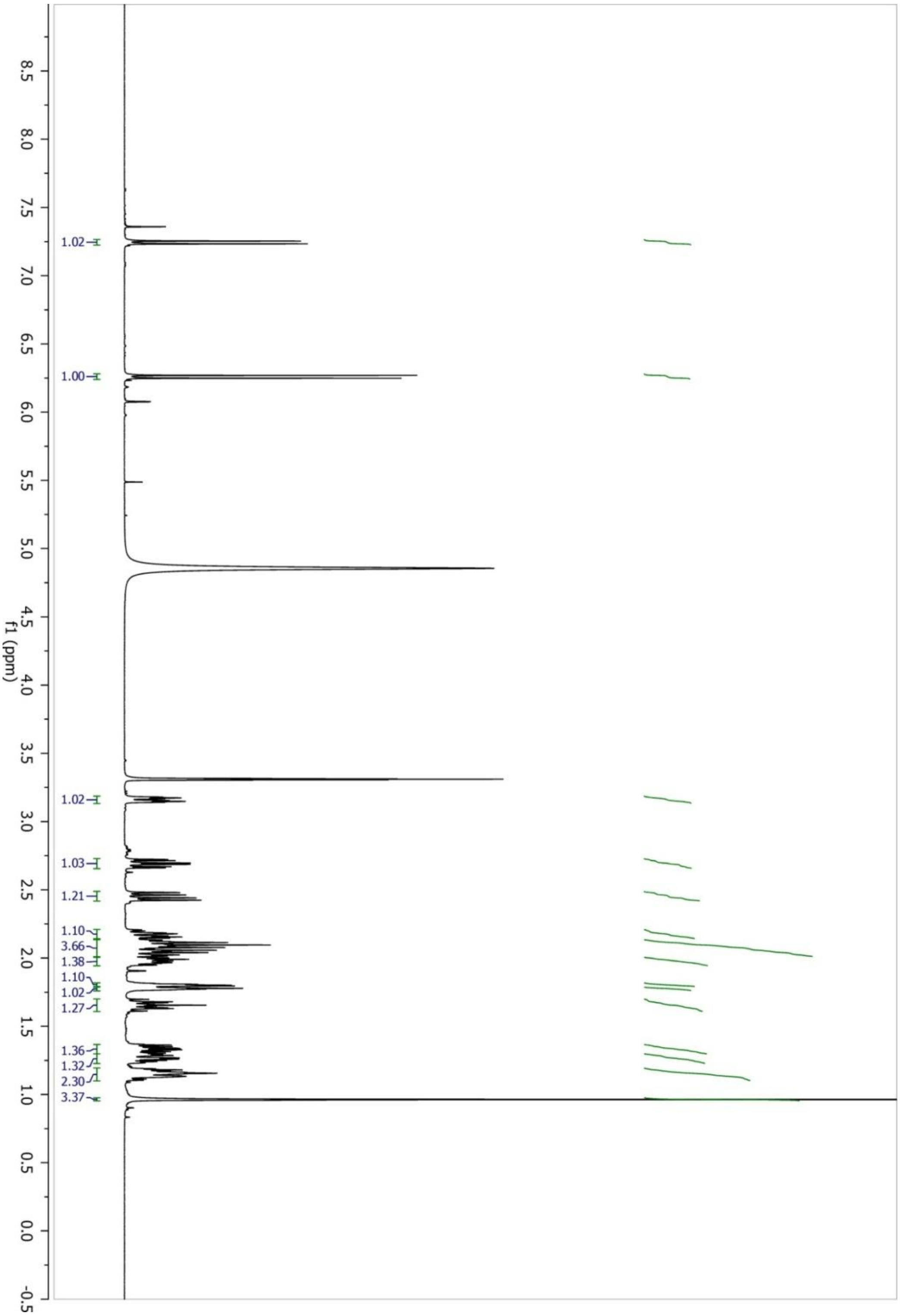

^{1}H NMR spectrum of compound**5**(500 MHz, Methanol-d_4)

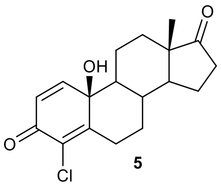

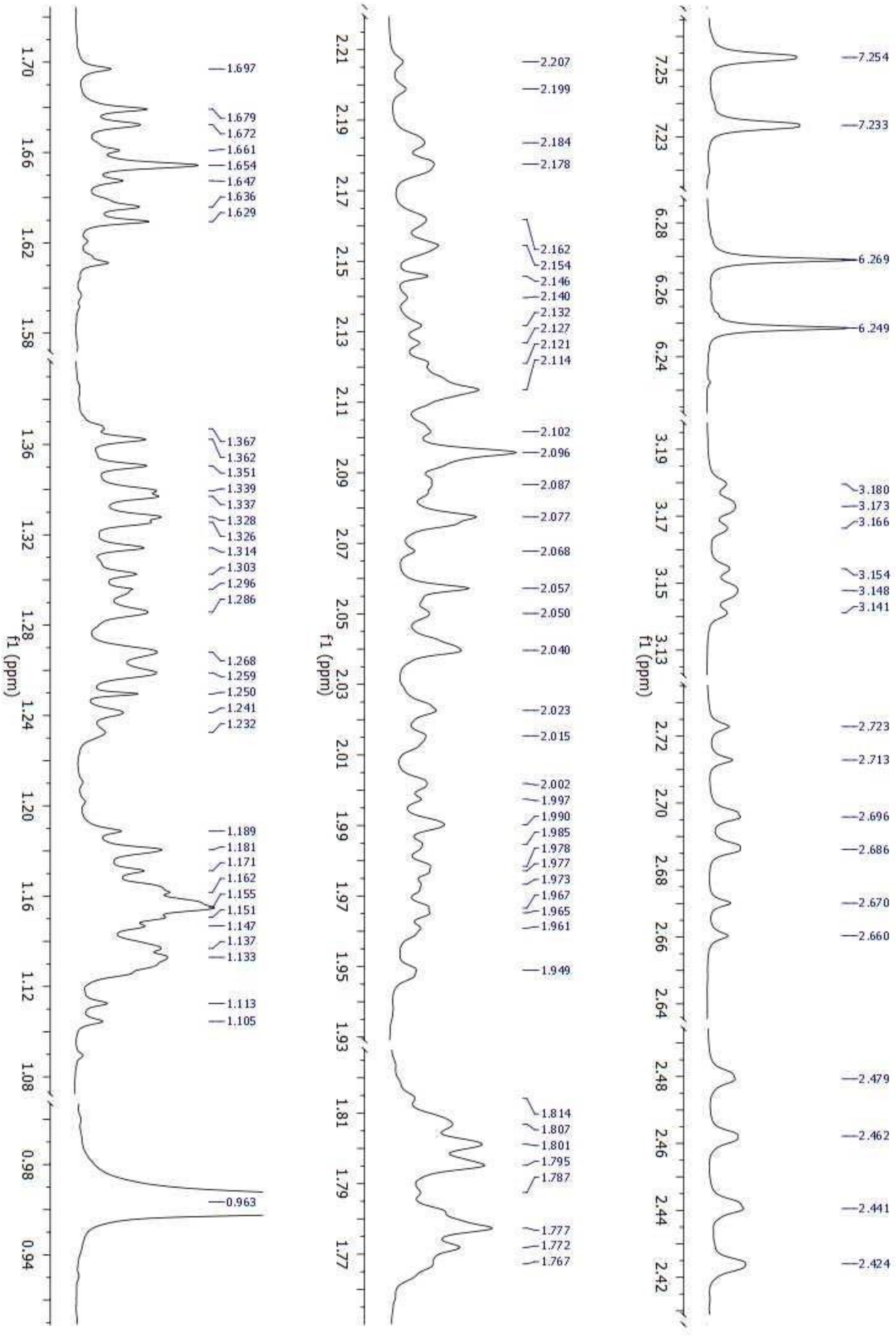

^{1}H NMR spectrum of compound**5**(500 MHz, Methanol-*d_4*)

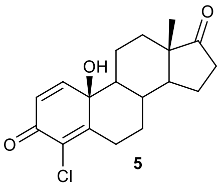

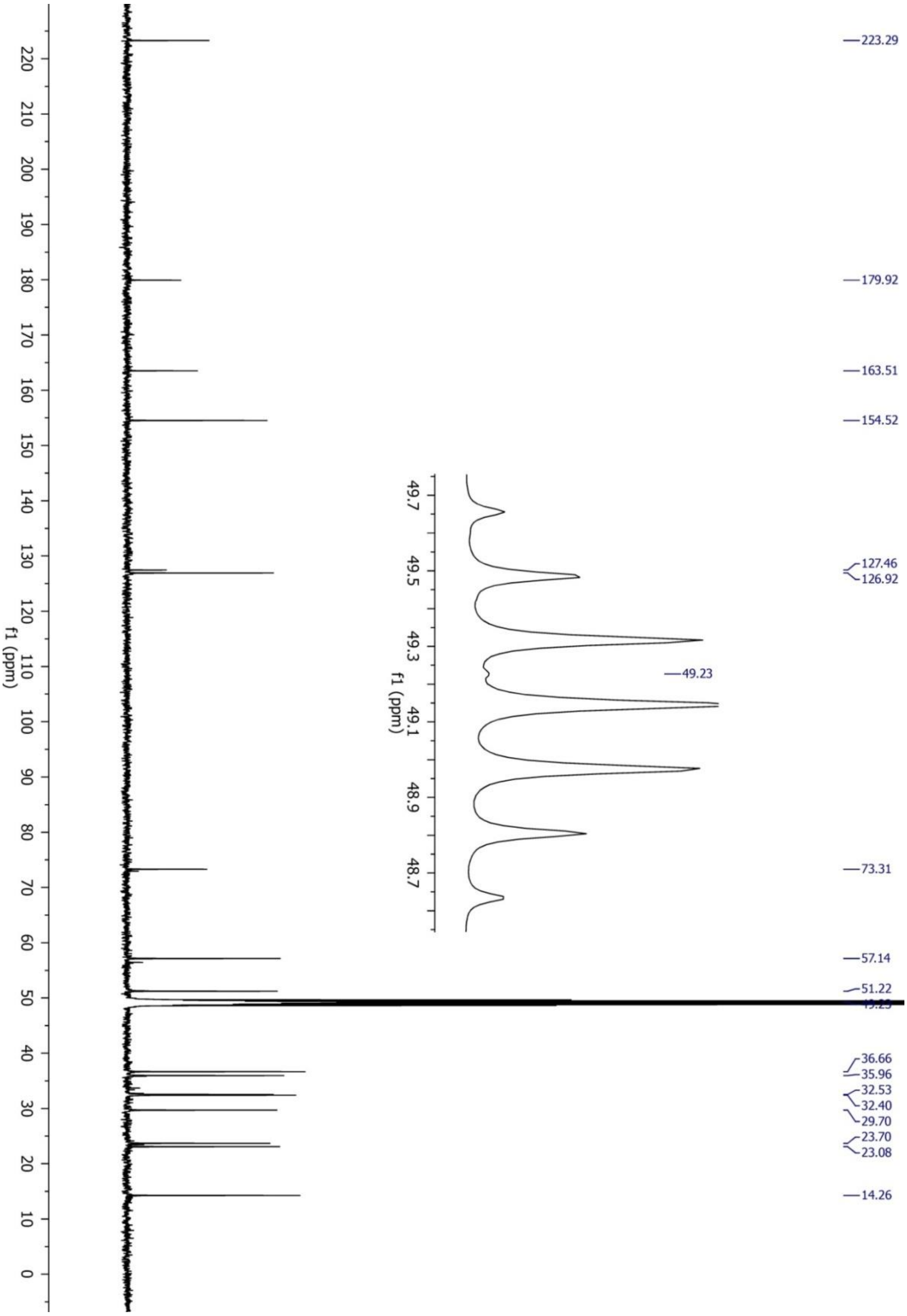

^{13}C NMR spectrum of compound**5**(125 MHz, Methanol-d_4)

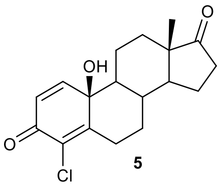

5

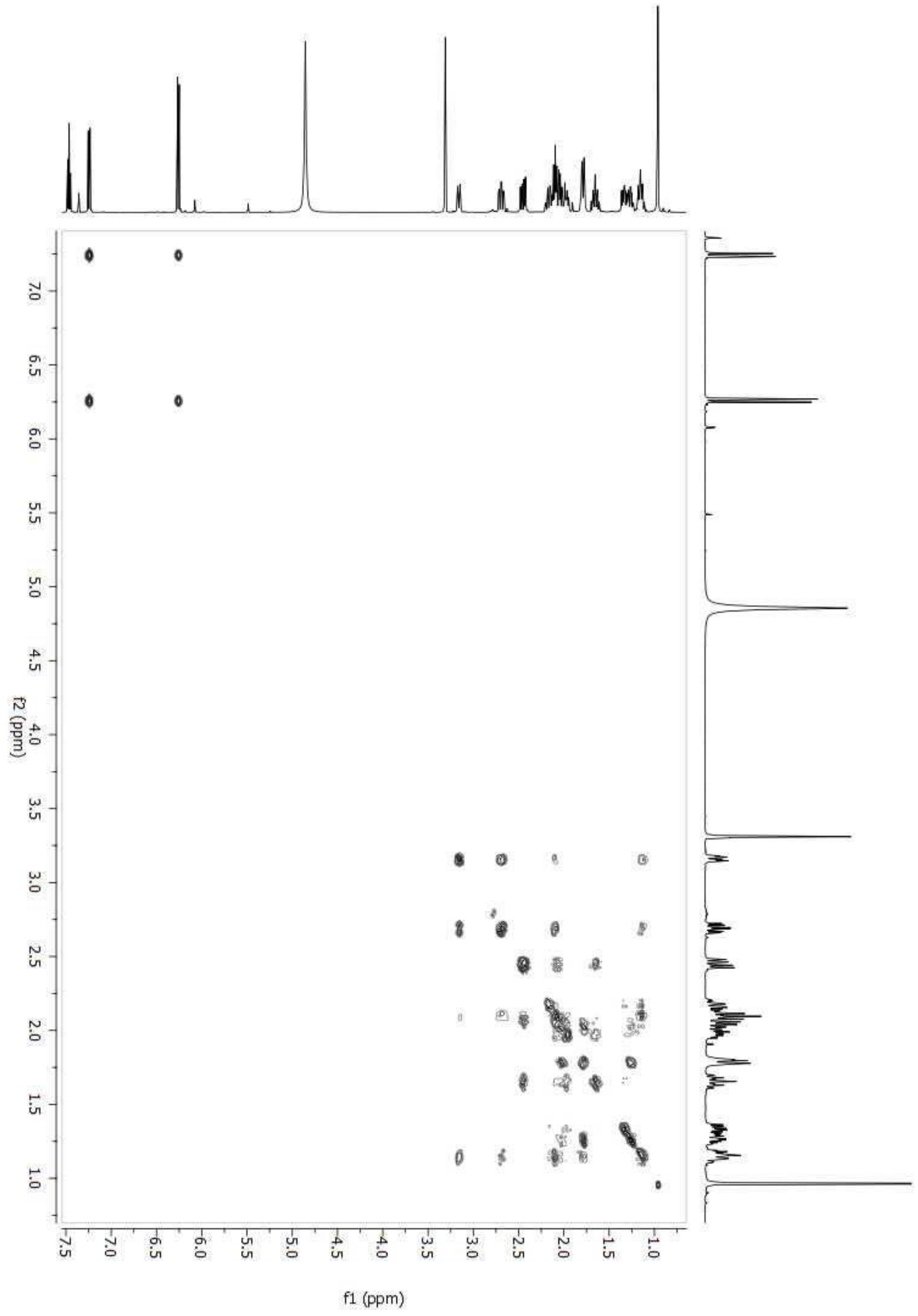

COSY spectrum of compound **5**(Methanol-*d₄*)

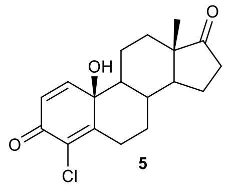

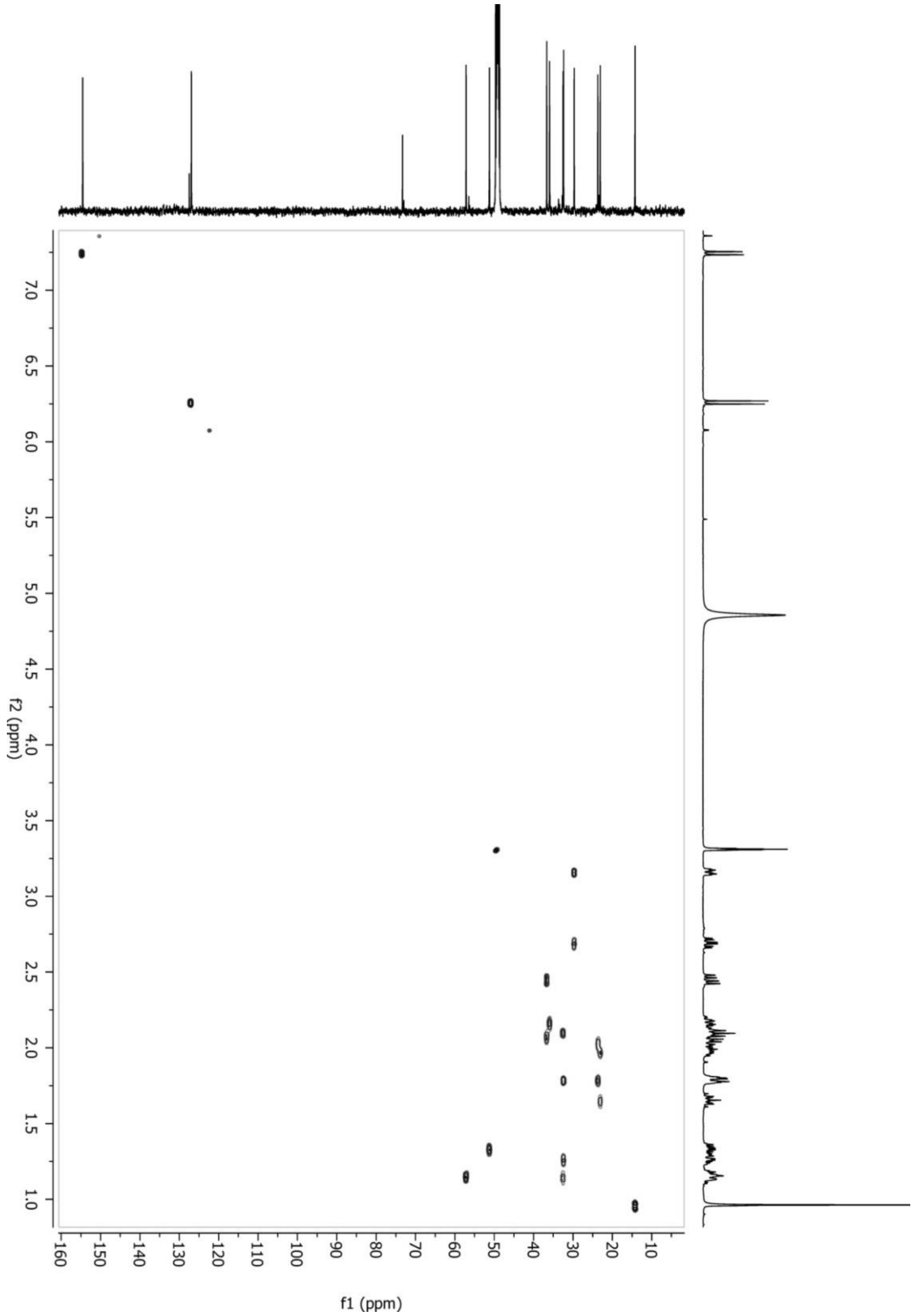

HSQC spectrum of compound **5**(Methanol-d_4)

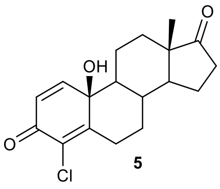

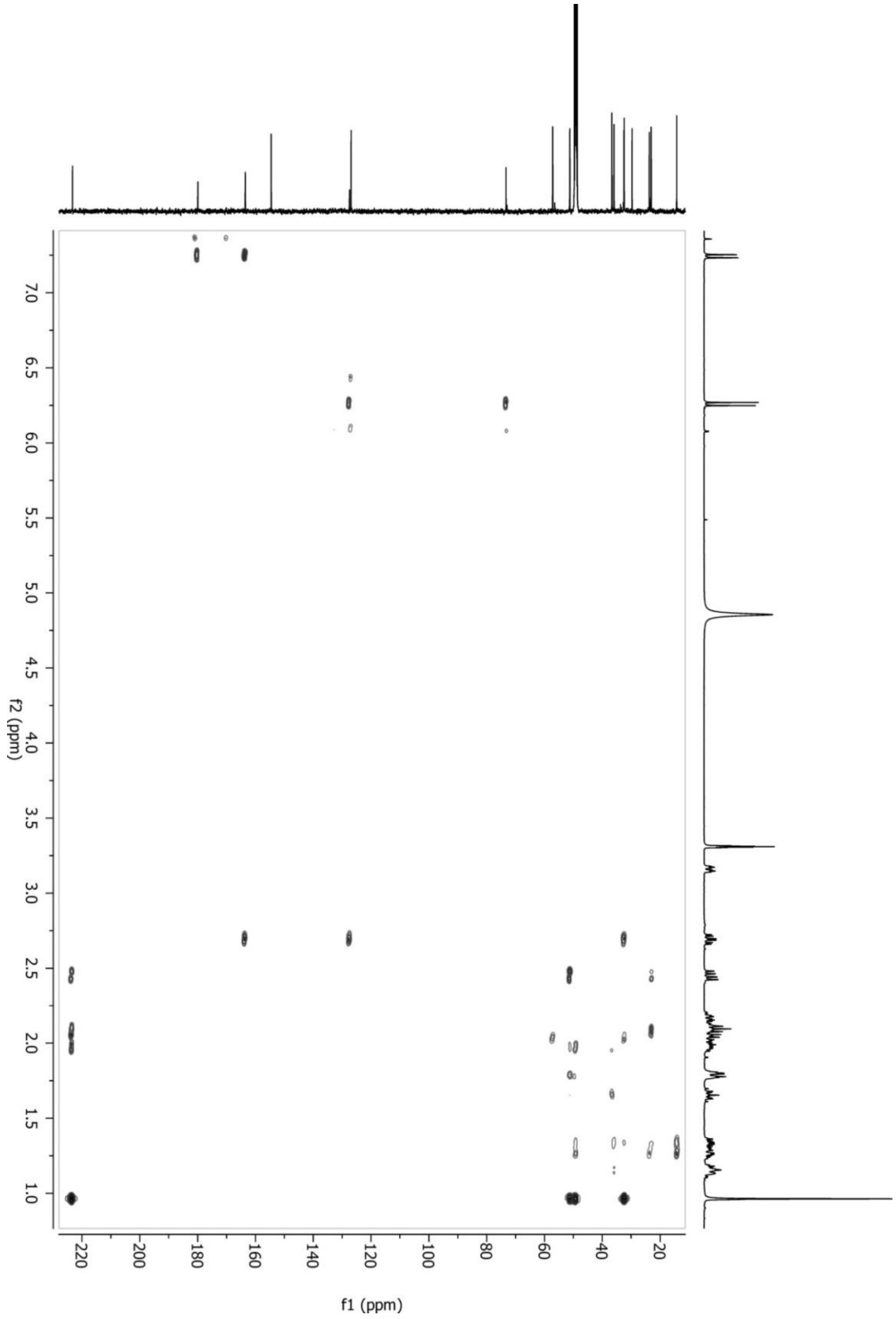

HMBC spectrum of compound **5**(Methanol-*d_4*)

3.12. Spectra of compound **6**

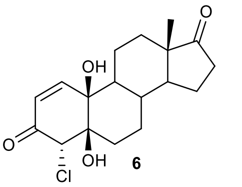

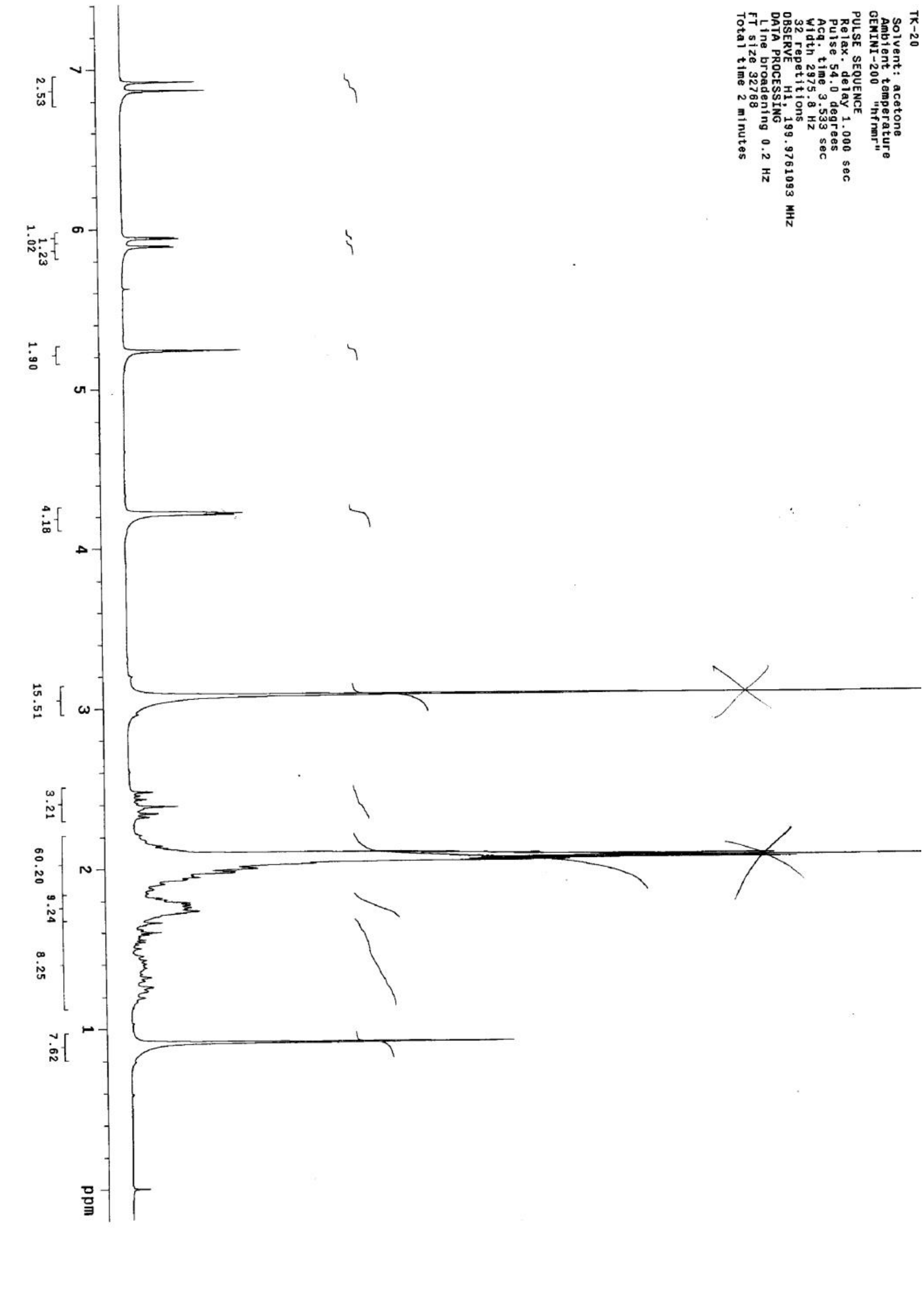

^{1}H NMR spectrum of compound**6**(200 MHz, Acetone-d_6)

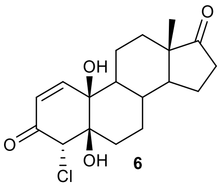

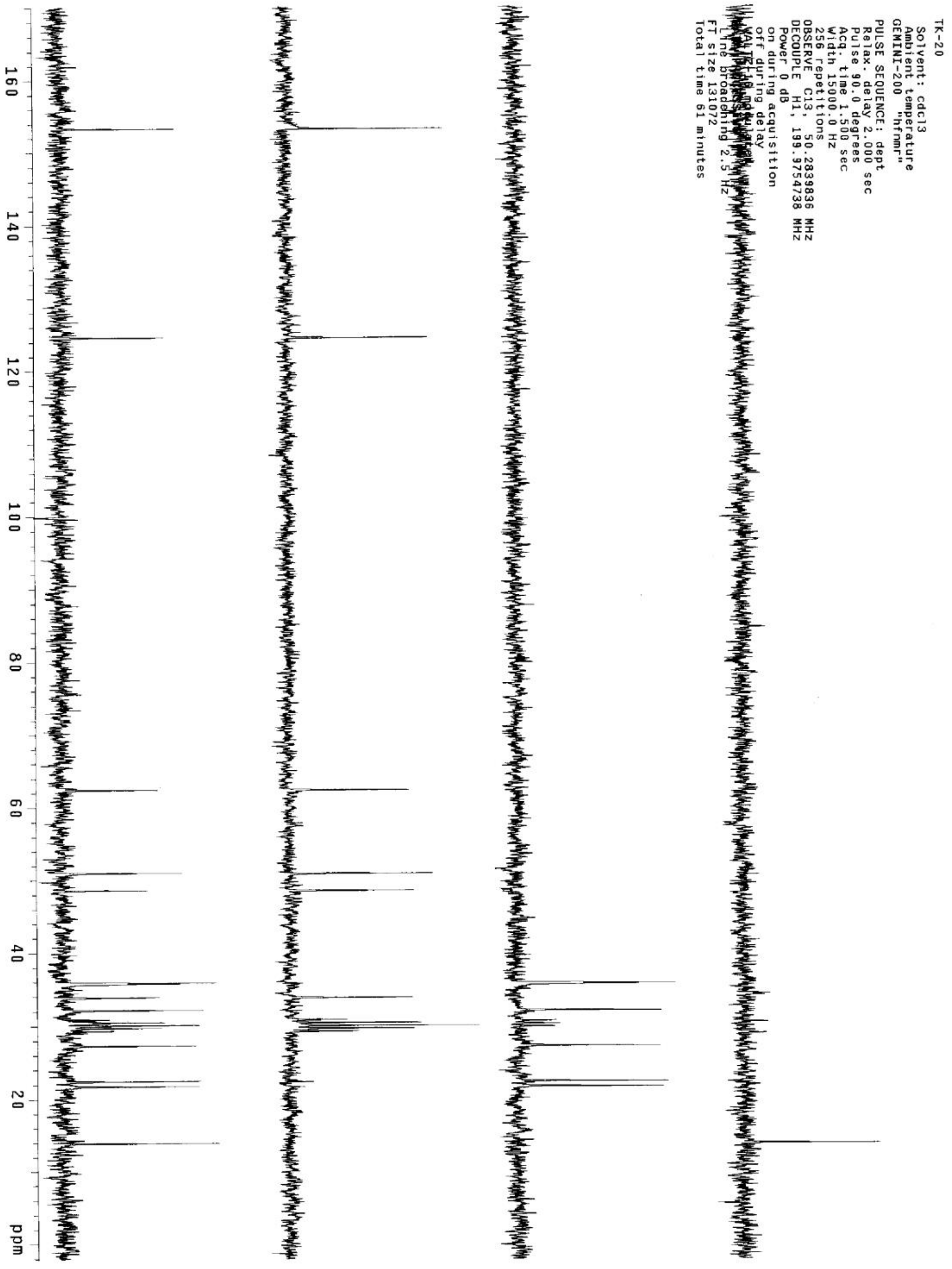

DEPT spectrum of compound **6**(50 MHz, Acetone-d_6)
